# Supplementary material for: A deimmunized and pharmacologically optimized Toll-like receptor 5 agonist for therapeutic applications
Source: Commun Biol. 2021 Apr 12;4:466. doi: 10.1038/s42003-021-01978-6 (PMC8041767; doi:10.1038/s42003-021-01978-6)
Supplement: Supplementary file 2 — Supplementary Information [file 42003_2021_1978_MOESM2_ESM.pdf]

SUPPLEMENTARY INFORMATION

A deimmunized and pharmacologically optimized Toll-like receptor 5 agonist for therapeutic applications

Vadim Mett[1], Oleg Kurnasov[2], Ivan Bespalov[3], Ivan Molodtsov[4], Craig Brackett[5], Lyudmila Burdelya[5], Andrei Purmal[3], Anatoli Gleiberman[3], Ilia Toshkov[3], Catherine Burkhart[1], Yakov Kogan[3], Ekaterina Andrianova[3], Andrei Gudkov[3,5,*], and Andrei Osterman[2]

[1] Buffalo BioLabs, LLC, Buffalo, NY, USA

[2] Sanford Burnham Prebys Medical Discovery Institute, La Jolla, CA, USA

[3] Genome Protection, Inc., Buffalo, NY, USA

[4] Gamaleya Research Center of Epidemiology and Microbiology, Moscow 123098, Russia

[5] Roswell Park Comprehensive Cancer Center, Buffalo, NY, USA

*corresponding author (andrei.gudkov@roswellpark.org)

Supplementary Figures 1 – 12

Supplementary Tables 1 – 8

Supplementary Methods:

Abzena Report 1
Abzena Report 2
Abzena Report 3

SUPPLEMENTARY FIGURES

```
                                       1         10          20
CBLB502 MRGSHHHHHHGMASMTGGQQMGRDLYDDDDKDPMAQVINTNSLSLLTQNNLNKSQSSLSS 60
        ←~~~~~~~~~N-terminal tag ~~~~~~~~~→←=========ND0 sub-domain=====

GP532   ------------------------------------------------------------

           30         40         50         60         70         80
CBLB502 AIERLSSGLRINSAKDDAAGQAIANRFTSNIKGLTQASRNANDGISIAQTTEGALNEINN 120
        =ND0→←~~~~~NS~~~~→←=====================ND1==================
GP532   -----MSGLRINSAKDDAAGQAAANRATSNIKGLTQASRNAADGISIAQTTEGALNEINN 55
              **************** *** ************** ******************

            90        100        110        120        130        140
CBLB502 NLQRVRELSVQATNGTNSDSDLKSIQDEIQQRLEEIDRVSNQTQFNGVKVLSQDNQMKIQ 180
        =============================ND1=================================
GP532   NLQRVRELSVQATAGANADAALKAIQAEIQQRLEEIDRVSQQTQAAAVKVLSQDNAMAIQ 115
        ************* * * *  ** ** ************* ***   ******** * **

           150        160        170                     402       410
CBLB502 VGANDGETITIDLQKIDVKSLGLDGFNVNSPGISGGGGGILDSMGTLINEDAAAAKKSTA 240
        =========ND1=================→←~loop/linker~~→←========CD1====
GP532   VGANDGAAITIDLQKIDVKSLGLDGFNVNSPG-------------------------STA 150
        ******  ************************                         ***

           420        430        440        450        460        470
CBLB502 NPLASIDSALSKVDAVRSSLGAIQNRFDSAITNLGNTVTNLNSARSRIEDADYATEVSNM 300
        ================CD1===============================→←~CS~→←=CD0
GP532   NPLASIDSALSKVDAVRSSLGAIQNRFDSAITNLGNTVTNLNSARSRIEDADYATEVSQM 210
        **********************************************************:*
            480         490        500
CBLB502 SKAQILQQAGTSVLAQANQVPQNVLSLLR 329
        =========CD0=================→
GP532   SKAQILDQAGTSTLAQLVPRGSHHHHHHG 239
        ****** ***** ***←~C-term.tag~→
```

Supplementary Figure 1. Amino acid (aa) sequence alignment of the partially deimmunized and pharmacologically optimized TLR5 agonist GP532 with the parental drug entolimod (CBLB502). Numbering by *Salmonella enterica* serovar Dublin flagellin (FliC) is shown above each sequence block; numbering by CBLB502 and GP532 is shown to the right of the respective sequences.

Sequences originating from flagellin are shown in black bold font and include:

- N-terminal segment (sub-domain) of D0 helical domain (ND0: aa 1 – 33 in FliC);
- Unstructured "spoke" region connecting ND0 and ND1 segments (NS: aa 34-45 in FliC);
- N-terminal segment (sub-domain) of D1 helical domain (ND1: aa 45 – 176 in FliC);
- C-terminal segment (sub-domain) of D1 helical domain (ND1: aa 402 – 463 in FliC);
- Unstructured "spoke" region connecting CD1 and CD0 segments (CS: aa 464 – 469 in FliC);
- C-terminal segment (sub-domain) of D0 helical domain (ND0: aa 470 – 504 in FliC).

Artificial segments are shown in non-bold italic font and include:

- N-terminal tag of CBLB502 (33 aa), which includes a His_6 tag (green highlight) and an enterokinase cleavage site (blue highlight);
- Flexible linker (yellow highlight) replacing domains D2 and D3 of FliC (comprised of 16 aa in CBLB502 but only three aa in GP532);
- C-terminal tag of GP532 (13 aa), which replaces 13 deleted C-terminal residues of FliC and includes a thrombin cleavage site (blue highlight) and a His_6 tag (green highlight).

Amino acid substitutions in GP532 vs. CBLB502 are shown in red.

```
CBLB502 MRGSHHHHHHGMASMTGGQQMGRDLYDDDDKDPMAQVINTNSLSLLTQNNLNKSQSSLSS 60
33ML    ------------------------------------------------------------
33MX    ------------------------------------------------------------
GP532   ------------------------------------------------------------


CBLB502 AIERLSSGLRINSAKDDAAGQAIANRFTSNIKGLTQASRNANDGISIAQTTEGALNEINN 120
33ML    -----MSGLRINSAKDDAAGQAIANRFTSNIKGLTQASRNANDGISIAQTTEGALNEINN 55
33MX    -----MSGLRINSAKDDAAGQAIANRFTSNIKGLTQASRNAADGISIAQTTEGALNEINN 55
GP532   -----MSGLRINSAKDDAAGQAAANRATSNIKGLTQASRNAADGISIAQTTEGALNEINN 55
              **************** *** ************** ******************

CBLB502 NLQRVRELSVQATNGTNSDSDLKSIQDEIQQRLEEIDRVSNQTQFNGVKVLSQDNQMKIQ 180
33ML    NLQRVRELSVQATNGTNSDSDLKSIQDEIQQRLEEIDRVSNQTQFNGVKVLSQDNQMKIQ 115
33MX    NLQRVRELSVQATAGANADAALKAIQAEIQQRLEEIDRVSQQTQAAAVKVLSQDNAMAIQ 115
GP532   NLQRVRELSVQATAGANADAALKAIQAEIQQRLEEIDRVSQQTQAAAVKVLSQDNAMAIQ 115
        ************* *:*:*: **:** *************:***  .******** * **

CBLB502 VGANDGETITIDLQKIDVKSLGLDGFNVNSPGISGGGGGILDSMGTLINEDAAAAKKSTA 240
33ML    VGANDGETITIDLQKIDVKSLGLDGFNVNSPG-------------------------STA 150
33MX    VGANDGAAITIDLQKIDVKSLGLDGFNVNSPG-------------------------STA 150
GP532   VGANDGAAITIDLQKIDVKSLGLDGFNVNSPG-------------------------STA 150
        ****** :************************                         ***

CBLB502 NPLASIDSALSKVDAVRSSLGAIQNRFDSAITNLGNTVTNLNSARSRIEDADYATEVSNM 300
33ML    NPLASIDSALSKVDAVRSSLGAIQNRFDSAITNLGNTVTNLNSARSRIEDADYATEVSNM 210
33MX    NPLASIDSALSKVDAVRSSLGAIQNRFDSAITNLGNTVTNLNSARSRIEDADYATEVSQM 210
GP532   NPLASIDSALSKVDAVRSSLGAIQNRFDSAITNLGNTVTNLNSARSRIEDADYATEVSQM 210
        **********************************************************:*

CBLB502 SKAQILQQAGTSVLAQANQVPQNVLSLLR           329
33ML    SKAQILQQAGTSVLAQANQVPQNVLSLLVPRGSHHHHHHG 250
33MX    SKAQILQQAGTSVLAQANQVPQNVLSLLVPRGSHHHHHHG 250
GP532   SKAQILDQAGTSTLAQ           LVPRGSHHHHHHG 239
        ******:*****.***
```

Supplementary Figure 2. Amino acid sequence alignment of the parental protein entolimod (CBLB502), intermediate variants 33ML and 33MX, and the final next-generation TLR5 agonist GP532.

- Minimized 33ML variant (250 aa; obtained from CBLB502 via truncation of the ND0 domain, minimization of the flexible linker, and addition of a His_6 tag at the C-terminus linked via a thrombin cleavage site)
- Partially deimmunized 33MX variant [250 aa; obtained from 33ML via 17 aa substitutions including 15 Ala substitutions eliminating major neutralizing B-cell epitopes (shown in red font) and two Asn → Gln substitutions removing potential glycosylation sites (shown in green font)]
- GP532 (239 aa; obtained from 33MX via 4 additional aa substitutions (shown in blue font) eliminating two T cell epitopes and deletion of 11 C-terminal aa eliminating the third T cell epitope and the inflammasome-activating domain).

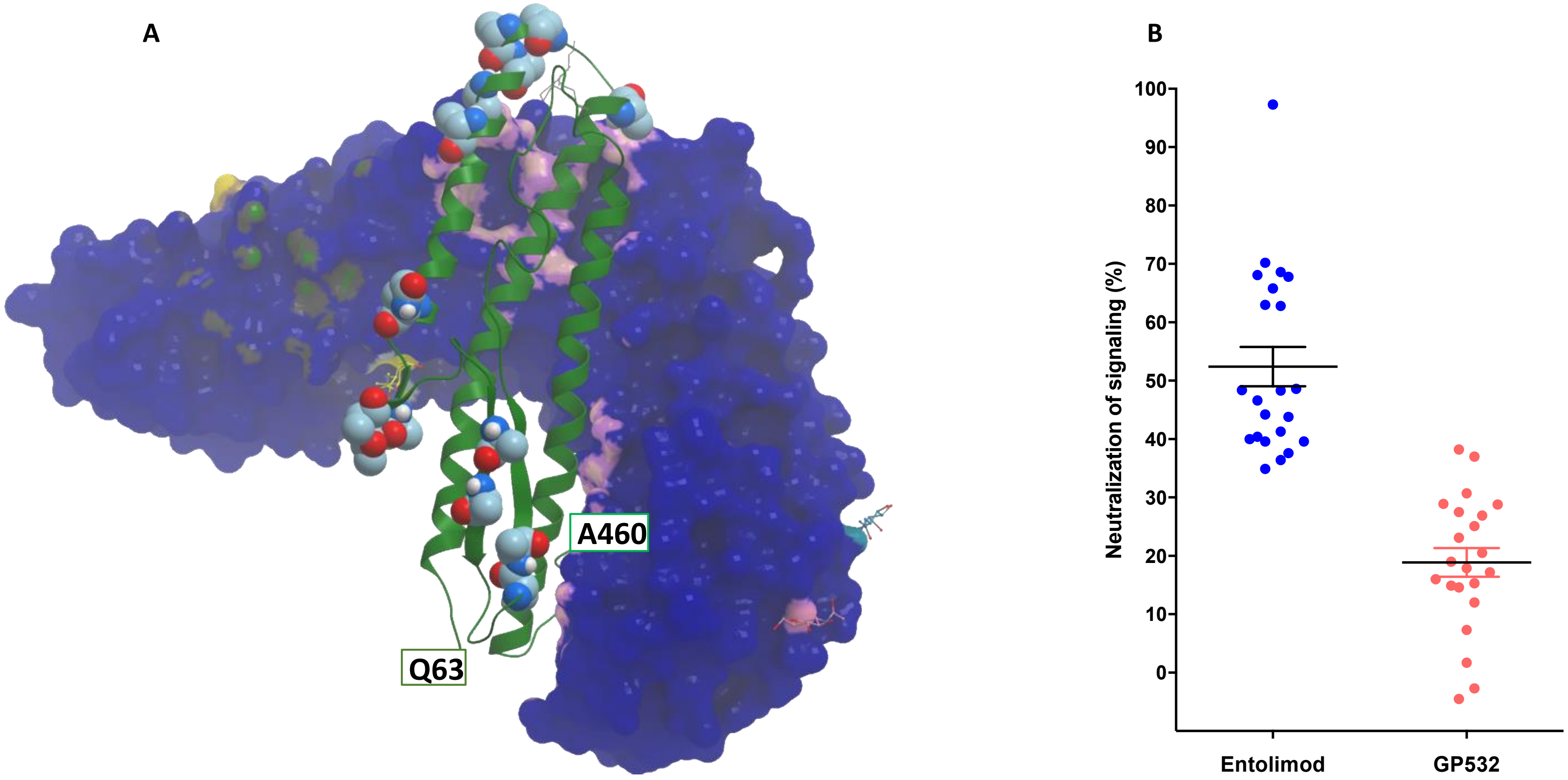

Supplementary Figure 3. Elimination of neutralizing B-cell epitopes in entolimod: 3D model of the human TLR5 ectodomain bound to the D1 domain of GP532 with introduced mutations (A) that led to significant suppression of inhibition of TLR5-NF-κB signaling activity by human neutralizing antibodies (B). A. In the displayed 1:1 heterodimer (one half of the 2:2 dimer of dimers), a segment of the TLR5 monomer is shown as a blue space-filling model with colored glycosylation sites. The helical D1 domain of entolimod (ND1 segment (Q63 - N175) + CD1 segment (T402 – A460)) is shown as a green ribbon diagram. Amino acid residues in the ND1 domain of GP532 resulting from site-directed mutagenesis (and modeled from the available 3D complex structure, PDB:3V47 (17), using ICM software) are shown as colored spheres. **B.** Confirmation of the resistance of GP532 to neutralizing antibodies in 22 selected human serum samples with high titers of entolimod-neutralizing antibodies (neutralization of entolimod >30%). The % reduction in activity of entolimod or GP532 in a cell-based NF-κB-lacZ reporter assay in the presence of individual human serum samples is shown with horizontal bars indicating the mean ±SD (n=22).

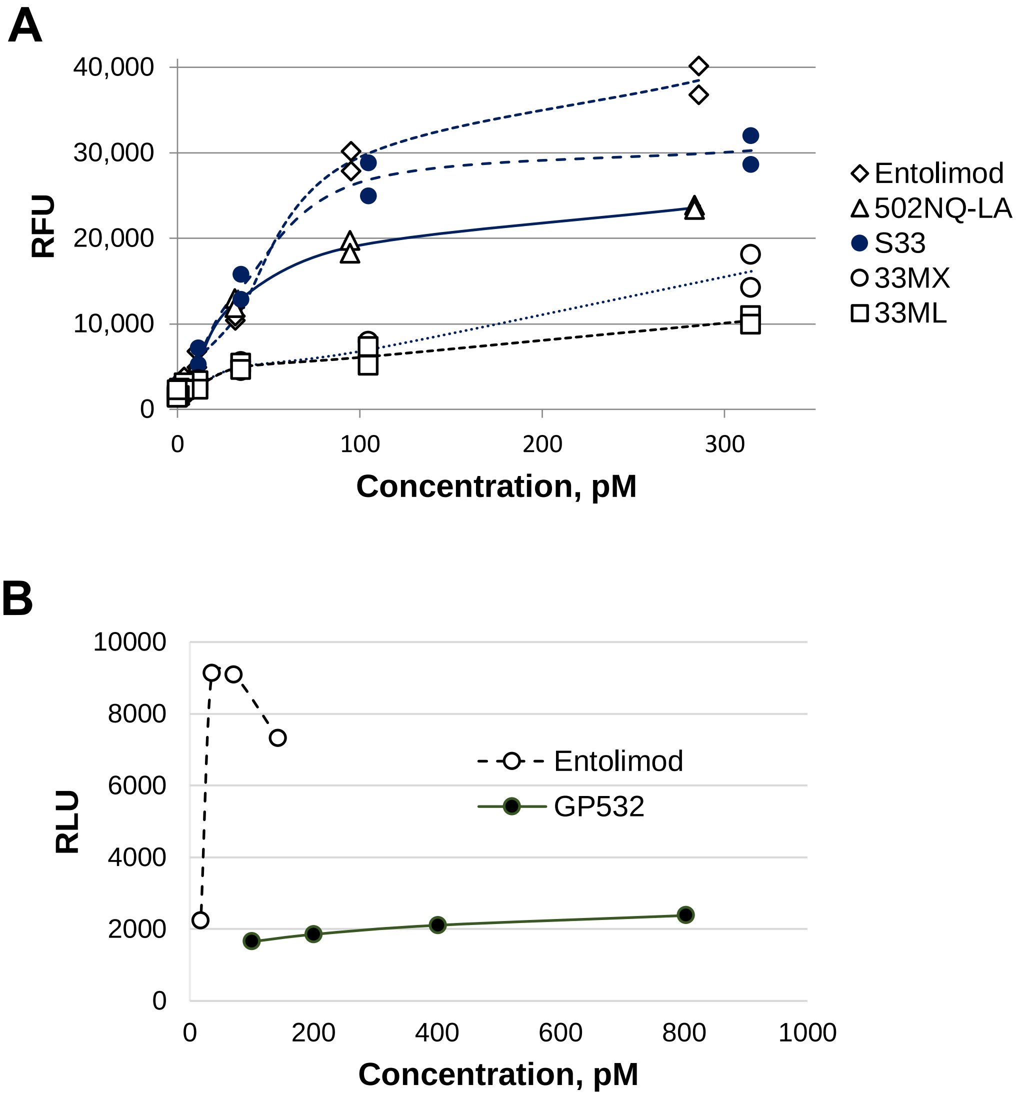

Supplementary Figure 4. Inflammasome activation by entolimod and protein variants with modifications at the C terminus. A. IL-1β-inducing activity of entolimod, 33ML- and 33MX-related protein variants (Supplementary Tables 2 and 3) assessed using THP1-NLRC4/HEK-Blue-KD-TLR5 cell co-cultures. Fluorescence (RFU) reflecting SEAP reporter enzyme activity was measured in supernatants collected from co-cultures after 20 hours incubation with the indicated proteins/concentrations. Markers indicate values for duplicate samples; trend lines pass through mean values. **B.** Inflammasome activation by entolimod and GP532 as indicated by caspase activation assay. THP1-NLRC4 cells were treated with the indicated concentrations of entolimod or GP532 for 5 hours. Caspase 1 activity was measured using Promega Caspase Glo-1-Inflammasome-assay and expressed as luminescence (RLU).

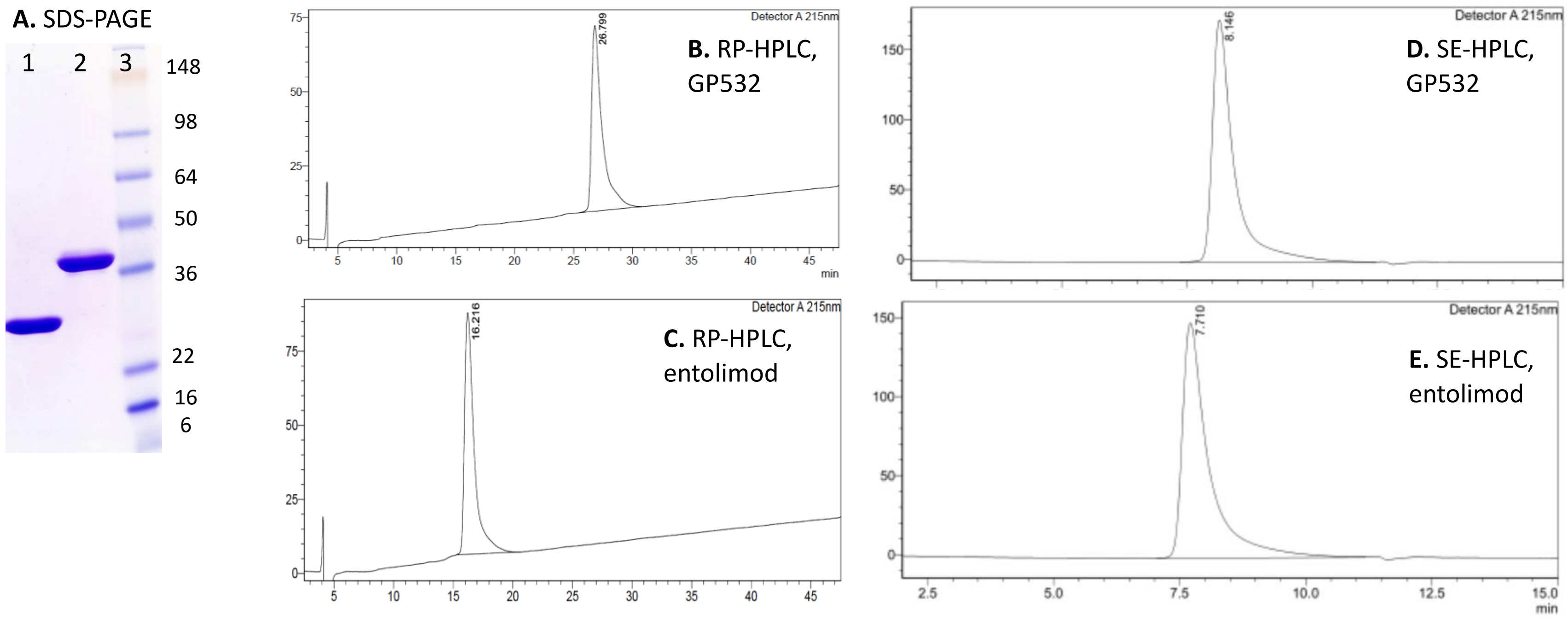

Supplementary Figure 5. GP532 expression and purification in comparison to GMP-manufactured entolimod drug product. The yield and extent of purification of GP532 and entolimod was assessed by SDS-PAGE (A; Lane 1 = GP532, Lane 2 = entolimod, Lane 3 = MW standards), RP-HPLC (B,C) and SE-HPLC (D,E).

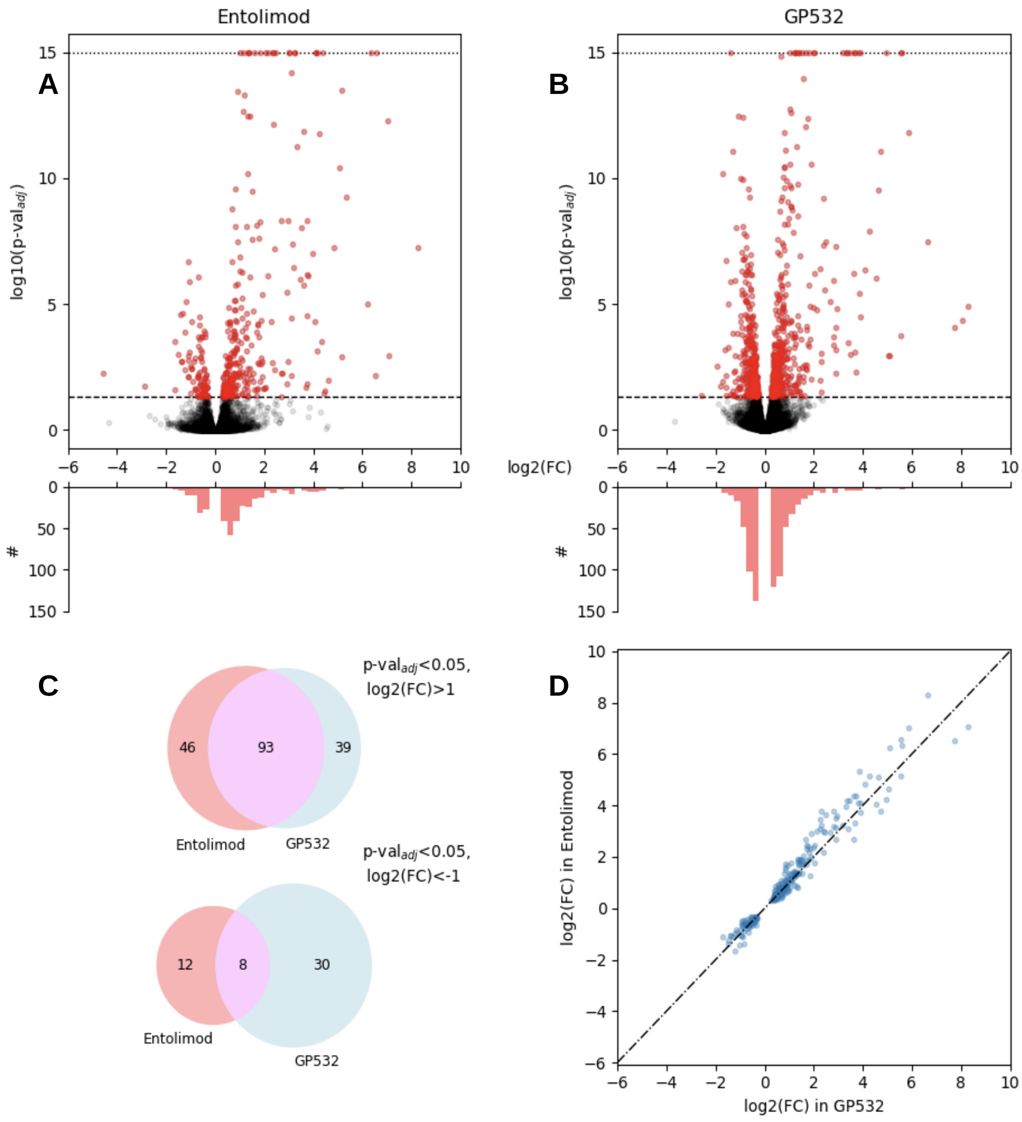

Supplementary Figure 6. Comparative analysis of differentially expressed (DE) genes in mouse livers 30 min after s.c. injection of GP532 or entolimod (1 µg/mouse) as compared to a vehicle-treated control group. A, B. Volcano plots of DE genes upon treatment with entolimod (A) or GP532 (B), with histograms for log2(FC) distribution shown below the x-axis. For volcano plots, dots representing DE genes with P-values below the threshold of statistical significance (0.05, shown by the dashed line) are shown in red, and all others are shown in black. All P-values below 1e-15 (shown by the dotted line) were set to this value for visualization purposes. **C.** Venn diagrams of DE genes that are significantly upregulated (log2(FC)>1, P-value<0.05; top diagram) or downregulated (log2(FC)<-1, P-value<0.05; bottom diagram) after entolimod (orange) or GP532 (blue) treatment. **D.** Correlation plot for log2(FC) for shared DE genes between GP532 and entolimod treatments.

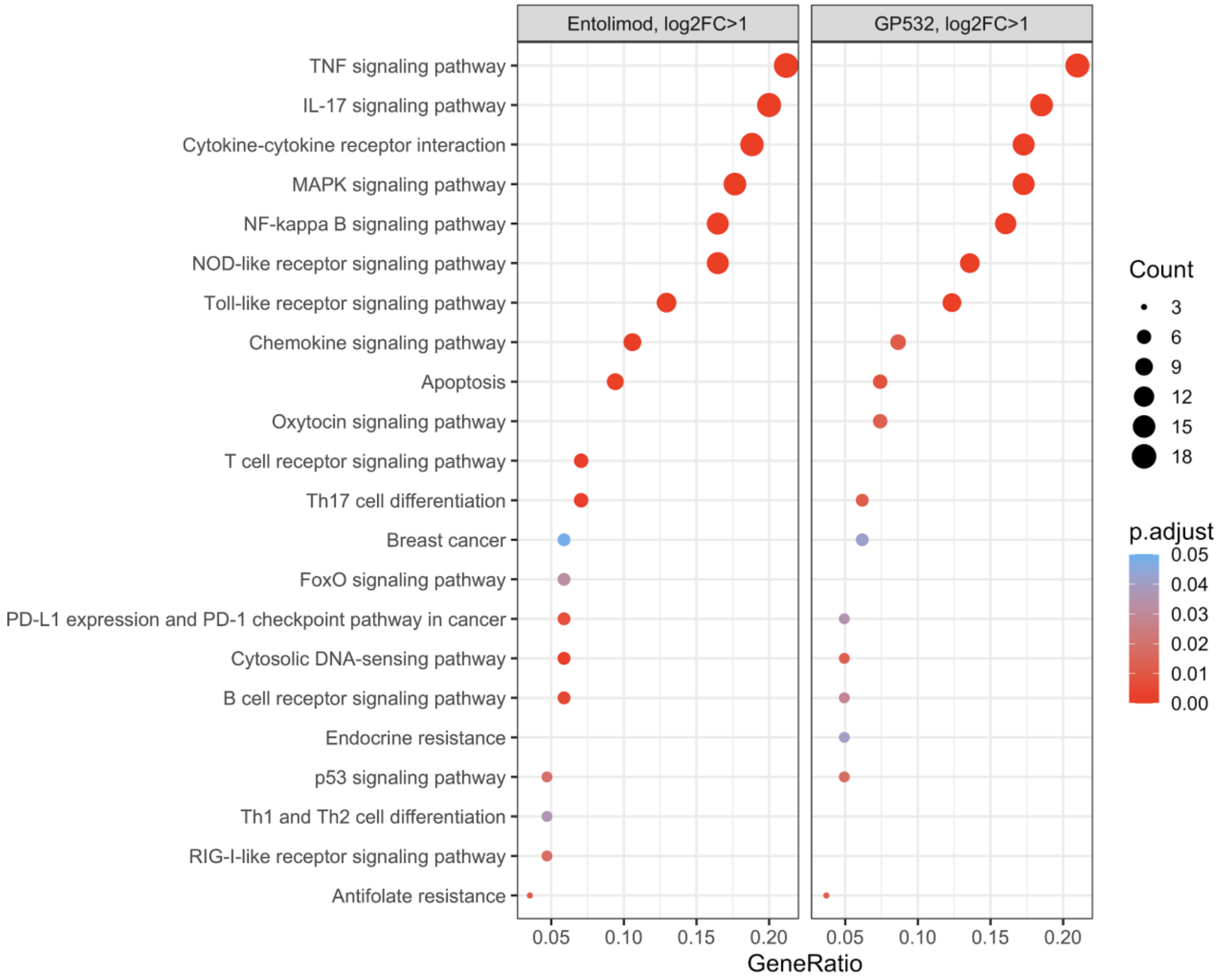

Supplementary Figure 7. KEGG pathway enrichment by differentially expressed genes identified in mouse liver at 30 min after treatment with GP532 or entolimod.

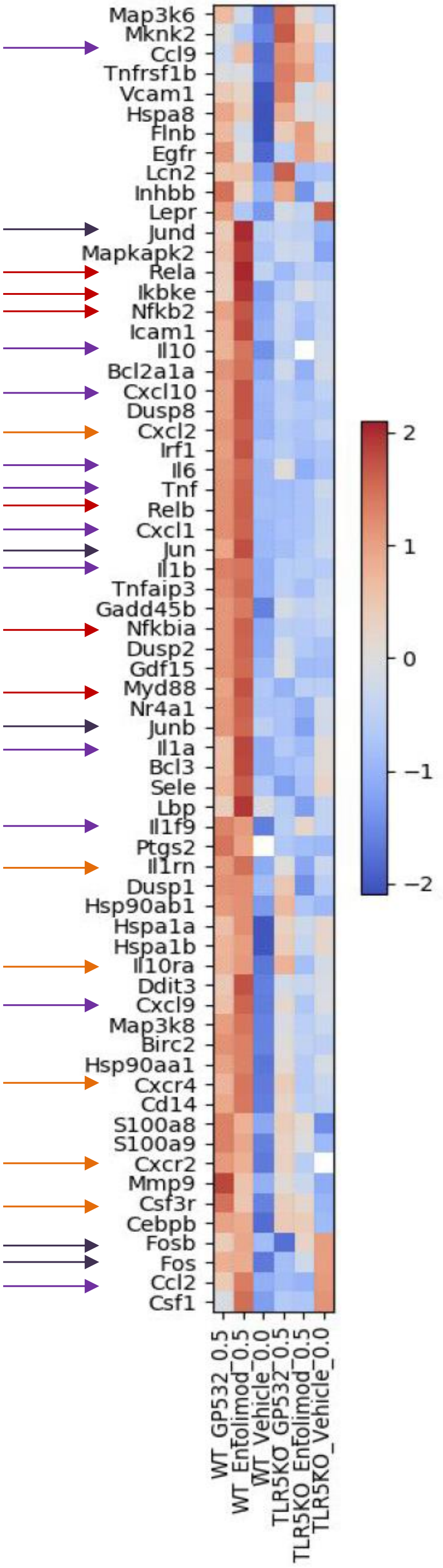

Supplementary Figure 8. Expanded heatmap of 93 differentially expressed genes representing the five top enriched pathways from RNAseq analysis of mouse livers 30 min after s.c. injection of GP532, entolimod or vehicle in C57BL/6 mice, wild type (lanes marked "WT") or TLR5-KO (lanes marked "TLR5KO"). Color-coded arrows indicate genes involved in known signaling/regulatory pathways (NF-κB or AP-1) as well as cytokines/chemokine ligands and receptors (see boxed legend below).

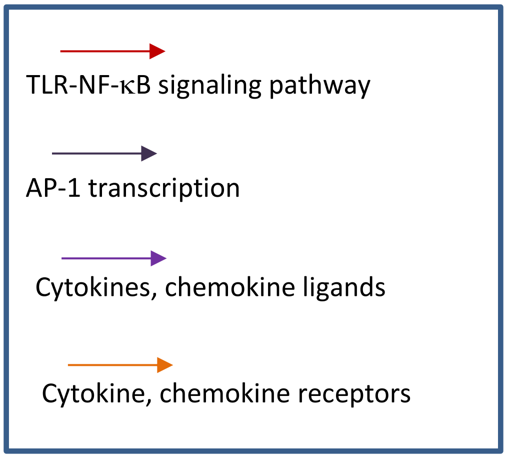

Genes were selected as follows: Genes from any of *Mus musculus* KEGG Pathways mmu04668 (TNF signaling pathway), mmu04657 (IL-17 signaling pathway), mmu04060 (Cytokine-cytokine receptor interaction), mmu04010 (MAPK signaling pathway), mmu04064 (NF-κB signaling pathway), which were significantly upregulated (deDeq2 log2FC > 0, adjusted P-value < 0.05) in WT mice 30 min after treatment with either entolimod or GP532 compared to vehicle-treated WT controls. Data was analyzed using deSeq2 R package. For better visualization, deseq2 normalized counts were log2-transformed and z-score values were calculated independently for each gene (exact zero values were excluded from calculation and are shown as white squared in the image). Genes were sorted using unweighted pair group method with arithmetic mean (UPGMA) hierarchical clustering algorithm. The red-blue heatmap reflects z-scores ranging from 2 to -2 as shown by the narrow vertical bar on the right side of the figure.

The 93 genes shown here include the 46 genes from Figure 9 as well as other significantly DE genes, including many encoding cytokines or chemokines (*Cxcl2, Cxcl10, Cxcl1, Tnf, Il1a, Il1b, Il6, l10. Il1f9*) or cytokine/chemokine receptors (*Il15ra, Il1rn, Csf3r, Eda2r*). It should be noted that transcriptional activation of production of certain cytokines, such as IL-1β, may enrich the procytokine pool inside cells but not lead to secretion of processed mature cytokines without inflammasome activation (which is blocked in the case of GP532).

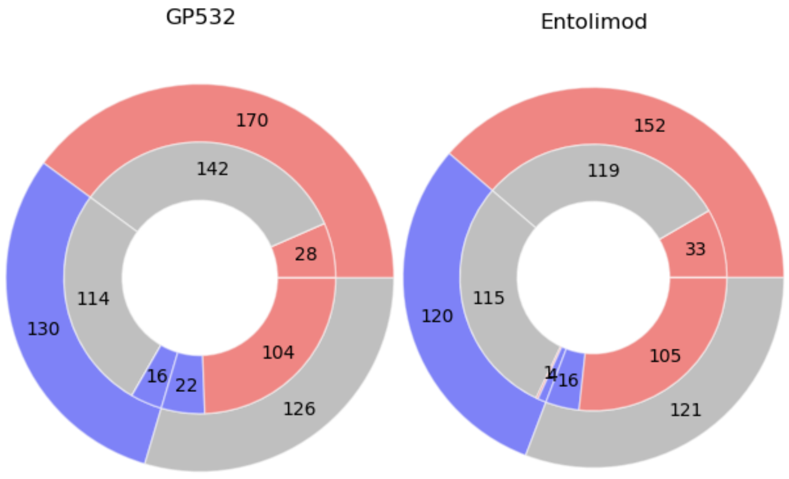

Supplementary Figure 9. Counts of Differentially Expressed (DE) genes in mouse livers collected at 30 min (inner circle) or 24 hrs (outer circle) after s.c. injection of GP532 (left) or entolimod (right). Colors correspond to subsets of DE genes that are significantly upregulated (log2FC > 1; red) or downregulated (log2FC < -1; blue) compared to a vehicle-treated control group. Gene expression in liver samples collected at 30 min vs 24 hrs after TLR5 agonist administration showed only a relatively small degree of overlap in DE genes at the two timepoints. Most prominently, most of the genes found to be upregulated by entolimod or GP532 at 30 min were no longer upregulated at 24 hrs.

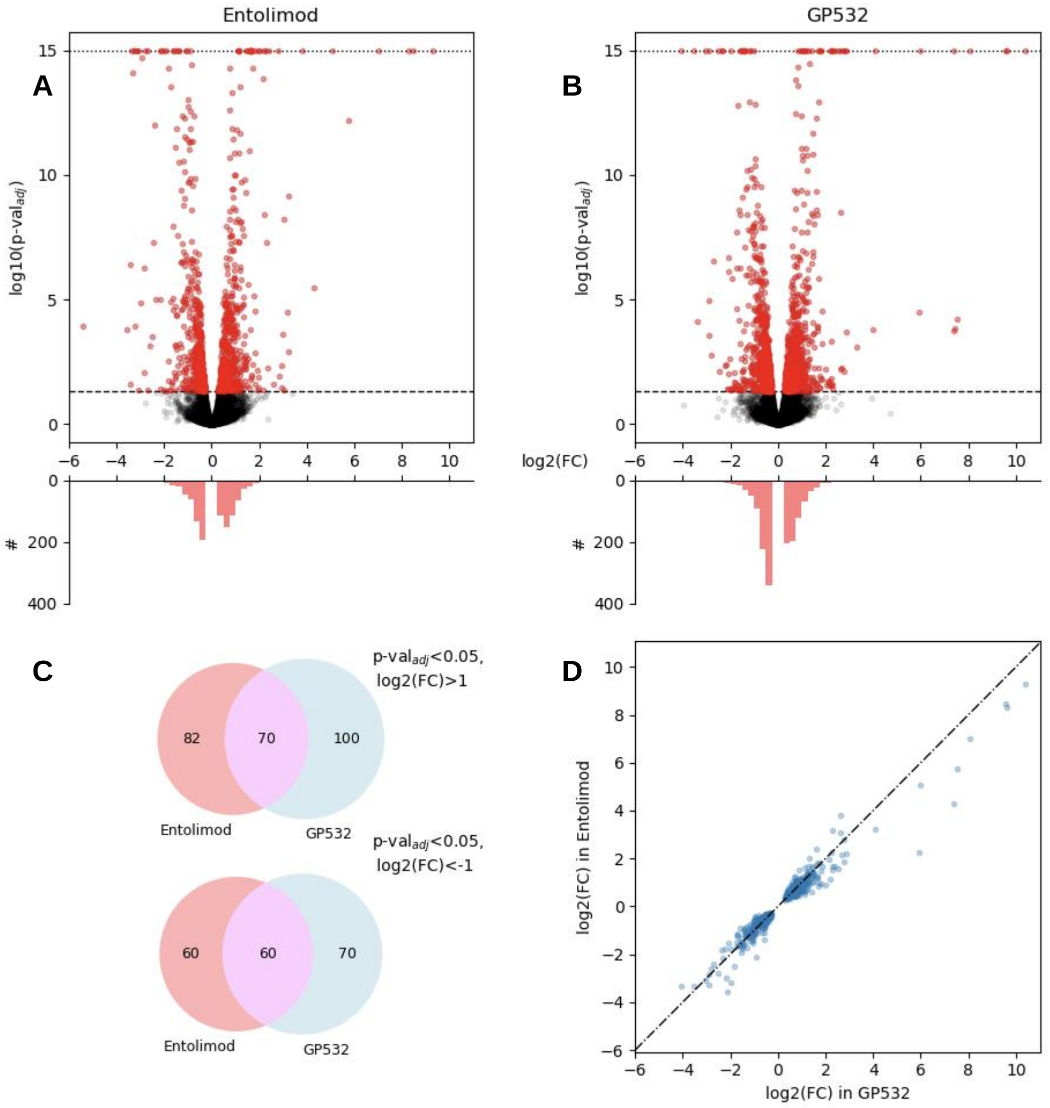

Supplementary Figure 10. Comparative analysis of differentially expressed (DE) genes in mouse livers 24 hrs after s.c. injection of GP532 or entolimod (1 μg/mouse) as compared to a vehicle-treated control group. A-B. Volcano plots of DE genes upon treatment with entolimod (A) or GP532 (B), with histograms for log2(FC) distribution shown below the x-axis. For volcano plots, dots representing DE genes with P-values below the threshold of statistical significance (0.05, shown by the dashed line) are shown in red, and all others are shown in black. All P-values below 1e-15 (shown by the dotted line) were set to this value for visualization purposes. **C.** Venn diagrams of DE genes that are significantly upregulated (log2(FC)>1, P-value<0.05; top diagram) or downregulated (log2(FC)<-1, P-value<0.05; bottom diagram) after entolimod (orange) or GP532 (blue) treatment. **D.** Correlation plot for log2(FC) for DE genes shared between GP532 and entolimod treatments.

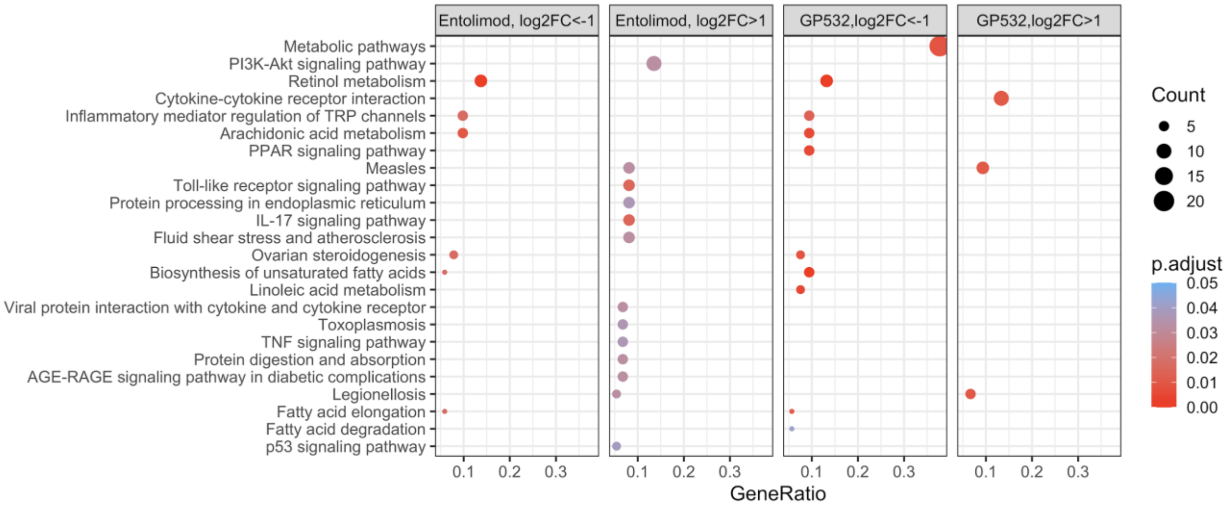

Supplementary Figure 11. KEGG pathway enrichment by differentially expressed (DE) genes identified in mouse livers at 24 hrs after treatment with GP532 or entolimod.

Pathway enrichment analysis of DE genes shared between entolimod and GP532 at the 24 hr time point (60 upregulated and 70 downregulated) revealed relatively low levels of enrichment over a very different set of pathways vs. those affected at 30 minutes.

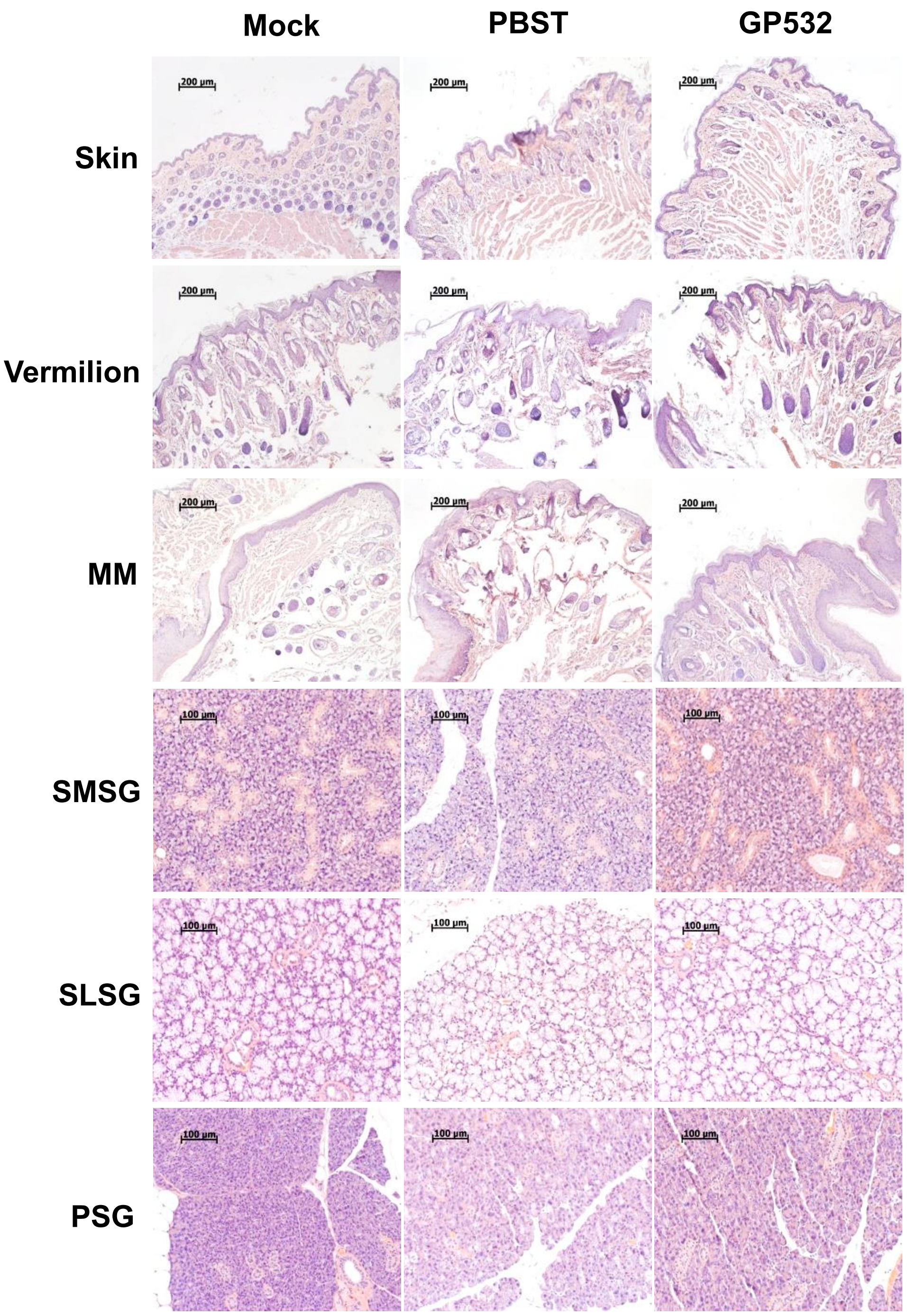

Supplementary Figure 12. GP532 protects mouse mucosal tissues and salivary glands against damage induced by localized H&N radiotherapy. Female FVB/NJ mice (n = 15) were administered s.c. 0.3 µg GP532 30 min prior to receiving 15 Gy irradiation (X-ray) to the H&N region. Control groups of mice were injected with PBST (vehicle) prior to irradiation or were not injected or irradiated ("mock"). Thirty days post-irradiation, mice were euthanized and mucosal tissues and salivary glands were collected. Representative hematoxylin and eosin-stained sections are presented. MM, mouth mucosa; LN, lymph nodes; SMSG, submandibular salivary glands; SLSG, sublingual salivary glands; PSG, parotid salivary glands. Scale bars represent 200 µm for skin, vermilion and MM and 100 µm for SMSG, SLSG and PSG.

SUPPLEMENTARY TABLES

Supplementary Table 1. Human immune response to entolimod (CBLB502) administration in three clinical trials

Dose range/Regimen/ Number of participants	Time point	Number of subjects with neutralizing entolimod-reactive Ab	% of total number of subjects
Study 1: An open-label clinical trial of the safety and pharmacokinetics of single, escalating doses of CBLB502 in healthy volunteers			
2-50 µg per injection/Single i.m. injection/50 (healthy volunteers)	Predose	5	10%
	Day 12	49	98%
	Day 28	47	94%
Study 2: A randomized, open-label, Phase 2 study of CBLB502 in healthy subjects			
25-35 µg per injection/Single or double (day 1 and 3) i.m. injection/100 (healthy volunteers)	Predose	8	8%
	Day 9	54	54%
	Day 30	97	97%
Study 3: A phase 1 study to evaluate the safety and pharmacokinetic profile of CBLB502 in patients with advanced cancers (https://clinicaltrials.gov/ct2/show/NCT01527136?term=Cleveland+Biolabs&cntry=US&draw=2&rank=2).			
5-20 µg per injection/once daily s.c. on days 1-5 /13 (cancer patients)	Predose	0[1]	0
	Day 5	0	0
	Day 15	12	92%
	Day 29	13	100%
30 or 40 µg per injection/i.m. on day 1 and s.c. on day 4, 8 and 11/12 (cancer patients)	Predose	0[1]	-
	Day 4	0	0%
	Day 8	5[2]	71%[2]
	Day 11	7[2]	100%[2]

[1] Pre-screened and selected for the absence of neutralizing entolimod-reactive Abs
[2] Only 7 patients out of 12 received entolimod on Days 8 and 11

Supplementary Table 2. Mapping of the minimal functional core of entolimod

Supplementary Table S2. Mapping of the minimal functional core of entolimod													
**Variant ID**	**Brief description**	**STRUCTURAL COMPOSITION***									**Size (aa)**	**NfkB signaling EC50/EC50E#**
		**N-tag**	**ND0**	**NS**	**ND1**	**FL**	**CD1**	**CS**	**CD0**	**C-tag**		
**Entolimod**	parental full-size protein	- 33 aa	1 - 33	34 - 45	46 - 175	16 aa	402 - 464	465 - 470	471 - 504	NA	**329**	**=1**
SY3	ΔND0 & ΔCD0	- 33 aa	deleted	34 - 45	46 - 175	16 aa	402 - 464	465 - 466	deleted	NA	**262**	300
**S33**	ΔND0	- 33 aa	deleted	34 - 45	46 - 175	16 aa	402 - 464	465 - 470	471 - 504	NA	**300**	**1**
33CT	ΔND0 & C-tag	deleted	deleted	34 - 45	46 - 175	16 aa	402 - 464	465 - 470	471 - 504	+ 12 aa	**275**	2
37CT	ΔND0 & partial ΔNS & C-tag	deleted	deleted	37 - 45	46 - 175	16 aa	402 - 464	465 - 470	471 - 504	+ 12 aa	**275**	3
N45	ΔND0 & ΔNS	- 33 aa	deleted	deleted	46 - 175	16 aa	402 - 464	465 - 470	471 - 504	NA	**288**	4
45CT	ΔND0 & ΔNS & C-tag	deleted	deleted	deleted	46 - 175	16 aa	402 - 464	465 - 470	471 - 504	+ 12 aa	**285**	4
v467	ΔCD0	- 33 aa	1 - 33	34 - 45	46 - 175	16 aa	402 - 464	465 - 466	deleted	NA	**295**	50
v461	ΔCD0 & truncation in CD1	- 33 aa	1 - 33	34 - 45	46 - 175	16 aa	402 - 460	deleted	deleted	NA	**289**	100
v445	ΔCD0 & truncation in CD1	- 33 aa	1 - 33	34 - 45	46 - 175	16 aa	402 - 443	deleted	deleted	NA	**272**	700
470CT	ΔCD0 & C-tag	deleted	1 - 33	34 - 45	46 - 175	16 aa	402 - 464	465 - 470	deleted	+ 12 aa	**274**	40
v33-485	partial ΔCD0	- 33 aa	deleted	34 - 45	46 - 175	16 aa	402 - 464	465 - 470	471 - 485	NA	**281**	10
**33ML**	ΔND0 & partial Δ(FL_CD1) & C-tag	deleted	deleted	34 - 45	46 - 175	3 aa	413 - 464	465 - 470	471 - 504	+ 12 aa	**250**	**1**

*See Figures 2 and S1 for domain definitions, abbreviations, and sequences; changes in the structural composition of protein variants relative to entolimod are shown in red font.

Bioactivity of the variant (EC50) normalized to that of entolimod (EC50E) in a cell-based NF-κB-lacZ reporter assay

Supplementary Table 3. Stage 2 engineering: mapping and elimination of neutralizing B-cell epitopes. Effects of amino acid substitutions on resistance of the corresponding protein variant to inhibition by human entolimod-reactive neutralizing antisera (P12 and P14) in a cell-based NF-κB-lacZ reporter assay. *See Supplementary Table 4 for data on a broader range of protein variants and antibodies.*

**Protein variant**	**EC50/ $EC50_E$**[1]	**Amino acid residue** (numbered as in *Salmonella* flagellin; substitution with alanine indicated by black box)															**$IC50/IC50_E$**[2]	
		**Asn 68**	**Asn 100**	**Thr 102**	**Ser 104**	**Ser 106**	**Asp 107**	**Ser 110**	**Asp 113**	**Phe 131**	**Asn 132**	**Gly 133**	**Gln 142**	**Lys 144**	**Glu 153**	**Thr 154**	**P12**	**P14**
**33ML**	1.3																~1	~1
ME100	0.6		Ala	Ala													~1	~2
ME104	1.0				Ala	Ala	Ala										~1	~2
ME110	0.7							Ala	Ala								~1	~2
ME132	0.9										Ala	Ala					~2	~3
ME142	0.3												Ala	Ala			~2	~2
MIXN	1.5	**Ala**								Ala			Ala		Ala	Ala	~5	~5
**33MX**	1.5	**Ala**	Ala	Ala	Ala	Ala	Ala	Ala	Ala	Ala	Ala	Ala	Ala	Ala	Ala	Ala	~6	~7

[1]Bioactivity of each protein variant (EC50 in cell-based NF-κB-lacZ reporter assay) normalized to that of entolimod ($EC50_E$)

[2]Inhibitory effect of neutralizing antisera on the bioactivity of protein variants in a cell-based NF-κB-lacZ reporter assay (IC50 of the variant normalized to that of entolimod, $IC50_E$). Human serum samples from two patients (P12, P14) who developed the strongest neutralizing antibody response after entolimod administration in a clinical trial were tested.

Supplementary Table 4. Alanine scanning mutagenesis of neutralizing B-cell epitopes in entolimod and characteristics of intermediate protein variants

Variant ID	Scaffold	AA substitutions	Size (aa)	NfkB signaling EC50/EC50E*	Neutralization by antibodies (IC50/IC50E**)					
					mAB4D11	mAb11D04	NSP61	NSP103	Human P12 antisera	Human P14 antisera
**MIM1**	Entolimod	N455A; N457A	329	**0.9**	No improvement in neutralization with any of the tested antibodies					
**MIM2**	Entolimod	N455A; N457A; R460A	329	**1.2**						
**MIM3**	Entolimod	N448A; N451A; N455A, N457A; R460A	329	**4.6**						
**MIM4**	Entolimod	Q439::F442 deletion; N448A; N451A; N455A, N457A; R460A	329	**37.0**						
**MIM5**	Entolimod	Q439A; N440K; R441A; N448A; N451A; N455A, N457A; R460A	329	**54.0**						
**ME42**	33ML	D42A; A45G	250	**1.3**	4x	5x	2x	2x	1x	1x
**ME100**	33ML	N100A; T102A	250	**0.6**	>5x	>10x	3x	2x	1x	2x
**ME104**	33ML	S104A; S106A; D107A	250	**1.0**	>5x	>10x	>5x	>5x	1x	2x
**ME110**	33ML	S110A; D113A	250	**0.7**	>5x	>10x	3x	2x	1x	2x
**ME117**	33ML	Q117A; E120A	250	**0.8**	5x	>10x	2x	2x	1x	1x
**ME124**	33ML	R124A; N127A; Q128A	250	**0.9**	3x	6x	2x	1x	3x	1x
**ME132**	33ML	N132A; G133A	250	**0.9**	>5x	>10x	4x	>5x	2x	3x
**ME142**	33ML	Q142A; K144A	250	**0.3**	>5x	>10x	3x	>5x	2x	2x
**ME150**	33ML	N150S; D151A; G152A	250	**0.5**	>5x	>10x	3x	2x	1x	2x
**ME468**	33ML	Y468A; A469G; T470A; S473A	250	**0.7**	>5x	>10x	2x	2x	4x	1x
**ME104 N**	33ML	N100A; T102A; S104A; S106A; D107A; S110A; D113A	250	**0.9**	1x	2x	2x	2x	1x	1x
**MIXN**	33ML	N68A; F131A; Q142A; E153A; T154A	250	**1.5**	~10x	~10x	ND	ND	4-5X	4-5X
**MIXC**	33ML	N440A; D443A; S444A; T447A	250	**1.5**	~10x	~10x	ND	ND	no improvement	
**33MX**	33ML	N68A; F131A; Q142A; E153; T154A; S104A; S106A; D107A; S110A; D113A; N132A; G133A; K144A; N127Q; N474Q	250	**1.3**	>4x	>4x	ND	ND	6x	7x

*Bioactivity of the variant (EC50) normalized to that of entolimod in a cell-based NF-κB-lacZ reporter assay

**Inhibition of NF-κB signaling in NF-κB-lacZ reporter cells by neutralizing antibodies (mouse monoclonal antibodies mAB4D11 and mAb11D04, normal human serum samples from entolimod-naïve anonymous donors NSP61 and NSP103, and human serum samples from patients P12 and P14 injected with entolimod in clinical trials). IC50 normalized to that of entolimod; ND, not determined.

Supplementary Table 5. Elimination of major T-cell epitopes in entolimod

	Variant ID	Mutations	Position	EC50^, ng/ml	EC50(variant)/ EC50(33MX)*
1	TEM1-49A	I49A	Epitope 1	0.113	1.4
2	TEM1-49E	I49E	Epitope 1	0.147	1.6
3	TEM1-49T	I49T	Epitope 1	0.137	1.5
4	TEM1-53A	F53A	Epitope 1	0.112	1.4
5	TEM1-54D	T54D	Epitope 1	0.146	1.7
6	TEM1-58E	K58E	Epitope 1	0.181	2
7	TEM1-AD	F53A; T54D	Epitope 1	0.089	2.8
8	TEM1-SD	F53S; T54D	Epitope 1	0.117	2.8
9	TEM1-TD	F53T; T54D	Epitope 1	0.148	3.2
10	TEM1-DD	T54D; S55D	Epitope 1	0.171	4
11	TEM2-480A	I480A	Epitope 2	0.662	7.5
12	TEM2-481D	L481D	Epitope 2	>10	ND
13	TEM2-481H	L481H	Epitope 2	>10	ND
14	TEM2-482D	Q482D	Epitope 2	0.441	5.8
15	TEM2-486D	T486D	Epitope 2	0.479	6.3
16	TEM2-481A	L481A	Epitope 2	0.836	9.7
.17	TEM2-488T	V488T	Epitope 2	0.103	1.2
18	TEM2-K1D	I480K; L481D	Epitope 2	>10	ND
19	TEM2-D6D	Q482D; T486D	Epitope 2	>2.5	ND
20	TEM2492DG	T486D; A492G	Epitope 2	>2.5	ND
21	TEM2-ADT	I480A; L481D; V488T	Epitope 2	>10	ND
22	TEM2-ADD	I480A; L481D; V489D	Epitope 2	>10	ND
23	TEM2-ANT	I480A; L481N; V488T	Epitope 2	>10	ND
24	TEM2-AND	I480A; L481N; V489D	Epitope 2	>10	ND
25	TEM2-AST	I480A; L481S; V488T	Epitope 2	>10	ND
26	TEM2-ASD	I480A; L481S; V489D	Epitope 2	>10	ND
27	GP532	I49A; F53A; Q482D; V488T; *Δ*(A491-L501)	Epitopes 1 and 2; Epitope 3 deleted	0.188	2.9

^ Activity in cell-based NF-κB-lacZ reporter assay; * EC50 for variant normalized to that of 33MX; "ND" – not determined

Notes: Following identification of three predicted T-cell epitopes, we generated a large set of 33MX-based protein variants containing single or double/triple mutations (to increase confidence in epitope elimination) in the identified epitopes as suggested by the iTope program (see Supplementary Methods, Abzena Report

2) and tested them for activity in NF-κB-lacZ reporter cells. Notably, while most single and double mutants in the area of Epitope 1 demonstrated preserved signaling activity (met the predefined threshold of EC50 ≤ 5 pM), many single and especially double and triple mutants in the area of Epitope 2 showed a much greater loss of activity. Since the part of the molecule containing Epitope 2 (domain CD0) is not present in the available 3D model of entolimod, searching for acceptable mutations in this area was only possible by an iterative trial and error process. Ultimately, based on their minor effect on NF-κB signaling, we selected four individual mutations (two each in Epitopes 1 and 2) for the final design of the next-generation drug candidate GP532. In addition to these four mutations, GP532 has a deletion of the C-terminal 11 amino acids of flagellin (Ala492 – Leu502, also present in entolimod and 33MX; see Figure 2 and Supplementary Figure 1), which effectively eliminates Epitope 3 (along with inflammasome activation). Combination of all of these modifications in GP532 led to only a moderate ~ 3-4-fold increase in EC50 for *in vitro* NF-κB signaling as compared to 33MX, which was even less than the effect of one of the Epitope 2 single mutations (see Supplementary Table 6).

Supplementary Table 6. Stage 3 engineering: mapping and elimination of T-cell epitopes. Modifications made to eliminate mapped T-cell epitopes via site-directed mutagenesis (Epitopes 1 and 2) and C-terminal truncation (Epitope 3) are shown in black boxes.

**Protein variant**	**Size (aa)**	**Epitope 1**	**Epitope 2**	**Epitope 3**	**EC50[1](ng/ml)**	**$EC50/EC50_{33MX}$[2]**
**33MX**	250				0.08	=1
TEM1-49A	250	I49A			0.113	1.4
TEM1-53A	250	F53A			0.112	1.4
TEM2-482D	250		Q482D		0.441	5.8
TEM2-488T	250		V488T		0.103	1.2
**GP532**	239	I49A; F53A	Q482D; V488T	*Δ*(A491-L501)	0.188	2.9

[1]Bioactivity of each protein variant in cell-based NF-κB-lacZ reporter assay, expressed as EC50

[2]EC50 of each protein variant normalized to that of 33MX

Supplementary Table 7. Comparison of the immunogenicity of GP532 and entolimod across a panel of 50 human PBMC samples from healthy donors^

Donors 1-25	Readout #1: Proliferation (SI ≥1.90, p <0.05)			Readout #2: ELISpot (IL-2) (SI ≥1.90, p <0.05)			Donors 26 - 50	Readout #1: Proliferation (SI ≥1.90, p <0.05)			Readout #2: ELISpot (IL-2) (SI ≥1.90, p <0.05)		
	GP532	Entolimod	KLH control	GP532	Entolimod	KLH control		GP532	Entolimod	KLH control	GP532	Entolimod	KLH control
**Donor 1**							**Donor 26**						
**Donor 2**							**Donor 27**						
**Donor 3**							**Donor 28**						
**Donor 4**							**Donor 29**						
**Donor 5**							**Donor 30**						
**Donor 6**							**Donor 31**						
**Donor 7**							**Donor 32**						
**Donor 8**							**Donor 33**						
**Donor 9**							**Donor 34**						
**Donor 10**							**Donor 35**						
**Donor 11**							**Donor 36**						
**Donor 12**							**Donor 37**						
**Donor 13**							**Donor 38**						
**Donor 14**							**Donor 39**						
**Donor 15**							**Donor 40**						
**Donor 16**							**Donor 41**						
**Donor 17**							**Donor 42**						
**Donor 18**							**Donor 43**						
**Donor 19**							**Donor 44**						
**Donor 20**							**Donor 45**						
**Donor 21**							**Donor 46**						
**Donor 22**							**Donor 47**						
**Donor 23**							**Donor 48**						
**Donor 24**							**Donor 49**						
**Donor 25**							**Donor 50**						

Statistics	GP532	Entolimod	KLH control
**Readout 1**	**8%**	**28%**	**52%**
**Readout 2**	**10%**	**20%**	**60%**
**Readout 1 or 2**	**18%**	**40%**	**72%**
**Readout 1 & 2**	**0%**	**8%**	**40%**
**Correlation of 1 and 2***	**0%**	**29%**	**74%**

^ Comparison was performed over the representative panel of PBMCs from 50 healthy donors using two readouts: readout #1 - proliferation assay by incorporation of [^{3}H]-thymidine and readout #2 - ELIspot assay for IL-2 measurement. The highly immunogenic KLH antigen was used as a positive control for both readouts. *Correlation is expressed as the percentage of proliferation responses that were also positive in the IL-2 ELISpot assay.

Supplementary Table 8. NF-κB activity induced by selected protein variants with C-terminal deletions and mutations

Variant ID	Scaffold	Size, aa	NF-κB signaling^		Description
			EC50, pM	EC50/ EC50E*	
502NQ-LA	entolimod	329	0.64	0.7	L500A; L502A; L503A (+ N-Q replacements)
470CT	entolimod	274	32.5	36.1	Δ(Thr470-Leu502) + C-terminal tag
MF227C	33ML	227	5.6	6.2	Δ(Thr470-Leu502) + C-terminal tag
33MX-LA	33MX	250	8.1	9.0	L500A; L502A; L503A
491MX	33MX	239	1.9	2.1	Δ(Ala492-Leu502) + C-terminal tag

^ Activity in cell-based NF-κB-lacZ reporter assay

* EC50 for variant normalized to that of entolimod

"N-Q replacement" indicates elimination of glycosylation sites

"C-terminal tag" indicates placement of a His6-tag at the C-terminus instead of the N-terminus (as in entolimod)

Supplementary Methods. Abzena Report 1.
EpiScreen T Cell Epitope Mapping in Synthetic Peptide Library Representing the Amino Acid Sequence of Bacterial Flagellin Derivative.

An Abzena company
ANTITOPE*
Enabling better biopharmaceuticals

*** ANTITOPE is a former name of ABZENA (https://abzena.com)**

Report: CBL01 Cleveland BioLabs, Inc.

EpiScreen™ T Cell Epitope Mapping

26th October 2015

Antitope Ltd.
Babraham Research Campus
Babraham
Cambridge
CB22 3AT
UK

Cleveland BioLabs, Inc.
F.A.O: Vadim Mett, PhD
73 High St.
Buffalo
NY 14203
USA

Signatures

Author(s):

Name	Signature	Date
E.A. Cloake	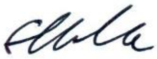	26th October 2015
M.H. Fogg	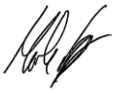	26th October 2015

Additional Contributors:

T. Jones
H. Reynolds
K. Welch
A. Rust
T. Fisher
B. Campbell
F. Grant
B. Cambi
G. Burns
F. Spalding
D. Geere
D. Pazeraitis
J. Cruickshank

Table of Contents

Table of Contents 3
List of Abbreviations 4
List of Tables 5
List of Figures 6
1. Summary 7
2. Introduction 8
3. Methods 9
3.1 EpiScreen™ Donor Selection 9
3.2 EpiScreen™Analysis: Proliferation Assay 10
3.3 EpiScreen™ Data Analysis 10
3.4 Endotoxin testing of peptides 11
3.5 *In Silico* Analysis of Peptides 11
4. Results and Discussion 12
4.1 Epitope 1 14
4.2 Epitope 2 14
4.3 Epitope 3 15
5. Conclusion 20
6. References 21
7. Appendix I 23
8. Appendix II 27

List of Abbreviations

Abbreviation	*Description*
BSA	Bovine serum albumin
CPM	Counts per minute
CV	Coefficient of variance
DMSO	Dimethyl sulphoxide
EBV	Epstein Barr Virus
EBNA	Epstein Barr virus nuclear antigen
HA	Haemagglutinin
HLA	Human leukocyte antigen
HTA	Human Tissue Authority
IFV	Influenza virus
KLH	Keyhole limpet haemocyanin
LAL	Limulus amoebocyte lysate
MHC	Major histocompatibility complex
PBMC	Peripheral blood mononuclear cells
PBS	Phosphate buffered saline
PHA	Phytohaemagglutinin
SD	Standard deviation
SI	Stimulation index
SSO	Sequence specific oligonucleotide

List of Tables

Table 1. Summary of individual donor responses to 80 peptides. Positive responses (SI ≥2.00, *p* <0.05, including borderline responses SI ≥1.90) are indicated by the donor number and individual SI are shown in brackets next to the corresponding donor. The background response rate was 4.3% in the non-adjusted data and 4.9% in the adjusted data. Peptides inducing positive T cell proliferation above this frequency (positive response in ≥3 donors) contained T cell epitopes (indicated in yellow). Potential p1 anchors are highlighted in red (predicted by iTope™). 19

Table 2. Summary of magnitude (mean SI and standard deviation) and frequency (% donor response) of positive T cell proliferation responses. Potential P1 anchor residues are shown in red (predicted by iTope™). 19

List of Figures

Figure 1. Comparison of the frequency of donor allotypes expressed in the CBL01 study cohort (n=50) with the European/North American and world population. 9

Figure 2. $CD4^+$ T cell epitope map using peptides tested against PBMC from 50 healthy donors. The non-adjusted and adjusted proliferation assay data for the 80 test peptides and controls C3, C32 and KLH. Peptides inducing positive (SI ≥2.00, *p* <0.05) T cell proliferation responses at a frequency above the background response threshold (indicated by the red dotted line) contain T cell epitopes. KLH induced positive responses (SI ≥2.00, *p* <0.05) in 92% of donors in both the non-adjusted and adjusted data sets. 13

Figure 3. Potential core 9mer HLA-DR restricted epitope within peptide 6. Using iTope™ two possible core 9mer binding registers (in blue with P1 anchors highlighted red) were identified within the peptide. The p1 and p9 anchor residues are indicated in the figure. 14

Figure 4. Potential core 9mer HLA-DR restricted epitope within peptides 71, 72, 73 and 74. Using iTope™ three possible core 9mer binding registers (in blue with P1 anchors highlighted red) were identified within the peptides. The p1 and p9 anchor residues are indicated in the figure and peptides that stimulated positive T cell responses in both non-adjusted and adjusted data sets are indicated (✓). 15

Figure 5. Potential core 9mer HLA-DR restricted epitope within peptides 77, 78, 79 and 80. Using iTope™ four possible core 9mer binding registers (in blue with P1 anchors highlighted red) were identified within the peptides. The p1 and p9 anchor residues are indicated in the figure and peptides that stimulated positive T cell responses in both non-adjusted and adjusted data sets are indicated (✓). 16

1. Summary

Cleveland BioLabs, Inc. provided the sequence of 80 peptides, each 15 amino acids in length, to be analysed for the presence of $CD4^+$ T cell epitopes using EpiScreen™ T cell epitope mapping technology. All peptides were synthesised and tested against PBMC from a cohort of 50 healthy human donors. $CD4^+$ T cell responses against individual peptides were measured using proliferation assays (^{3}[H]-thymidine incorporation). Positive responses were observed to five peptides containing HLA-DR restricted MHC class II binding motifs.

2. Introduction

Immune responses to biological therapeutic agents are wide ranging, and can be directed against agents that are both non-human and human in origin. These responses include those that elicit a weak clinical effect and those that limit efficacy which can occasionally result in morbidity or even mortality in patients. In particular, serious complications can arise with the production of neutralising antibodies, especially when they target recombinant self-proteins and therefore have the potential to cross react with the patient’s own endogenous protein (Lim, 2005). Problems associated with immunogenicity to biologics, especially monoclonal antibodies, have been reduced largely due to advances in molecular biology. There are, however, many recombinant protein biologics that are identical to endogenously expressed human sequences that still elicit potent neutralising immune responses in patients (Hochuli 1997, Schellekens *et al* 1997, Namaka *et al* 2006). The mechanism by which immunogenicity is triggered remains unclear although the tolerance to self-proteins may be broken by a number of factors linked to both the product and the patient (reviewed in Chester *et al* 2005, Baker and Jones 2007). For the product, these include dose, frequency of administration, route, immunomodulatory capacity of the protein therapeutic, and the formulation (Jaber and Baker 2007). For the patient, factors such as immune competence (i.e. whether the patient is receiving immunosuppressive treatment), patient’s MHC haplotype and intrinsic tolerance to the protein therapeutic will influence immunogenicity. Regardless of how immunogenicity is triggered, one of the single most important factors in the development of an ensuing immune response is the presence of epitopes that are able to effectively stimulate a potent $CD4^+$ T cell response (reviewed Baker and Jones 2007).

T cell epitope analysis is becoming increasingly important particularly in the pre-clinical analysis of biologics and may, in time, become a requirement for regulatory approval for clinical trials. To this end, Antitope has developed a pre-clinical *ex vivo* T cell assay (EpiScreen™) that provides an effective technology for predicting T cell immunogenicity by identifying linear T cell epitopes present in protein sequences. Synthetic overlapping peptides ranging from 15 to 35 amino acids in length are tested against a cohort of community blood donors carefully selected based on MHC haplotypes to provide a quantitative analysis of T cell epitopes present in protein sequences. This technology has been used successfully to compare protein variants for the potential to induce an immune response *in vivo*. EpiScreen™ provides a powerful screening technology due to the high degree of sensitivity along with the robust nature of the assay which allows an accurate pre-clinical assessment of the potential for immunogenicity of biologics.

In the present study, CBL01, 80 peptides were tested in the EpiScreen™ T cell epitope mapping assay using PBMC from a cohort of 50 healthy donors. Individual peptides were tested in sextuplicate cultures and $CD4^+$ T cell proliferation responses were assessed using the EpiScreen™ T cell epitope mapping technology in order to identify the location of epitopes, as well as their relative potency.

3. Methods

3.1 EpiScreen™ Donor Selection

Antitope Ltd is licensed (number 12627) by the Human Tissue Authority, the regulatory body for the Human Tissue Act 2004. This act regulates the removal, storage, use and disposal of human tissue within the UK. Accordingly all activities that fall under this act were performed to HTA standards.

PBMC were isolated from healthy community donor buffy coats (from blood drawn within 24 hours) obtained under consent from commercial vendors. Cells were separated by Lymphoprep (Axis-shield, Dundee, UK) density centrifugation and $CD8^+$ T cells were depleted using $CD8^+$ RosetteSep™ (StemCell Technologies Inc, London, UK). Donors were characterised by identifying HLA-DR haplotypes using the HISTO Spot SSO HLA typing method (MC Diagnostics, St. Asaph, UK). T cell responses to a control neoantigen protein (KLH, Sigma, Poole, UK) and control peptides derived from IFV (C32) and EBV (C3) were also determined. PBMC were then frozen and stored in liquid nitrogen until required.

A cohort of 50 donors was selected to best represent the number and frequency of HLA-DR and DQ allotypes expressed in the world population. Analysis of the allotypes expressed revealed that the cohort covered all major HLA-DR and DQ allotypes. **Figure 1** shows a comparison of the distribution and frequency of MHC class II haplotypes expressed in the world, European and North American populations against the selected donor cohort (CBL01).

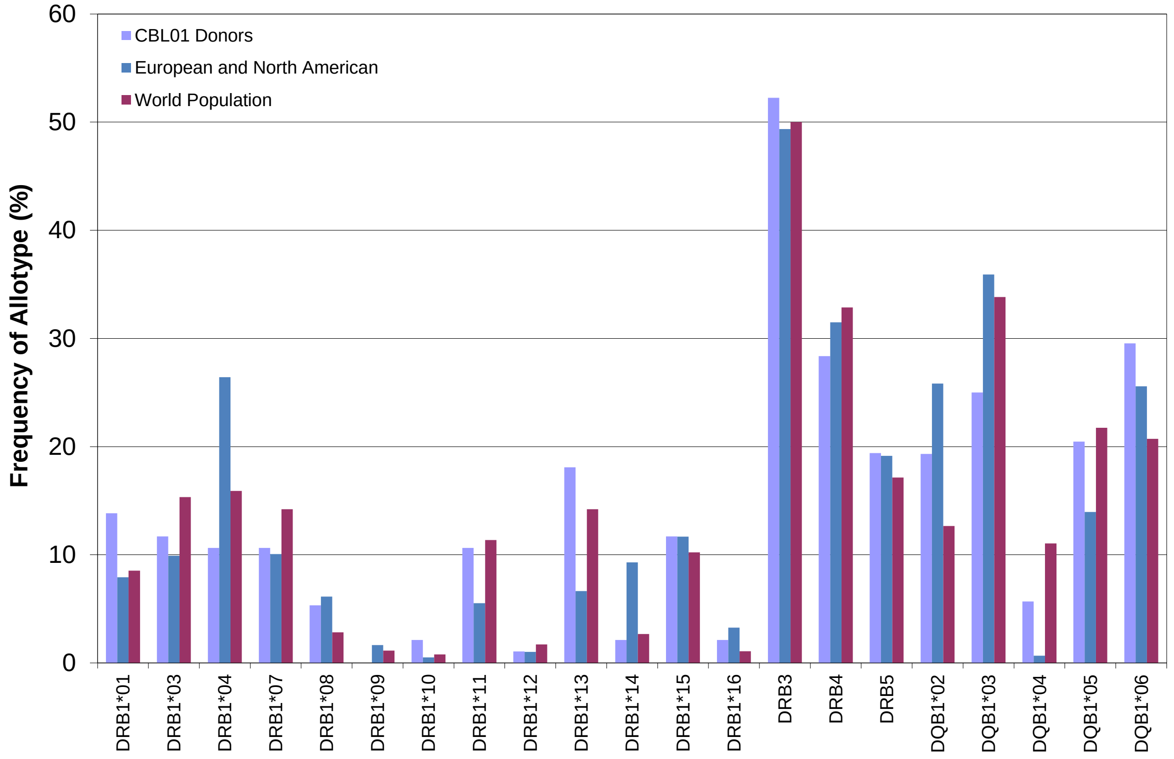

Figure 1. Comparison of the frequency of donor allotypes expressed in the CBL01 study cohort (n=50) with the European/North American and world population.

3.2 EpiScreen™Analysis: Proliferation Assay

PBMC from each donor were thawed, counted and viability was assessed. Cells were revived in room temperature AIM V® culture medium (Invitrogen, Paisley, UK) before adjusting the cell density to 2.5-3.5x10^6 PBMC/ml (proliferation cell stock). Peptides were synthesised on a 1-3 mg scale with free N-terminal amine and C-terminal carboxylic acid. Peptides were dissolved in DMSO to a concentration of 10 mM and peptide culture stocks prepared by diluting into AIM V® culture medium to a final concentration of 5 µM in each well. For each peptide and each donor, sextuplicate cultures were established in a flat bottomed 96 well plate. Both positive and negative control cultures were also tested in sextuplicate. For each donor, three controls (KLH protein (final assay concentration 0.3 µM) and peptides derived from IFV and EBV) were also included. For a positive control, PHA (Sigma, Poole, UK) was used at a final concentration of 2.5 µg/ml.

Cultures were incubated for a total of 6 days before adding 0.75 µCi ^{3}[H]-thymidine (Perkin Elmer®, Beaconsfield, UK) to each well. Cultures were incubated for a further 18 hours before harvesting onto filter mats using a TomTec Mach III cell harvester. CPM for each well were determined by Meltilex™ (Perkin Elmer®, Beaconsfield, UK) scintillation counting on a Microplate Beta Counter (Perkin Elmer®, Beaconsfield, UK) in paralux, low background counting mode.

3.3 EpiScreen™ Data Analysis

For proliferation assays, an empirical threshold of a stimulation index (SI) equal to or greater than 2 (SI ≥2.00) has been previously established whereby samples inducing proliferative responses above this threshold are deemed positive (where included, borderline SI ≥1.90 are highlighted). Extensive assay development and previous studies have shown that this is the minimum signal to noise threshold allowing maximum sensitivity without detecting large numbers of false positive responses. Positive responses are defined by the following statistical and empirical thresholds:

1. Significance (p <0.05) of the response by comparing cpm of test wells against medium control wells using unpaired two sample Student's t-test.

2. Stimulation index greater than 2.00 (SI ≥2.00), where SI = mean cpm of test wells /mean cpm medium control wells. Data presented in this way is indicated as SI ≥2.00, p <0.05.

In addition, intra-assay variation was assessed by calculating the CV and SD of the raw data from replicate cultures. Proliferation assays were set up in sextuplicate cultures ("non-adjusted data"). To ensure that intra-assay variability was low, the data were also analysed after removing the maximum and minimum cpm values ("adjusted data") and the SI of donor responses was compared using both data sets. Details of donor SI from both non-adjusted and adjusted data sets can be found in **Appendix I** and **Appendix II**, respectively. T cell epitopes were identified by the frequency of responses to a peptide being ≥ 6%, based on the average

frequency of the positive responses to all peptides in the study plus 1.5x SD. Any peptide that induced proliferative responses above this threshold in both the adjusted and non-adjusted data was considered to contain a T cell epitope. However for interpretation, weight is added to overlapping peptides that induce positive T cell responses in a common group of donors versus single peptides with unique donors.

3.4 Endotoxin testing of peptides

Peptides were screened for endotoxin contamination using a LAL Chromogenic Endotoxin Quantitation Kit (Pierce (Perbio), Cramlington, UK). Briefly, 1 µl of stock peptide (10 mM), diluted in 49 µl of the provided endotoxin free H_2O, was incubated with 50 µl LAL substrate for 10 minutes at 37°C in a pre-warmed flat bottomed 96 well plate. After exactly 10 minutes, 100 µl of chromogenic substrate was added to each well. This was incubated at 37°C for a further 6 minutes before the addition of 50 µl of Stop Reagent (25% acetic acid in endotoxin free H_2O). The absorbance at 405 nm was then measured, and a standard curve was prepared by plotting the average blank-corrected absorbance for each standard versus its concentration in EU/ml. The formulated standard curve was used to determine the endotoxin concentration of each sample. All peptides contained levels of endotoxin below acceptable limits (<5 EU/mg).

3.5 *In Silico* Analysis of Peptides

The sequences of peptides that were positive in the proliferation assay were analysed using Antitope's predictive iTope™ software (Perry *et al* 2008). This software predicts favourable interactions between amino acid side chains of the peptide and specific binding pockets within the MHC class II binding groove. Each peptide was tested against Antitope's database of MHC class II alleles (34 in total) and scored based on their fit and interactions with the MHC class II molecules.

4. Results and Discussion

A total of 80 peptides were synthesised from sequences supplied by Cleveland BioLabs, Inc. These peptides were tested for the presence of $CD4^+$ T cell epitopes by EpiScreen™ T cell epitope mapping analysis. Positive T cell responses were defined by donors whose PBMC produced a significant (p <0.05) response with a SI ≥2.00 to any given peptide. Based on previous EpiScreen™ T cell epitope mapping studies, T cell epitopes were identified by calculating the average frequency of the positive responses to all peptides in the study plus 1.5x SD (termed 'background response threshold') (**Figure 2**). Thus, peptides were considered to contain a T cell epitope if they induced positive T cell proliferation responses (SI ≥2.00, p <0.05, including borderline responses SI ≥1.90, p <0.05) in 3 or more donors in both the non-adjusted and adjusted data sets (≥ 6% of the donor cohort).

All donors produced a positive T cell response against the positive control PHA in the proliferation assay indicating that cells in the *ex vivo* cultures were functional (data not shown). In the non-adjusted dataset the frequencies of T cell responses against the control neoantigen KLH, and control peptides C3 (EBV EBNA protein derived epitope) and C32 (IFV HA protein derived epitope) were 92%, 22% and 12% respectively, the same frequencies were observed in the adjusted set, within the typical ranges seen in previous T cell epitope mapping studies.

The output from non-adjusted and adjusted data analysis was examined to ensure that intra-assay variability was low and that positive responses were not the result of spurious proliferation in individual wells. The results from each analysis showed, in most cases, only small differences between donor responses for both non-adjusted and adjusted data sets (**Appendix I** and **Appendix II**). **Table 1** provides a summary of individual donor responses to each of the peptides. The frequency of positive donor responses to each peptide in the T cell proliferation assay is shown in **Figure 2**. A total of five peptides induced positive responses at or above the 6% cut off in both the non-adjusted and adjusted data sets.

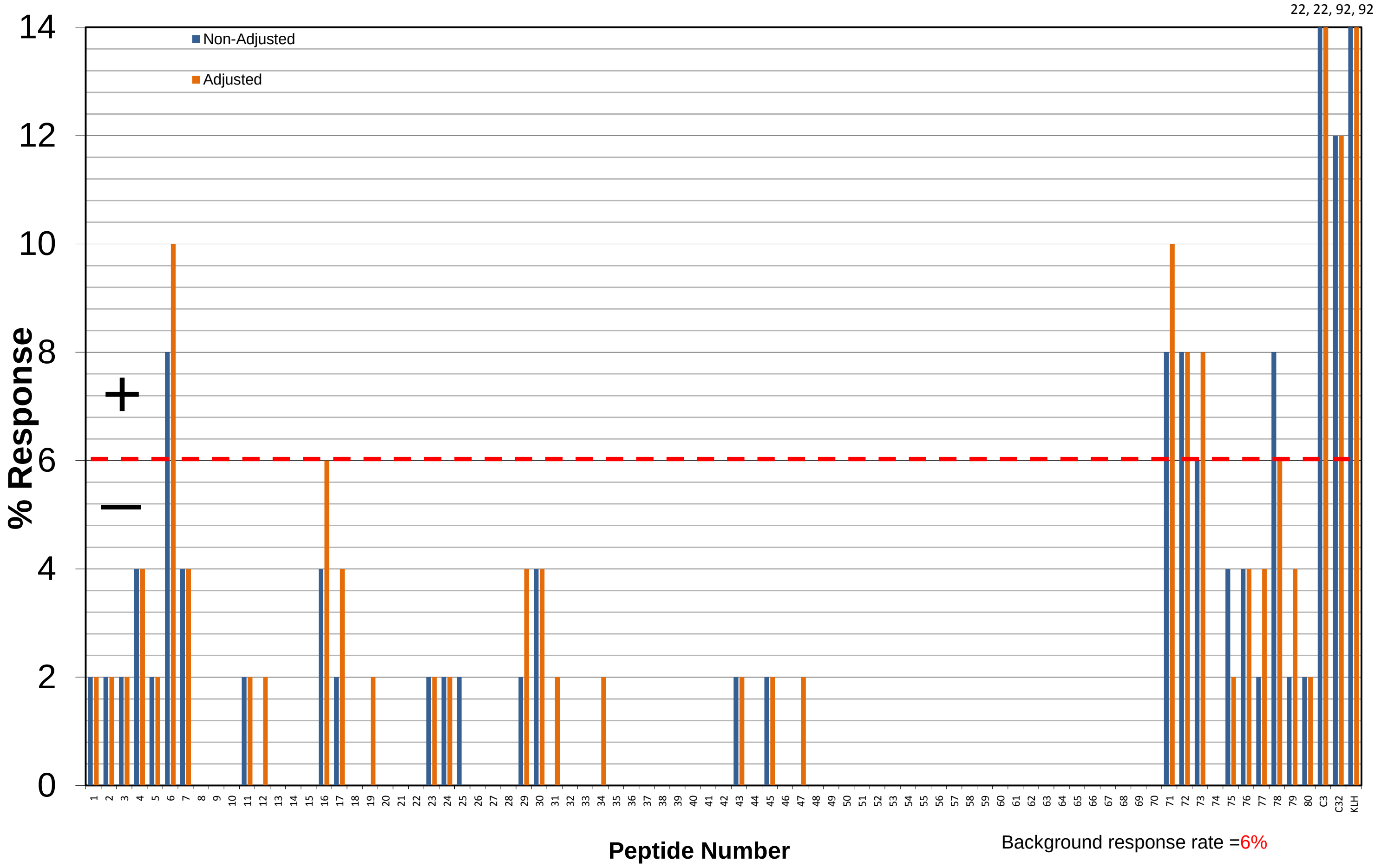

Figure 2. CD4+ T cell epitope map using peptides tested against PBMC from 50 healthy donors. The non-adjusted and adjusted proliferation assay data for the 80 test peptides and controls C3, C32 and KLH. Peptides inducing positive (SI ≥2.00, *p* <0.05) T cell proliferation responses at a frequency above the background response threshold (indicated by the red dotted line) contain T cell epitopes. KLH induced positive responses (SI ≥2.00, *p* <0.05) in 92% of donors in both the non-adjusted and adjusted data sets.

4.1 Epitope 1

Peptide 6 stimulated a frequency of T cell proliferation (SI ≥2.00, *p* <0.05, including borderline responses SI ≥1.90) above the background threshold (**Figure 2**), inducing positive responses in 8% and 10% of the study cohort in the non-adjusted and adjusted data sets respectively (**Figure 2**, **Table 1** and **Table 2**). The mean magnitude of the positive responses to this peptide was low, with mean SIs ranging between 2.35 and 2.57 for the non-adjusted and adjusted data sets, suggesting that this is a weak T cell epitope (mean SI <3.00) (**Table 2**). *In silico* MHC class II binding analysis using iTope™ revealed two possible overlapping core 9mer HLA-DR restricted epitopes in peptide 6, IANRFTSNI and FTSNIKGLT (**Figure 3**). The 9mers were predicted to bind 17 and 8 out of 34 MHC class II alleles respectively. Since sub threshold responses were detected to peptides 5 and 7 which contain either the IANRFTSNI 9mer (peptide 5) or FTSNIKGLT 9mer (peptide 7) it is not possible to add weighting to either 9mer being the core sequence responsible for the T cell epitope.

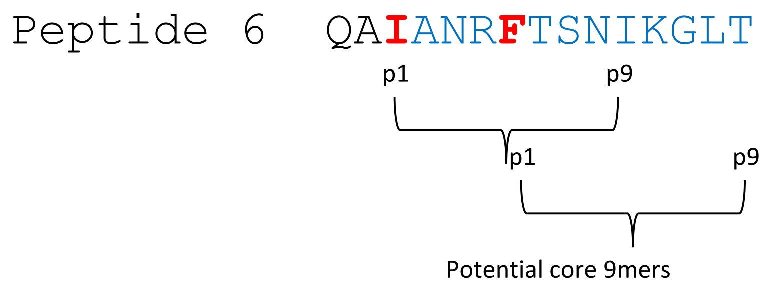

Figure 3. Potential core 9mer HLA-DR restricted epitope within peptide 6. Using iTope™ two possible core 9mer binding registers (in blue with P1 anchors highlighted red) were identified within the peptide. The p1 and p9 anchor residues are indicated in the figure.

4.2 Epitope 2

Peptides 71, 72 and 73 all stimulated a frequency of positive responses (SI ≥2.00, *p* <0.05, including borderline responses SI ≥1.90) above the background threshold (**Figure 2**). Peptide 71 induced positive responses in 8% and 10% of the study cohort in the non-adjusted and adjusted data sets respectively, peptide 72 induced positive responses in 8% of the donor cohort in both data sets, and peptide 73 induced positive responses in 6 and 8% of the donor cohort in the non-adjusted and adjusted data sets respectively (**Figure 2**, **Table 1** and **Table 2**). The mean magnitude of the positive responses to these peptides was moderate, with mean SIs ranging between 2.08 and 5.24 for the non-adjusted and adjusted data sets (**Table 2**), suggesting that this is a strong T cell epitope, with peptide 72 stimulating the strongest responses. *In silico* MHC class II binding analysis using iTope™ revealed three possible core 9mer HLA-DR restricted epitopes, ILQQAGTSV, LQQAGTSVL and VLAQANQVP (**Figure 4**). These 9mers were predicted to bind 2, 29 and 9 out of 34 MHC class II alleles respectively. The mean magnitude SI together with the *in silico* data and the fact that donors 25, 33 and 44 responded to both peptides 71 and 72 suggest that the core 9mer LQQAGTSVL is most likely to be the T cell epitope in peptides 71 and 72. Potentially due to the 9 amino acid overlap between peptides 71, 72 and 73, there may be a single common 9mer, potentially presented via HLA-DP/DQ, stimulating the positive responses to all three peptides. Alternatively, peptides 72 and 73 may contain an additional common 9mer,

potentially presented via HLA-DP/DQ, since peptide 74, which also contains the HLA-DR predicted 9mer VLAQANQVP, was negative; however it is possible that the lack of C-terminal flanking residues negatively influencing MHC class II binding may have led to the positive responses in peptide 73 which were absent in peptide 74 (Godkin *et al* 2001, Arnold *et al* 2002, Sant'Angelo *et al* 2003, Zavala-Ruiz *et al* 2004, Lovitch *et al* 2006, Knapp *et al* 2009).

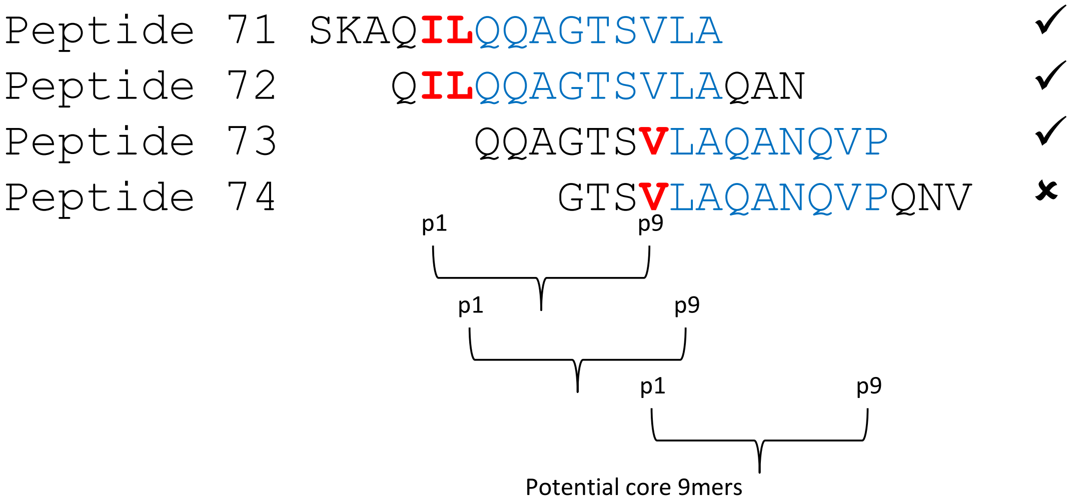

Figure 4. Potential core 9mer HLA-DR restricted epitope within peptides 71, 72, 73 and 74. Using iTope™ three possible core 9mer binding registers (in blue with P1 anchors highlighted red) were identified within the peptides. The p1 and p9 anchor residues are indicated in the figure and peptides that stimulated positive T cell responses in both non-adjusted and adjusted data sets are indicated (✓).

4.3 Epitope 3

Peptide 78 stimulated a frequency of T cell proliferation (SI ≥2.00, p <0.05, including borderline responses SI ≥1.90) above the background threshold (**Figure 2**), inducing positive responses in 8% and 6% of the study cohort in the non-adjusted and adjusted data sets respectively (**Figure 2**, **Table 1** and **Table 2**). The mean magnitude of the positive responses to this peptide was moderate, with mean SIs ranging between 2.61 and 3.05 for the non-adjusted and adjusted data sets suggesting this is a moderate T cell epitope (**Table 2**). *In silico* MHC class II binding analysis using iTope™ revealed four possible core 9mer HLA-DR restricted epitopes VLSLLVPRG, LSLLVPRGS, LLVPRGSHH and LVPRGSHHH (**Figure 5**). The 9mers were predicted to bind 29, 29, 2 and 30 out of 34 MHC class II alleles respectively. Since both peptides 77 and 79 induced sub-threshold responses, it is difficult to identify which 9mer may correspond to the T cell epitope in peptide 78. Consideration of the influence of flanking residues (Godkin *et al* 2001, Arnold *et al* 2002, Sant'Angelo *et al* 2003, Zavala-Ruiz *et al* 2004, Lovitch *et al* 2006, Knapp *et al* 2009) may point to the core 9mer LSLLVPRGS which lacks either N- or C-terminal flanking residues in peptides 77 and 79 respectively. Regardless of the precise core 9mer, the presence of the hexahistidine tag appears to have contributed to the formation of a novel T cell epitope.

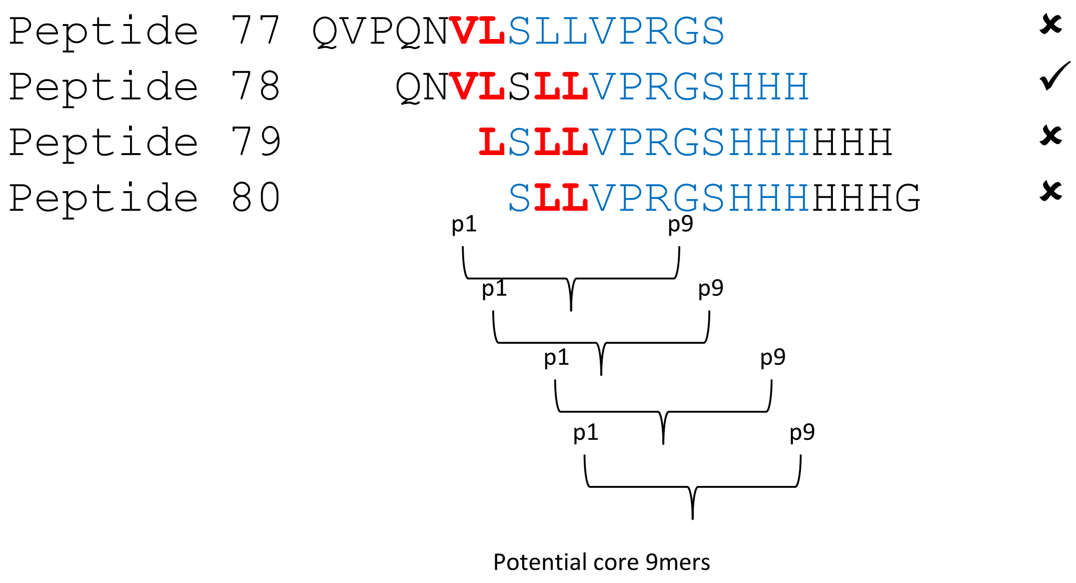

Figure 5. Potential core 9mer HLA-DR restricted epitope within peptides 77, 78, 79 and 80. Using iTope™ four possible core 9mer binding registers (in blue with P1 anchors highlighted red) were identified within the peptides. The p1 and p9 anchor residues are indicated in the figure and peptides that stimulated positive T cell responses in both non-adjusted and adjusted data sets are indicated (✓).

Peptide	Proliferation Non-adjusted	Proliferation Adjusted	Peptide Sequence
1	16(1.96)	16(1.92)	MSGLRINSAKDDAAG
2	9(1.96)	9(1.91)	LRINSAKDDAAGQAI
3	22(3.10)	22(3.52)	NSAKDDAAGQAIANR
4	9(1.94), 22(3.84)	9(1.90), 22(3.88)	KDDAAGQAIANRFTS
5	21(1.90)	22(2.04)	AAGQAIANRFTSNIK
6	9(1.90), 33(1.94), 39(2.77), 44(2.77)	9(1.91), 33(1.93), 39(3.79), 43(2.22), 44(2.98)	QA**I**ANR**F**TSNIKGLT
7	21(2.00), 43(2.39)	21(2.08), 43(2.51)	ANRFTSNIKGLTQAS
8			FTSNIKGLTQASRNA
9			NIKGLTQASRNAADG
10			GLTQASRNAADGISI
11	10(2.45)	10(2.45)	QASRNAADGISIAQT
12		1(1.90)	RNAADGISIAQTTEG
13			ADGISIAQTTEGALN
14			ISIAQTTEGALNEIN
15			AQTTEGALNEINNNL
16	11(2.18), 22(6.78)	7(1.90), 11(2.09), 22(6.45)	TEGALNEINNNLQRV
17	22(3.07)	20(1.96), 22(3.17)	ALNEINNNLQRVREL
18			EINNNLQRVRELSVQ
19		20(1.93)	NNLQRVRELSVQATA
20			QRVRELSVQATAGAN
21			RELSVQATAGANADA
22			SVQATAGANADAALK
23	20(2.24)	20(2.37)	ATAGANADAALKAIQ
24	20(2.32)	20(2.45)	GANADAALKAIQAEI
25	1(5.86)		ADAALKAIQAEIQQR
26			ALKAIQAEIQQRLEE
27			AIQAEIQQRLEEIDR
28			AEIQQRLEEIDRVSQ
29	7(1.99)	7(2.08), 8(2.02)	QQRLEEIDRVSQQTQ
30	7(1.91), 50(1.98)	7(1.97), 50(1.97)	LEEIDRVSQQTQAAA
31		7(1.90)	IDRVSQQTQAAAVKV
32			VSQQTQAAAVKVLSQ
33			QTQAAAVKVLSQDNA
34		6(1.99)	AAAVKVLSQDNAMAI
35			VKVLSQDNAMAIQVG

Peptide	Proliferation Non-adjusted	Proliferation Adjusted	Peptide Sequence
36			LSQDNAMAIQVGAND
37			DNAMAIQVGANDGAA
38			MAIQVGANDGAAITI
39			QVGANDGAAITIDLQ
40			ANDGAAITIDLQKID
41			GAAITIDLQKIDVKS
42			ITIDLQKIDVKSLGL
43	30(2.69)	30(2.89)	DLQKIDVKSLGLDGF
44			KIDVKSLGLDGFNVN
45	30(2.07)	30(2.18)	VKSLGLDGFNVNSPG
46			LGLDGFNVNSPGSTA
47		8(1.97)	DGFNVNSPGSTANPL
48			NVNSPGSTANPLASI
49			SPGSTANPLASIDSA
50			STANPLASIDSALSK
51			NPLASIDSALSKVDA
52			ASIDSALSKVDAVRS
53			DSALSKVDAVRSSLG
54			LSKVDAVRSSLGAIQ
55			VDAVRSSLGAIQNRF
56			VRSSLGAIQNRFDSA
57			SLGAIQNRFDSAITN
58			AIQNRFDSAITNLGN
59			NRFDSAITNLGNTVT
60			DSAITNLGNTVTNLN
61			ITNLGNTVTNLNSAR
62			LGNTVTNLNSARSRI
63			TVTNLNSARSRIEDA
64			NLNSARSRIEDADYA
65			SARSRIEDADYATEV
66			SRIEDADYATEVSQM
67			EDADYATEVSQMSKA
68			DYATEVSQMSKAQIL
69			TEVSQMSKAQILQQA
70			SQMSKAQILQQAGTS

Peptide	Proliferation Non-adjusted	Proliferation Adjusted	Peptide Sequence
71	13(1.96), 25(2.20), 33(4.80), 44(3.96)	13(2.02), 25(2.16), 33(4.71), 42(1.90), 44(3.60)	SKAQ**IL**QQAGTS**V**LA
72	2(2.2.69), 25(2.55), 33(4.89), 44(10.79)	2(2.32), 25(2.44), 33(4.50), 44(10.60)	Q**IL**QQAGTS**V**LAQAN
73	2(2.46), 16(2.05), 42(2.14)	2(2.25), 7(1.92), 16(2.03), 42(2.11)	QQAGTS**V**LAQANQVP
74			GTSVLAQANQVPQNV
75	1(2.67), 2(2.07)	2(2.06)	VLAQANQVPQNVLSL
76	5(1.95), 33(3.65)	5(1.92), 33(3.84)	QANQVPQNVLSLLVP
77	12(2.72)	5(1.90), 12(2.60)	QVPQNVLSLLVPRGS
78	12(3.59), 23(1.91), 33(2.22), 39(2.70)	12(4.22), 33(2.21), 39(2.70)	QN**VL**S**LL**VPRGSHHH
79	42(2.32)	42(2.14), 48(1.93)	LSLLVPRGSHHHHHH
80	42(2.03)	42(2.13)	SLLVPRGSHHHHHHG

Table 1. Summary of individual donor responses to 80 peptides. Positive responses (SI ≥2.00, *p* <0.05, including borderline responses SI ≥1.90) are indicated by the donor number and individual SI are shown in brackets next to the corresponding donor. The background response rate was 4.3% in the non-adjusted data and 4.9% in the adjusted data. Peptides inducing positive T cell proliferation above this frequency (positive response in ≥3 donors) contained T cell epitopes (indicated in yellow). Potential p1 anchors are highlighted in red (predicted by iTope™).

Peptide	Peptide Sequence	Response Frequency (%)		Mean SI (±SD)		Epitope ID
		Non Adjusted	Adjusted	Non Adjusted	Adjusted	
6	QA**I**ANR**F**TSNIKGLT	8	10	2.35(±0.49)	2.57(±0.81)	1
71	SKAQ**IL**QQAGTS**V**LA	8	10	3.23(±1.37)	2.88(±1.23)	2
72	Q**IL**QQAGTS**V**LAQAN	8	8	5.23(±3.86)	4.97(±3.89)	3
73	QQAGTS**V**LAQANQVP	6	8	2.22(±0.22)	2.08(±0.14)	3
78	QN**VL**S**LL**VPRGSHHH	8	6	2.61(±0.73)	3.04(±1.05)	3

Table 2. Summary of magnitude (mean SI and standard deviation) and frequency (% donor response) of positive T cell proliferation responses. Potential P1 anchor residues are shown in red (predicted by iTope™).

5. Conclusion

EpiScreen™ T cell epitope mapping of 80 peptides resulted in positive T cell responses against five peptides (**Figure 2**, **Table 1** and **Table 2**). The magnitude and frequency of responses to the peptides suggested one weak, one moderate and one strong T cell epitope. Sequence analysis of the positive peptides using iTope™ identified several potential HLA-DR restricted MHC class II binding motifs.

6. References

Arnold PY, La Gruta NL, Miller T, Vignali KM, Adams PS, Woodland DL, Vignali DA. The majority of immunogenic epitopes generate CD4+ T cells that are dependent on MHC class II-bound peptide-flanking residues. *J Immunol*. 2002 **169**(2):739-49. (Erratum in: *J Immunol* 2002 169(8):4674).

Baker MP and Jones TD. Identification and removal of immunogenicity in therapeutic proteins. *Curr. Opin. Drug. Disc. Dev*. 2007 **10**(2): 219-227.

Chester, K, Baker, MP and Mayer A. Overcoming the immunologic response to foreign enzymes in cancer therapy. *Expert Rev. Clin. Immunol*. 2005 **1**(4): 549-559.

Godkin AJ, Smith KJ, Willis A, Tejada-Simon MV, Zhang J, Elliott T, Hill AV. Naturally processed HLA class II peptides reveal highly conserved immunogenic flanking region sequence preferences that reflect antigen processing rather than peptide–MHC interactions. *J Immunol*. 2001 **166**:6720–7.

Hochuli E. Interferon immunogenicity: technical evaluation of interferon-alpha 2a. *J Interferon Cytokine Res*. 1997 **17** Suppl 1:S15-21.

Jaber A and Baker MP. Assessment of the immunogenicity of different interferon beta-1a formulations using ex vivo T-cell assays. *J Pharm Biomed Anal* 2007 **43**(4):1256-61.

Knapp B, Omasits U, Bohle B, Maillere B, Ebner C, Schreiner W, Jahn-Schmid B. 3-Layer-based analysis of peptide-MHC interaction: in silico prediction, peptide binding affinity and T cell activation in a relevant allergen-specific model. *Mol Immunol.* 2009 **46**(8-9):1839-44.

Lim LC. Acquired red cell aplasia in association with the use of recombinant erythropoietin in chronic renal failure. *Hematology*. 2005 **10**(3):255-9.

Lovitch SB, Pu Z, Unanue ER. Amino-terminal flanking residues determine the conformation of a peptide–class II MHC complex. *J Immunol*. 2006 **176**:2958–68.

Namaka M, Pollitt-Smith M, Gupta A, Klowak M, Vasconcelos M, Turcotte D, Gong Y, Melanson M. The clinical importance of neutralizing antibodies in relapsing-remitting multiple sclerosis. *Curr Med Res Opin.* 2006 **22**(2):223-39.

Perry LC, Jones TD and Baker MP. New approaches to prediction of immune responses to therapeutic proteins during preclinical development. *Drugs R D*. 2008 **9**(6):385-96.

Sant'Angelo DB, Robinson E, Janeway CA Jr, Denzin LK. Recognition of core and flanking amino acids of MHC class II-bound peptides by the T cell receptor. *Eur J Immunol.* 2002 **32**(9):2510-20.

Schellekens, H., Ryff, J.C., and Van Der Meide, P.H. Assays for antibodies to human interferon-alpha: the need for standardization. *J. Interferon Cytokine Res*. 1997 **17**(Suppl. 1), S5–S8.

Zavala-Ruiz, Z, Strug, I, Anderson, MW, Gorski, J and Stern, LJ. A polymorphic pocket at the p10 position contributes to peptide binding specificity in class II MHC proteins. *Chemistry & Biology*. 2004 **11**(10):1395-402.

7. Appendix I

Mean SI proliferation assay data from donors 1-25 in the non-adjusted data set for peptides 1-42. Numbers in red indicate positive responses (SI ≥2.00, p <0.05). Numbers in pink indicate borderline responses (SI ≥1.90, p <0.05).

PEPTIDE	1	2	3	4	5	6	7	8	9	10	11	12	13	14	15	16	17	18	19	20	21	22	23	24	25
1	0.99	0.78	0.86	1.21	1.13	0.59	1.63	1.40	1.54	1.83	0.90	1.09	1.05	1.26	0.93	1.96	0.86	1.10	1.10	0.87	1.00	1.26	0.76	0.85	1.19
2	1.39	0.91	1.12	1.33	1.06	1.05	1.54	0.98	1.96	1.59	1.08	0.99	1.07	1.15	1.05	1.43	0.95	1.15	1.18	0.86	1.17	1.17	0.90	1.06	1.17
3	1.33	0.93	0.95	1.17	0.84	1.17	1.48	1.11	1.66	1.61	0.97	0.76	0.84	1.31	1.14	1.02	1.11	0.98	1.22	1.11	1.30	3.10	0.85	1.06	1.28
4	1.08	0.86	0.92	1.47	0.87	1.26	1.48	1.01	1.94	1.73	1.28	1.34	0.89	1.34	1.08	0.85	0.95	0.97	1.04	1.03	1.84	3.84	0.91	1.15	1.11
5	1.22	0.80	0.20	1.30	1.14	1.38	1.50	0.97	1.64	1.37	1.34	0.78	0.82	1.35	1.10	0.86	1.01	1.02	0.93	0.93	1.90	1.15	0.86	1.19	1.26
6	1.11	0.93	0.23	1.14	0.94	1.68	1.60	0.98	1.90	1.02	1.03	0.93	0.86	1.35	1.21	1.06	1.07	0.91	0.83	0.71	1.47	1.11	1.05	1.15	1.27
7	1.03	1.02	0.31	1.30	1.10	1.42	1.36	0.83	1.05	0.98	1.09	1.15	0.97	0.97	1.02	1.08	0.78	0.69	0.95	0.63	2.00	1.37	1.15	1.24	1.18
8	0.64	1.00	0.48	1.33	1.41	1.30	0.84	0.93	1.62	0.95	0.98	1.09	1.29	0.99	0.98	1.25	0.76	0.83	1.24	0.70	1.76	1.19	1.04	1.06	1.19
9	1.08	0.74	0.97	1.34	1.27	0.66	1.76	1.34	1.31	1.63	0.90	1.12	1.01	1.42	1.16	1.31	0.94	1.17	0.91	1.02	0.92	1.61	0.78	1.19	1.14
10	1.50	1.06	1.09	1.39	0.99	0.89	1.49	1.16	1.25	1.68	0.96	1.07	1.01	1.17	1.10	1.09	1.00	1.07	0.82	0.89	1.24	1.49	0.81	1.19	1.18
11	1.21	0.98	0.96	1.18	1.01	0.98	1.61	1.05	1.13	2.45	0.94	0.84	0.91	1.25	1.19	1.06	0.98	1.28	0.83	1.08	1.69	1.40	0.80	1.22	1.10
12	1.72	0.93	0.80	1.19	1.17	1.03	1.29	1.07	1.09	1.72	1.06	0.90	1.04	1.17	0.96	0.98	1.19	1.24	1.04	1.35	1.14	1.28	0.87	1.21	1.20
13	1.26	1.14	0.35	1.31	1.26	1.08	1.37	1.14	1.05	1.30	1.45	0.84	1.01	1.62	1.27	1.30	1.17	1.04	0.85	0.85	1.17	1.69	0.97	1.33	1.09
14	1.19	1.21	0.59	1.43	1.01	1.11	1.50	0.96	1.22	1.05	1.49	1.18	0.98	1.34	1.16	1.28	1.12	1.00	1.25	0.93	1.05	1.22	0.85	1.18	1.12
15	1.08	0.98	0.70	0.87	1.50	0.72	1.30	1.51	1.24	1.17	1.70	0.63	0.63	0.98	0.86	0.70	0.90	1.09	1.16	1.69	0.92	0.91	1.17	0.86	0.80
16	1.12	1.26	0.81	0.87	1.21	1.35	1.86	1.23	0.77	1.12	2.18	0.63	0.67	0.93	1.16	0.83	0.91	0.74	1.23	1.26	1.18	6.78	1.24	1.02	0.94
17	1.43	0.99	0.93	0.79	1.22	1.08	1.40	1.36	1.40	1.02	1.48	0.69	0.73	1.34	1.20	1.02	1.31	1.05	1.31	1.82	0.91	3.07	1.24	0.96	1.00
18	1.24	0.86	0.80	1.23	0.85	1.21	1.51	1.26	1.19	0.85	1.57	0.71	0.60	0.99	1.24	0.80	1.38	0.80	1.10	1.81	0.99	0.83	1.27	0.93	1.00
19	1.14	0.74	0.86	1.18	1.10	1.18	1.20	1.10	1.03	0.87	1.47	0.94	0.73	1.04	1.06	0.96	1.39	0.99	0.71	1.81	0.93	0.83	1.36	0.95	1.40
20	1.21	1.02	0.76	1.30	0.94	1.25	1.02	1.07	1.40	0.99	1.56	0.91	0.73	1.33	0.96	1.04	1.12	0.94	0.98	1.55	0.85	0.98	1.32	0.99	1.14
21	1.21	0.97	0.87	1.14	1.23	1.05	1.05	1.05	1.38	0.84	1.57	0.93	0.81	0.92	0.92	0.89	1.29	0.88	0.99	1.50	0.65	0.93	1.22	0.80	1.13
22	0.79	0.96	0.59	1.09	1.12	1.22	1.08	1.31	1.00	0.69	1.33	1.01	0.86	1.01	0.79	1.17	1.05	0.95	1.15	1.48	0.97	0.91	1.13	0.89	1.25
23	0.95	0.92	0.75	0.73	1.22	0.90	1.17	1.52	1.44	1.37	1.21	0.58	0.81	0.91	0.96	0.88	0.95	1.18	1.13	2.24	1.17	0.93	1.29	1.28	0.90
24	1.03	1.28	0.97	0.78	1.14	0.87	1.34	1.37	1.28	0.90	1.24	0.78	0.87	0.94	1.08	1.20	1.33	1.07	0.98	2.32	0.99	0.81	1.08	1.31	0.79
25	5.86	0.95	1.02	0.93	0.90	0.86	1.11	1.12	1.04	0.94	1.22	0.85	0.98	1.19	1.02	0.99	1.07	0.97	0.96	1.65	1.27	1.10	1.47	1.12	1.06
26	1.08	0.87	0.84	0.87	1.03	0.96	1.12	0.99	1.10	0.88	1.41	0.81	0.76	1.03	1.13	1.06	1.21	1.07	0.85	1.65	1.18	0.86	1.52	1.20	1.12
27	0.90	0.86	0.73	0.88	1.15	1.05	1.03	0.97	1.25	0.88	1.21	0.80	0.84	1.17	1.06	0.93	1.19	0.86	1.09	1.64	1.07	0.99	1.09	1.24	0.84
28	0.91	0.99	0.77	1.00	0.98	1.01	1.18	1.22	1.10	0.79	1.20	0.84	1.06	0.96	0.96	1.02	1.11	0.99	1.09	1.14	0.94	0.94	1.00	1.22	1.01
29	1.18	1.02	0.89	0.82	1.22	0.73	1.99	1.76	1.36	1.49	0.62	0.93	1.03	0.87	0.64	0.90	0.80	0.86	1.14	1.42	1.05	0.93	1.34	0.93	0.58
30	1.15	1.07	0.90	0.80	1.01	1.02	1.91	1.65	1.18	1.52	0.84	0.94	1.17	1.00	0.73	0.79	0.90	0.94	1.69	1.19	1.53	1.51	1.01	1.38	0.79
31	1.30	1.10	0.80	0.78	1.10	0.85	1.76	1.60	1.10	1.34	0.76	0.85	1.05	1.05	0.66	0.78	0.89	0.86	1.36	1.29	1.07	1.18	1.22	1.10	0.64
32	1.17	1.10	0.83	0.87	1.08	1.21	1.52	1.28	1.03	1.21	0.64	0.99	1.10	0.94	0.80	1.25	0.92	0.75	1.06	1.26	1.04	0.93	1.02	1.09	0.69
33	1.17	1.00	0.91	0.98	1.05	1.42	1.59	1.35	0.89	1.30	0.80	0.94	1.09	1.03	0.62	0.98	0.79	0.69	1.39	1.16	1.22	0.99	1.02	1.29	0.76
34	1.29	1.04	0.98	0.92	0.96	1.46	1.63	1.11	0.92	1.17	0.83	1.05	1.14	1.06	0.74	0.90	0.80	0.72	1.29	1.00	1.10	0.95	0.99	1.24	0.71
35	1.39	1.12	0.96	0.96	1.12	1.24	1.45	1.12	0.83	1.16	0.76	0.95	1.01	0.99	0.69	1.06	0.76	0.86	1.43	1.16	1.12	1.09	0.99	1.14	0.79
36	0.80	1.04	0.90	1.08	1.06	0.92	1.08	1.23	0.76	1.05	0.78	1.13	1.09	0.98	0.78	0.95	0.56	0.82	1.29	1.05	0.94	0.97	1.09	1.18	0.94
37	1.24	0.98	0.67	0.80	1.01	0.72	1.65	1.80	1.54	1.62	0.68	0.83	1.00	1.08	0.81	0.95	0.89	1.31	1.32	1.56	1.50	0.99	1.24	1.01	0.91
38	1.24	0.83	0.90	0.92	1.03	0.97	1.44	1.47	1.36	1.12	0.63	0.86	0.99	1.00	0.96	1.12	1.08	1.15	1.41	1.43	1.28	1.22	0.95	1.13	0.94
39	1.28	1.19	0.95	1.02	1.03	0.89	1.19	1.45	1.16	1.01	0.70	0.93	1.01	0.96	0.90	0.96	1.03	1.07	1.18	1.22	1.63	1.05	1.04	1.02	0.97
40	1.28	1.03	0.76	0.96	0.95	0.99	1.23	1.20	1.15	1.15	0.82	0.77	0.99	1.07	0.86	1.23	1.18	1.16	1.35	1.13	1.45	1.05	0.93	1.03	0.91
41	1.22	1.16	0.83	1.09	0.96	1.33	1.20	1.25	1.04	1.02	0.87	0.88	0.97	1.12	1.02	1.10	1.01	1.04	1.46	1.23	1.37	1.24	1.05	1.23	0.79
42	1.23	1.03	0.86	1.04	0.96	1.24	1.24	1.07	1.14	0.82	0.80	1.01	0.94	0.96	1.05	1.14	0.98	1.40	1.31	1.18	1.46	1.02	1.02	1.03	0.95

Mean SI proliferation assay data from donors 26-50 in the non-adjusted data set for peptides 1-42. Numbers in red indicate positive responses (SI ≥2.00, *p* <0.05). Numbers in pink indicate borderline responses (SI ≥1.90, *p* <0.05).

PEPTIDE	26	27	28	29	30	31	32	33	34	35	36	37	38	39	40	41	42	43	44	45	46	47	48	49	50
1	1.11	0.56	0.92	0.86	1.50	1.38	1.10	0.88	0.95	0.93	1.05	1.57	1.16	0.80	1.00	1.23	1.22	0.65	0.65	0.77	0.71	1.04	1.00	1.08	1.30
2	1.25	0.65	0.96	1.07	1.22	0.95	0.99	1.13	1.20	1.09	0.96	1.47	1.35	0.95	1.03	1.41	1.51	0.85	0.65	0.90	0.80	0.95	0.99	1.01	1.33
3	1.24	1.12	0.90	0.89	1.08	1.17	1.08	1.21	1.09	1.00	1.23	1.31	1.30	1.09	1.37	1.18	1.30	1.18	0.56	0.73	0.81	1.17	0.86	0.91	1.46
4	1.25	1.73	0.95	0.82	1.07	1.43	1.23	1.10	1.23	0.89	1.11	1.37	1.17	1.11	1.20	1.40	1.15	1.17	0.48	0.76	1.04	1.01	0.98	1.00	1.53
5	1.46	0.94	1.00	0.78	1.24	1.57	1.40	1.29	1.10	0.98	1.34	1.49	1.20	1.17	1.10	1.36	1.05	0.76	0.86	0.76	0.85	0.93	0.80	0.92	1.44
6	1.30	1.00	1.11	0.87	1.29	0.94	1.20	1.94	1.07	0.83	0.93	1.26	1.16	2.77	1.19	1.18	1.27	0.97	2.77	0.84	0.81	1.11	0.70	1.15	1.37
7	1.27	1.11	1.02	0.77	1.12	0.56	0.51	1.31	1.11	0.95	1.38	1.11	1.22	1.07	1.15	1.23	1.43	2.39	0.90	0.74	0.89	0.83	0.64	0.75	1.29
8	1.12	1.01	0.96	0.76	1.21	1.04	1.04	1.16	0.80	0.88	1.20	1.49	1.11	1.41	0.74	1.23	0.94	1.10	1.15	0.77	0.97	0.84	0.56	1.06	1.09
9	1.53	0.76	0.93	1.19	1.05	1.57	0.72	1.05	0.73	1.06	0.88	1.32	1.38	1.00	1.11	1.52	1.44	0.76	0.38	0.93	0.85	1.16	0.78	0.86	1.19
10	1.17	0.89	0.97	1.51	0.97	1.10	0.72	1.42	1.13	1.05	1.09	1.45	1.12	1.29	1.31	1.72	1.33	0.87	0.63	0.94	0.91	1.13	1.04	1.20	1.21
11	1.26	1.01	1.00	1.17	1.12	1.41	0.88	1.02	0.99	1.10	1.05	1.21	1.17	1.26	1.55	1.39	1.01	0.84	0.60	0.93	0.99	1.25	0.79	1.00	1.20
12	1.31	1.30	0.96	1.16	1.28	1.14	1.08	1.29	1.29	1.08	1.14	1.40	1.12	1.34	1.07	1.51	0.94	0.90	0.44	0.90	1.11	1.31	1.12	1.23	1.46
13	1.42	1.20	0.99	1.28	1.24	1.39	1.13	1.35	0.79	0.99	1.04	1.34	1.24	1.47	1.27	1.50	0.97	0.98	0.62	0.89	1.04	1.06	0.93	1.17	1.78
14	1.21	1.09	0.95	1.52	1.03	1.15	0.80	0.88	1.02	0.96	1.13	1.10	1.37	1.37	1.12	1.28	1.14	1.10	0.72	0.91	0.98	1.14	0.82	1.05	1.20
15	1.25	0.48	1.28	1.19	1.00	1.26	1.29	0.76	0.97	1.37	1.14	1.08	1.01	0.64	0.96	1.41	1.37	0.83	1.53	0.73	0.73	0.72	1.07	1.03	1.25
16	1.23	0.77	1.00	1.32	1.40	0.55	0.56	0.78	1.08	1.46	1.48	0.66	1.36	0.61	1.43	1.26	1.61	1.34	1.59	0.79	0.70	0.64	0.88	0.71	1.15
17	1.59	1.38	1.36	1.46	1.61	0.99	1.28	0.90	1.23	1.72	1.36	1.19	1.48	1.23	1.37	1.49	1.63	1.44	1.54	0.84	0.97	0.81	1.68	1.09	1.32
18	1.38	0.84	1.31	1.30	1.16	1.32	1.63	0.62	1.01	1.32	1.13	1.53	1.21	0.80	1.07	1.15	1.22	0.94	1.24	0.90	0.73	0.85	0.99	0.93	1.06
19	1.61	1.04	1.23	1.24	1.21	1.23	1.53	0.71	0.87	1.22	0.97	1.23	1.12	0.92	1.47	1.23	1.72	1.28	1.27	0.78	0.87	0.63	0.89	1.04	1.05
20	1.51	1.19	1.40	1.27	1.10	1.14	1.27	1.13	0.90	1.23	1.17	0.97	1.21	0.83	1.22	1.19	1.07	1.17	1.33	0.74	0.88	0.80	0.94	1.17	0.88
21	1.41	1.27	1.45	1.44	1.27	0.97	0.99	0.92	0.76	1.32	1.12	1.05	1.21	0.97	1.02	1.09	1.16	1.11	1.15	0.67	1.06	0.63	1.20	0.89	0.94
22	1.26	1.38	1.64	1.40	1.13	1.06	1.22	1.08	0.98	1.23	1.23	1.44	1.25	1.06	0.86	1.05	1.01	1.52	1.35	0.65	0.93	0.66	1.22	0.99	0.69
23	1.12	0.67	1.08	1.16	1.31	1.14	1.12	0.89	1.00	1.40	0.84	1.22	1.06	0.66	0.96	1.30	1.42	0.85	1.36	1.05	1.10	0.90	1.12	0.84	1.51
24	1.24	0.67	1.08	1.17	1.52	1.09	1.04	0.76	1.23	1.22	0.86	0.86	1.02	0.84	0.96	1.21	1.22	1.31	1.62	1.02	0.94	0.83	1.03	0.84	1.38
25	1.37	0.74	1.00	1.17	1.23	1.11	1.03	0.85	1.28	1.28	0.82	1.16	1.05	0.93	1.14	1.22	1.33	0.63	1.14	1.05	1.07	0.90	0.97	1.02	1.04
26	1.42	0.84	1.09	1.19	1.46	1.03	1.00	0.66	1.13	1.12	0.87	0.94	1.05	0.88	1.24	1.25	1.06	0.78	1.52	1.04	0.93	0.93	0.96	1.18	1.29
27	1.32	1.01	1.11	1.07	1.26	1.04	0.95	1.04	1.17	0.99	0.70	0.98	1.00	1.02	1.05	1.41	1.06	0.95	1.31	0.99	0.87	0.90	1.10	1.00	0.90
28	1.25	1.16	1.11	1.10	1.10	1.08	0.89	1.19	0.99	1.00	0.91	0.77	1.01	1.05	1.03	1.09	1.18	0.94	1.04	1.17	1.08	0.89	1.16	1.17	0.92
29	0.80	0.68	1.24	1.41	0.77	0.79	1.52	0.80	0.59	0.91	0.80	0.96	1.12	0.79	0.90	0.99	1.53	1.09	1.08	0.96	0.64	1.20	1.10	1.22	1.11
30	0.97	0.70	1.19	1.49	0.97	0.88	1.44	0.96	0.64	0.98	0.88	0.84	1.36	0.94	1.38	0.90	1.71	1.57	1.71	1.00	0.72	1.37	1.45	1.33	1.98
31	1.01	0.82	1.11	1.15	0.93	0.70	1.17	0.72	0.82	0.77	0.92	0.69	1.17	0.83	0.94	0.96	1.37	1.02	0.93	0.91	0.83	1.02	1.05	1.21	1.27
32	0.93	0.72	0.99	1.23	0.98	0.67	1.59	0.94	0.79	0.89	0.88	0.87	1.07	0.90	1.01	1.01	1.33	0.97	1.09	0.96	0.78	1.02	1.18	1.35	1.19
33	1.04	0.86	1.19	1.38	0.82	0.75	1.37	1.29	0.99	0.90	1.12	0.97	1.11	0.82	0.99	1.20	1.41	0.98	1.15	0.93	0.84	0.97	0.88	1.18	1.26
34	1.01	1.24	1.06	1.44	1.06	0.91	1.42	1.28	0.95	0.86	1.19	1.37	1.04	0.88	1.16	1.03	1.33	1.01	0.90	0.90	0.77	0.93	0.99	1.01	1.46
35	1.00	0.88	1.33	1.28	0.74	0.79	1.57	1.56	0.84	1.02	1.41	1.09	1.07	0.92	0.76	0.91	1.28	1.10	0.95	0.87	0.78	0.87	0.88	1.08	1.24
36	0.97	1.31	1.51	1.14	0.85	0.89	1.27	0.94	1.05	0.97	1.02	1.07	1.15	0.90	0.99	1.11	1.09	1.19	0.96	0.84	0.75	0.95	0.90	1.20	1.18
37	0.75	0.94	1.02	1.56	1.31	0.93	1.23	1.26	0.71	1.02	0.77	1.19	1.01	0.97	0.93	1.03	1.55	1.09	1.47	0.96	0.72	1.25	1.76	0.94	1.20
38	0.86	1.05	0.88	1.27	0.95	0.89	1.23	0.94	0.89	0.88	0.84	0.95	1.10	0.99	0.86	1.00	1.29	0.85	1.13	0.92	0.85	1.06	1.15	0.91	1.18
39	0.91	1.16	0.95	1.12	1.00	0.72	1.29	0.94	0.81	0.95	0.86	0.96	1.01	1.09	1.09	1.03	1.09	1.38	1.39	0.97	0.81	1.03	1.15	0.87	1.26
40	0.93	1.08	0.82	1.35	1.20	0.75	1.29	0.80	0.92	0.88	0.81	0.98	0.96	0.97	0.83	0.96	1.05	1.09	1.83	1.18	0.89	1.15	1.04	1.11	1.17
41	1.00	0.99	0.86	1.07	1.04	0.85	1.17	0.89	0.85	0.89	0.90	1.02	0.94	0.87	1.30	1.24	1.18	1.38	0.96	0.96	0.77	1.20	1.01	1.08	1.10
42	0.88	1.09	0.87	0.93	1.00	0.81	0.96	1.13	0.93	0.92	0.94	0.89	0.73	1.02	1.05	1.21	1.40	1.12	1.15	1.21	0.95	1.04	1.09	1.00	1.16

Mean SI proliferation assay data from donors 1-25 in the non-adjusted data set for peptides 43-80. Numbers in red indicate positive responses (SI ≥2.00, p <0.05). Numbers in pink indicate borderline responses (SI ≥1.90, p <0.05).

PEPTIDE	1	2	3	4	5	6	7	8	9	10	11	12	13	14	15	16	17	18	19	20	21	22	23	24	25
43	0.89	1.04	1.16	1.30	1.41	0.70	1.29	1.22	1.10	1.15	0.71	1.03	0.73	0.85	0.83	0.76	0.78	1.16	1.01	1.07	1.49	0.77	0.58	1.53	1.06
44	1.28	1.12	1.73	1.29	1.20	1.00	1.34	1.57	1.20	1.11	0.79	1.13	0.85	1.11	0.89	1.00	1.00	1.08	0.83	1.22	1.46	1.14	0.70	1.35	1.13
45	1.20	0.96	1.30	1.31	0.93	0.97	1.56	1.69	1.17	1.06	0.82	0.89	0.72	1.16	0.93	0.86	0.90	0.98	0.82	0.96	1.11	1.11	0.83	1.43	0.98
46	1.24	0.98	1.27	1.43	0.88	1.16	1.12	1.44	1.04	0.91	0.78	1.07	0.77	1.09	0.78	0.84	0.90	1.03	0.91	0.86	1.21	0.98	0.89	1.40	1.04
47	1.17	1.03	1.23	1.20	0.99	1.08	1.09	1.18	0.93	0.82	1.07	0.94	0.73	1.07	0.84	0.86	0.95	0.80	1.04	1.02	1.20	1.07	0.69	1.27	1.18
48	1.11	0.98	1.24	1.30	1.14	0.72	0.97	1.38	0.95	0.84	0.83	1.11	0.83	1.00	0.98	1.02	1.00	0.88	1.04	0.89	1.24	1.15	0.68	1.41	0.99
49	1.00	1.18	1.31	1.19	0.83	1.13	0.85	1.15	0.84	0.68	0.92	1.16	0.82	0.88	0.93	1.06	0.94	0.87	1.16	0.79	1.12	1.17	0.84	1.32	1.09
50	0.66	0.97	1.35	1.36	0.82	0.78	0.80	1.00	0.94	0.77	1.09	1.42	0.91	0.92	0.83	1.21	0.83	1.10	0.75	0.62	1.08	1.07	0.79	1.09	1.14
51	0.88	0.93	1.25	1.17	1.42	0.71	1.24	1.56	1.31	1.48	0.64	0.75	0.87	0.95	1.05	1.05	0.85	1.31	0.95	0.93	1.35	0.79	0.86	1.37	1.02
52	1.22	0.86	1.21	1.30	1.25	0.98	1.19	1.02	1.35	1.12	0.66	0.78	0.74	1.02	1.05	1.09	0.92	1.16	1.06	1.13	1.33	0.91	0.87	1.38	1.02
53	1.40	0.92	1.28	1.22	0.80	0.83	1.17	1.41	1.13	0.99	0.76	0.88	0.70	1.13	0.95	0.99	1.06	1.10	1.24	1.10	1.72	0.85	0.90	1.35	1.14
54	1.04	0.88	0.99	1.02	1.17	0.95	1.19	1.30	1.09	1.06	0.86	0.92	0.77	1.20	0.70	0.94	1.00	0.87	0.89	1.22	1.27	0.99	0.86	1.28	1.07
55	1.07	0.87	1.11	1.07	1.11	1.07	1.27	1.01	1.24	1.08	0.78	1.00	0.81	1.00	1.00	1.18	1.15	0.86	0.96	1.24	1.38	1.08	0.91	1.13	1.15
56	1.12	0.86	1.24	1.20	1.01	0.99	1.10	1.03	1.11	0.92	0.81	1.01	0.88	1.15	0.93	0.94	1.27	0.92	1.02	1.14	1.19	0.97	0.81	1.14	1.03
57	0.73	1.01	1.04	0.83	1.25	0.69	0.66	1.41	0.80	0.98	0.67	1.03	0.92	1.13	0.84	0.87	0.63	1.20	0.61	1.52	1.45	0.71	0.74	1.15	0.79
58	1.08	1.13	1.02	0.97	1.12	0.98	0.80	1.16	0.94	0.92	0.76	0.76	0.71	1.35	0.91	1.00	0.76	1.14	0.71	1.34	1.27	1.02	0.89	1.29	0.77
59	0.99	1.07	1.07	0.97	1.06	0.89	0.77	1.27	1.11	0.86	1.01	0.92	0.69	1.34	0.73	0.91	0.98	0.81	0.77	1.57	1.12	1.01	1.00	1.28	0.88
60	0.98	1.40	1.08	0.99	0.89	0.95	0.89	0.98	1.10	0.83	0.91	1.17	0.72	1.26	0.67	0.87	0.92	0.86	0.76	1.57	1.71	0.97	0.80	1.03	0.73
61	0.83	1.26	1.42	1.28	1.05	1.65	0.80	0.97	0.96	0.99	1.17	1.07	0.65	1.54	0.83	0.89	0.85	0.72	0.68	1.36	1.29	0.86	0.86	1.13	0.97
62	0.90	1.36	1.35	1.00	1.03	1.00	0.71	1.07	0.80	1.10	0.94	1.23	0.78	1.22	0.73	0.86	1.10	0.97	1.06	1.36	1.09	0.90	0.93	1.21	1.10
63	0.84	1.08	1.39	1.02	1.10	1.01	0.59	0.88	0.77	1.06	1.03	1.25	0.77	1.11	0.93	0.93	0.89	1.06	0.95	1.22	1.03	0.69	0.86	1.13	0.91
64	0.66	1.02	1.16	1.04	1.19	0.93	0.65	1.07	0.72	1.17	1.09	1.67	0.91	0.91	0.89	1.01	0.73	1.00	0.77	1.23	1.14	0.83	0.70	0.99	0.89
65	0.88	0.91	0.86	0.70	0.76	0.59	0.77	1.34	0.84	1.08	0.58	0.80	1.16	1.03	0.72	0.98	0.82	1.37	0.93	1.51	1.06	0.91	1.03	1.14	0.89
66	1.13	1.05	1.01	0.80	0.93	0.98	0.75	1.06	1.10	0.99	0.82	0.76	1.13	1.25	0.86	0.98	1.04	1.32	0.91	1.48	1.20	1.16	0.95	1.03	1.00
67	1.09	1.15	1.15	0.88	0.95	0.74	0.82	0.86	1.38	0.92	0.71	0.79	0.95	1.16	0.93	1.07	0.98	1.08	0.97	1.36	1.07	1.41	0.90	1.06	0.85
68	1.19	1.09	1.01	0.98	0.94	0.83	0.88	0.84	1.15	0.88	0.79	0.77	1.02	1.12	0.76	0.81	1.12	0.95	0.96	1.69	1.04	1.25	1.06	1.10	0.87
69	0.95	1.17	1.07	0.86	1.00	0.68	0.77	0.77	1.02	1.09	1.34	0.86	1.02	1.10	0.96	0.81	1.00	1.06	1.00	1.39	0.96	1.40	0.86	1.10	0.79
70	1.11	1.12	1.04	1.10	0.83	1.14	0.92	0.75	1.10	1.12	1.40	1.02	0.85	1.21	0.95	1.03	1.15	1.03	1.02	1.05	1.06	1.21	0.91	1.09	0.84
71	0.57	1.76	0.82	1.02	1.25	1.09	0.90	1.61	0.91	0.67	1.38	0.47	1.96	0.85	0.66	1.11	0.86	1.24	1.21	0.92	0.86	0.59	1.06	1.06	2.20
72	0.89	2.69	1.27	1.38	1.43	1.14	1.81	1.46	1.31	0.76	1.11	0.49	1.12	1.11	0.64	1.39	1.05	0.98	1.12	1.18	0.93	0.70	1.27	1.18	2.55
73	0.94	2.46	1.13	1.20	1.25	1.26	1.85	1.35	1.11	0.76	1.05	0.59	0.98	1.15	0.92	2.05	1.04	0.88	0.85	1.07	1.37	0.81	1.05	1.32	1.48
74	1.03	1.86	1.26	1.20	1.26	1.33	1.66	1.24	1.30	0.75	1.42	0.80	0.93	0.92	0.68	0.93	1.24	0.84	0.64	1.04	0.89	0.82	1.20	1.42	1.16
75	2.67	2.07	0.98	1.14	1.47	1.36	1.44	1.02	1.38	0.80	1.42	1.01	0.83	0.93	0.71	1.00	1.28	0.70	0.78	1.16	0.89	0.94	1.35	1.38	1.11
76	0.79	1.84	1.15	0.97	1.95	1.31	1.64	0.90	1.19	0.91	1.28	1.21	1.09	1.01	0.73	0.92	0.94	0.84	0.73	0.85	0.94	0.74	1.37	1.39	1.11
77	0.99	1.72	1.08	0.96	1.85	1.53	1.23	0.90	1.09	0.88	1.50	2.72	1.02	0.98	0.89	0.96	1.00	0.84	0.67	1.09	0.91	0.81	1.27	1.42	1.28
78	0.78	1.14	1.17	0.96	1.61	1.22	1.03	0.90	0.98	0.72	1.69	3.59	0.89	0.94	0.59	0.76	0.82	0.86	0.74	0.86	0.86	0.89	1.91	1.35	1.49
79	0.82	1.27	1.05	1.04	1.33	0.92	0.87	1.64	1.03	0.82	0.96	0.54	1.52	1.04	0.86	1.05	0.76	1.31	1.37	0.97	0.92	0.76	1.31	0.97	1.26
80	1.18	1.67	1.12	1.01	1.88	1.21	1.20	1.43	1.17	0.96	1.20	0.52	1.32	1.22	1.04	1.05	1.14	1.06	1.08	1.03	1.02	0.87	1.13	1.15	1.31
C3	0.98	2.65	1.28	3.11	1.29	1.07	1.15	1.30	0.93	0.89	0.99	0.64	4.36	3.50	3.05	1.08	13.12	0.96	3.93	1.14	1.18	0.83	1.03	1.55	2.33
C32	1.04	2.03	1.69	0.98	1.37	1.11	1.28	1.63	1.09	0.95	1.10	0.78	1.06	1.01	1.98	1.21	1.15	2.30	1.33	1.28	1.45	0.82	1.17	1.08	2.06
KLH	2.27	11.11	10.98	4.71	3.09	7.57	2.26	2.57	2.74	1.06	3.97	2.34	6.66	2.98	12.65	10.18	5.00	3.30	3.83	2.14	10.50	5.34	6.18	2.77	3.51

Mean SI proliferation assay data from donors 26-50 in the non-adjusted data set for peptides 43-80. Numbers in red indicate positive responses (SI ≥2.00, *p* <0.05). Numbers in pink indicate borderline responses (SI ≥1.90, *p* <0.05).

PEPTIDE	26	27	28	29	30	31	32	33	34	35	36	37	38	39	40	41	42	43	44	45	46	47	48	49	50
43	1.06	0.51	0.78	1.05	2.69	0.82	1.33	0.86	0.68	1.18	0.66	1.07	1.12	0.94	0.98	1.33	0.84	0.78	0.76	0.81	0.72	0.82	1.07	1.04	1.00
44	1.29	0.59	0.81	1.22	1.59	0.74	1.16	0.83	0.77	1.22	0.82	1.19	1.38	0.88	0.91	1.18	0.96	0.95	1.14	0.73	0.94	0.90	0.90	0.89	1.27
45	1.27	0.62	0.76	1.23	2.07	0.89	1.36	0.84	0.88	1.19	0.85	1.05	1.21	0.79	0.75	1.14	0.85	1.05	1.02	0.80	1.04	0.99	0.79	0.82	1.23
46	1.01	0.69	0.70	1.12	1.40	0.91	1.00	0.98	0.95	1.14	0.93	0.96	1.06	0.99	0.66	1.12	0.92	0.77	1.07	0.84	1.09	1.00	0.91	1.06	0.92
47	1.11	0.74	0.75	1.04	1.18	0.90	0.88	0.97	0.69	1.27	0.91	1.15	1.20	0.97	0.86	1.50	0.78	0.62	1.24	0.88	0.93	0.81	0.91	0.89	0.89
48	0.90	0.70	0.82	0.94	1.06	0.79	1.18	0.91	0.78	1.23	0.96	1.06	1.20	0.98	0.87	1.57	0.86	0.70	1.54	0.90	1.16	1.00	0.96	0.82	1.05
49	0.85	0.73	0.86	0.96	1.04	0.99	1.02	0.86	0.78	1.13	1.09	1.19	1.31	0.97	0.80	1.68	0.83	0.95	0.94	0.96	0.89	0.84	1.05	0.98	1.03
50	0.84	0.78	0.94	0.98	1.12	1.06	1.13	1.02	0.87	1.12	1.24	0.99	1.26	1.29	0.80	1.79	0.94	0.84	1.08	0.69	0.99	0.84	1.14	0.65	0.88
51	1.01	0.50	0.99	1.05	1.41	0.91	1.31	0.74	0.81	1.06	0.57	1.09	1.22	1.00	0.88	1.05	0.90	0.98	0.97	0.93	0.89	0.96	1.02	1.11	0.99
52	1.21	0.67	0.83	1.21	1.07	0.97	1.24	0.69	0.80	1.02	0.56	1.01	1.27	0.91	0.86	1.05	1.02	0.92	0.88	0.99	0.80	0.82	1.00	0.92	0.92
53	1.01	0.68	0.89	1.17	1.30	0.87	0.93	0.78	0.81	1.00	0.76	0.92	1.18	0.89	0.81	1.10	1.17	0.93	1.60	1.01	1.01	0.84	1.17	0.88	1.02
54	1.06	0.76	0.89	1.20	1.28	1.00	1.15	0.80	0.89	0.90	0.71	0.96	1.00	0.88	0.86	0.99	0.99	0.70	1.22	1.06	1.02	0.85	0.95	1.01	0.92
55	1.23	0.65	0.74	0.95	1.27	0.82	0.70	1.03	0.81	0.98	0.75	0.92	0.96	0.93	0.89	1.36	0.93	0.99	1.45	1.03	0.99	1.06	0.84	0.89	0.79
56	1.03	0.79	0.86	1.18	1.11	0.97	0.99	0.82	0.88	1.02	1.02	0.91	0.88	1.12	0.79	1.20	0.93	0.83	1.19	1.11	1.01	1.02	1.00	0.80	0.98
57	1.36	0.51	1.23	0.99	1.34	1.32	0.91	1.10	0.86	1.06	0.85	0.97	1.00	1.22	1.21	1.29	1.23	1.44	0.59	0.76	1.23	1.05	1.27	0.81	1.14
58	1.38	0.68	1.18	1.05	1.17	1.37	1.12	1.01	1.04	1.18	1.08	0.95	1.14	1.31	1.26	1.21	1.04	1.37	0.86	0.79	1.01	1.11	1.05	1.13	0.94
59	1.51	0.72	1.10	1.11	1.32	1.22	1.37	0.81	0.88	1.17	0.82	1.06	1.14	1.35	1.13	1.15	1.19	1.00	0.56	0.85	1.10	1.04	0.81	0.86	1.11
60	1.39	0.68	0.98	1.07	1.16	1.09	1.24	0.94	0.91	1.02	1.03	1.28	0.94	1.20	1.19	0.89	1.09	1.24	0.54	0.88	1.25	1.01	0.79	0.68	0.90
61	1.41	0.87	0.93	1.14	1.03	1.08	1.28	1.15	0.88	1.11	1.26	1.34	1.07	1.18	1.20	1.34	0.91	0.91	0.47	0.68	1.24	1.16	0.87	0.85	0.83
62	1.35	1.02	1.14	1.14	0.82	1.31	1.19	1.13	0.93	1.03	1.21	1.03	1.10	1.43	1.14	0.95	1.00	1.08	0.72	0.72	1.24	0.84	0.79	0.76	0.78
63	1.29	0.87	1.10	1.10	0.93	1.36	1.29	1.01	0.96	1.00	1.21	1.15	1.19	1.17	1.26	1.26	1.08	1.13	0.96	0.80	1.15	1.06	0.67	1.00	0.66
64	1.12	1.00	1.05	1.21	1.13	1.69	1.04	0.92	0.76	1.03	0.93	1.28	1.38	1.18	0.90	1.08	1.05	0.88	1.24	0.81	1.03	1.16	0.90	0.80	0.60
65	1.33	0.47	1.03	0.84	0.97	1.07	0.83	1.08	0.80	1.28	0.85	1.25	0.97	1.13	1.12	1.46	1.05	1.57	0.59	0.93	0.96	0.98	1.25	1.08	1.14
66	1.42	0.51	1.12	1.01	1.19	1.23	0.92	0.91	1.10	1.42	0.96	1.07	1.26	0.89	1.08	1.53	1.19	1.39	0.57	0.94	1.26	0.91	1.10	1.19	1.05
67	1.21	0.70	0.93	0.89	1.38	1.42	0.90	0.65	0.79	1.19	0.69	1.09	0.84	1.15	1.27	1.16	1.10	1.06	0.64	0.93	1.28	0.80	1.23	0.98	1.14
68	1.20	0.96	0.84	1.12	0.99	1.00	1.15	1.13	1.16	1.18	0.82	0.88	0.87	1.13	1.02	0.90	1.04	1.09	0.62	0.88	1.11	0.95	1.12	0.97	1.10
69	1.18	0.90	0.76	1.07	1.02	1.06	1.11	0.82	1.07	0.98	0.91	1.14	0.88	1.08	1.14	1.07	1.10	0.99	0.48	0.99	0.98	0.88	0.94	1.05	0.89
70	1.07	0.82	0.71	1.10	0.88	0.99	1.07	0.84	0.90	0.96	1.01	1.24	0.85	1.09	0.95	0.93	1.04	1.02	0.58	0.82	1.08	0.70	1.14	0.77	0.97
71	1.11	0.62	0.82	0.93	1.01	0.78	0.74	4.80	0.89	0.95	0.79	1.21	0.74	0.49	1.01	1.07	1.88	1.02	3.96	0.87	0.77	0.87	0.88	0.91	1.46
72	1.24	0.54	1.06	1.50	1.29	0.87	0.86	4.89	1.17	1.19	0.65	1.09	0.76	0.80	0.83	1.25	1.82	1.17	10.79	0.94	0.97	1.09	0.84	0.90	1.16
73	1.18	0.61	1.03	1.22	1.11	0.80	0.76	1.57	1.18	1.40	0.71	0.98	0.89	0.64	0.98	0.90	2.14	1.09	0.79	0.86	1.07	1.16	0.88	0.91	1.65
74	1.19	0.73	0.93	0.98	1.06	0.69	1.15	1.61	0.80	1.52	0.84	1.16	0.85	0.77	0.73	1.08	1.74	1.19	1.16	1.15	0.95	1.00	0.70	0.91	1.54
75	1.33	1.01	1.01	1.04	1.11	0.69	0.99	1.65	1.05	1.16	0.96	0.93	0.87	0.68	0.81	1.08	1.48	1.23	0.91	0.93	0.94	0.96	0.90	0.83	1.22
76	1.06	0.99	0.87	1.20	1.15	0.64	0.93	3.65	0.92	1.39	0.79	1.00	0.91	0.81	0.62	0.96	1.19	0.92	1.27	0.85	0.99	0.82	0.73	0.83	1.18
77	1.36	0.89	1.00	0.80	1.17	0.83	1.05	1.30	0.66	1.49	0.91	1.08	0.91	0.86	0.54	0.82	1.20	0.79	0.81	0.80	0.87	0.71	0.64	0.77	0.98
78	1.02	1.00	0.89	0.88	1.56	0.83	1.06	2.22	0.79	1.16	1.38	1.27	1.02	2.70	0.65	0.77	1.21	0.84	1.03	0.86	0.83	0.53	0.77	0.92	0.65
79	1.14	0.90	1.00	1.34	1.20	1.30	0.89	1.49	1.33	0.98	0.72	0.94	0.97	0.55	0.98	1.50	2.32	1.13	1.27	0.86	1.06	1.59	1.81	1.05	1.71
80	1.05	0.76	1.15	1.38	1.19	1.33	0.73	0.98	1.33	0.89	0.72	0.98	1.06	0.70	0.89	1.42	2.03	1.29	1.05	0.82	1.11	1.10	1.06	0.98	1.69
C3	1.23	0.96	1.05	1.38	14.32	0.97	0.78	1.28	1.37	1.08	0.69	1.04	2.38	1.42	0.92	1.26	1.46	3.60	1.29	1.10	1.09	1.17	1.11	0.82	1.30
C32	1.25	1.47	0.98	1.06	1.18	1.29	5.57	1.69	1.28	1.19	1.70	0.92	1.17	0.84	0.87	1.28	1.46	1.34	1.43	0.86	1.05	7.18	1.27	0.66	1.24
KLH	2.52	9.53	2.43	5.96	7.87	1.38	1.33	9.94	8.32	3.49	4.04	1.91	5.17	3.45	5.14	3.47	6.72	12.55	7.97	11.78	2.71	3.60	14.07	0.62	5.43

8. Appendix II

Mean SI proliferation assay data from donors 1-25 in the adjusted data set for peptides 1-42. Numbers in red indicate positive responses (SI ≥2.00, *p* <0.05). Numbers in pink indicate borderline responses (SI ≥1.90, *p* <0.05).

PEPTIDE	1	2	3	4	5	6	7	8	9	10	11	12	13	14	15	16	17	18	19	20	21	22	23	24	25
1	1.02	0.87	0.96	1.32	1.20	0.59	1.70	1.39	1.48	1.77	0.93	1.17	0.98	1.26	0.92	1.92	0.87	1.10	1.21	0.96	1.00	1.37	0.71	0.83	1.18
2	1.49	0.96	1.19	1.45	1.21	1.02	1.62	1.01	1.91	1.49	1.09	1.03	1.03	1.22	1.00	1.52	0.99	1.07	1.29	0.92	1.23	1.15	0.88	1.11	1.14
3	1.39	0.99	1.06	1.19	0.93	1.11	1.48	1.13	1.67	1.59	0.97	0.87	0.81	1.34	1.12	0.94	1.10	1.03	1.45	1.22	1.30	3.52	0.84	1.05	1.20
4	1.09	0.93	1.01	1.51	0.97	1.07	1.53	1.03	1.90	1.63	1.29	1.25	0.86	1.39	1.03	0.83	0.90	0.92	1.20	1.08	1.63	3.88	0.92	1.19	1.08
5	1.28	0.77	0.21	1.38	1.26	1.48	1.51	0.99	1.63	1.37	1.36	0.90	0.75	1.23	1.10	0.85	0.92	1.00	1.11	1.00	1.87	2.04	0.81	1.20	1.22
6	1.14	0.94	0.26	1.20	1.07	1.25	1.55	1.02	1.91	1.03	1.01	1.02	0.84	1.36	1.19	1.14	1.05	0.91	0.90	0.72	1.48	1.16	1.07	1.15	1.27
7	1.10	1.15	0.35	1.44	1.21	1.57	1.41	0.86	1.04	0.98	1.08	1.12	0.96	0.96	0.99	0.94	0.77	0.68	1.02	0.66	2.08	1.38	0.99	1.15	1.18
8	0.66	1.09	0.22	1.41	1.52	1.43	0.95	0.91	1.61	1.00	0.97	1.21	1.18	1.00	0.97	1.19	0.78	0.83	1.37	0.78	1.59	1.15	1.02	1.01	1.19
9	1.12	0.78	1.09	1.48	1.38	0.66	1.74	1.40	1.25	1.60	0.85	1.25	0.95	1.40	1.11	1.30	0.91	1.19	0.92	1.12	1.41	1.53	0.79	1.03	1.12
10	1.55	1.13	1.22	1.46	1.04	0.91	1.52	1.18	1.17	1.73	0.92	1.12	0.96	1.18	1.10	1.09	0.92	1.06	1.00	0.96	1.29	1.49	0.80	1.17	1.13
11	1.18	1.06	1.22	1.26	1.15	0.97	1.68	1.09	1.09	2.45	0.92	0.91	0.88	1.21	1.16	1.02	0.96	1.30	0.92	1.18	1.69	1.37	0.76	1.24	1.06
12	1.90	0.90	0.95	1.27	1.30	0.97	1.37	1.10	1.07	1.68	1.00	1.09	0.93	1.18	0.90	0.93	1.17	1.16	1.17	1.37	1.21	1.34	0.85	1.22	1.18
13	1.21	1.20	0.24	1.40	1.29	1.04	1.43	1.11	1.01	1.27	1.38	0.89	0.92	1.63	1.23	1.20	1.19	1.02	0.96	0.88	1.13	1.87	0.99	1.43	1.08
14	1.22	1.28	0.23	1.49	1.17	1.10	1.59	0.98	1.19	1.03	1.50	1.07	0.95	1.28	1.20	1.22	1.12	1.00	1.46	1.08	1.03	1.28	0.87	1.17	1.13
15	1.05	0.98	0.76	0.84	1.51	0.74	1.22	1.42	1.25	1.24	1.65	0.60	0.61	1.01	0.88	0.75	0.88	1.05	1.16	1.73	0.92	0.94	1.13	0.82	0.79
16	1.12	1.10	0.89	0.89	1.17	1.35	1.90	1.27	0.79	1.14	2.09	0.60	0.60	0.96	1.19	0.81	0.89	0.74	1.25	1.33	1.18	6.45	1.21	1.05	0.93
17	1.39	0.93	1.03	0.81	1.28	1.15	1.27	1.37	1.40	1.05	1.43	0.62	0.70	1.35	1.13	1.03	1.29	1.05	1.37	1.96	0.91	3.17	1.17	0.96	1.05
18	1.19	0.88	0.88	1.20	0.88	1.08	1.47	1.25	1.20	0.87	1.48	0.69	0.59	1.02	1.20	0.84	1.37	0.79	1.15	1.88	0.99	0.84	1.28	0.93	1.03
19	1.04	0.74	0.87	1.24	1.09	1.21	1.10	1.15	1.15	0.86	1.52	0.82	0.68	1.03	1.00	0.94	1.36	1.01	0.75	1.93	0.97	0.83	1.35	0.96	1.33
20	1.21	0.99	0.90	1.21	0.88	1.32	0.94	1.05	1.38	0.99	1.58	0.89	0.69	1.18	1.32	1.06	1.04	0.93	1.11	1.62	0.86	0.88	1.22	1.02	1.05
21	1.12	0.97	1.00	1.06	1.20	1.18	1.04	1.07	1.38	0.83	1.48	0.91	0.78	0.98	0.93	0.95	1.29	0.87	1.02	1.62	0.64	0.97	1.22	0.81	1.19
22	0.76	0.98	0.66	1.10	1.09	1.35	0.98	1.36	1.05	0.72	1.34	0.99	0.75	1.02	0.80	1.14	1.04	0.94	1.21	1.63	0.99	0.86	1.11	0.89	1.26
23	0.91	0.90	0.86	0.73	1.23	0.90	1.08	1.44	1.44	1.30	1.24	0.59	0.79	0.91	0.95	0.93	0.88	1.15	1.13	2.37	1.14	0.96	1.22	1.30	0.90
24	0.99	1.25	1.06	0.76	1.14	0.89	1.27	1.40	1.29	0.92	1.22	0.78	0.84	0.97	1.07	1.10	1.27	1.08	1.00	2.45	1.05	0.78	1.08	1.33	0.81
25	0.98	0.93	1.14	0.90	0.89	0.83	1.05	1.16	1.04	0.95	1.25	0.72	1.02	1.15	1.03	1.07	1.11	0.94	1.02	1.76	1.17	1.16	1.45	1.09	1.10
26	1.03	0.86	0.91	0.90	1.01	0.99	1.06	1.00	1.10	0.86	1.41	0.74	0.75	1.06	1.20	1.13	1.19	1.09	0.89	1.86	1.13	0.87	1.29	1.23	1.11
27	0.79	0.85	0.82	0.91	1.12	1.01	1.01	1.01	1.26	0.77	1.17	0.79	0.81	1.14	1.05	0.99	1.16	0.79	1.24	1.45	1.05	0.97	1.04	1.23	0.88
28	0.89	0.98	0.83	0.98	0.98	1.02	1.10	1.23	1.09	0.81	1.21	0.73	1.02	0.95	0.99	1.14	1.09	0.97	1.11	1.20	0.94	0.92	0.99	1.22	1.05
29	1.17	1.09	0.91	0.82	1.23	0.70	2.08	2.02	1.34	1.47	0.62	1.02	1.04	0.90	0.62	0.83	0.86	0.85	1.06	1.31	0.94	0.88	1.35	0.88	0.63
30	1.09	1.18	0.89	0.80	1.03	0.99	1.97	1.75	1.21	1.60	0.81	0.97	1.15	1.00	0.75	0.80	0.94	0.85	1.66	1.14	1.51	1.55	1.14	1.36	0.82
31	1.25	1.23	0.79	0.80	1.12	0.82	1.90	1.75	1.07	1.28	0.77	0.89	1.03	1.04	0.65	0.81	1.01	0.87	1.22	1.22	1.03	1.14	1.33	1.11	0.67
32	1.12	1.22	0.82	0.88	0.97	1.22	1.59	1.42	1.06	1.13	0.64	1.14	1.08	0.89	0.77	1.29	1.01	0.76	1.14	1.11	0.98	0.89	1.12	1.12	0.71
33	1.07	0.99	0.86	0.94	1.11	1.32	1.65	1.52	0.93	1.30	0.78	1.01	0.99	0.96	0.60	0.96	0.85	0.70	1.44	1.14	1.14	0.93	1.13	1.25	0.84
34	1.15	1.15	0.92	0.91	0.94	1.99	1.68	1.21	0.96	1.19	0.82	1.07	1.14	1.02	0.71	0.98	0.84	0.76	1.32	0.93	1.12	0.91	1.05	1.25	0.73
35	1.36	1.27	0.91	0.96	1.14	1.34	1.45	1.19	0.83	1.15	0.76	1.02	1.03	1.00	0.66	1.18	0.81	0.97	1.39	1.13	1.16	1.01	1.06	1.14	0.87
36	0.83	1.09	0.82	1.02	1.15	0.94	1.18	1.31	0.77	1.09	0.76	1.17	1.08	0.95	0.73	0.98	0.60	0.85	1.22	1.02	0.99	0.95	1.09	1.17	0.99
37	1.26	1.05	0.67	0.79	1.02	0.73	1.74	1.84	1.70	1.67	0.69	0.92	0.98	1.02	0.78	1.00	0.98	1.24	1.26	1.62	1.52	0.96	1.43	1.03	0.97
38	1.24	0.94	0.90	0.92	1.07	0.95	1.44	1.51	1.45	1.10	0.59	0.86	0.89	0.99	0.84	1.08	1.12	1.18	1.25	1.39	1.29	1.20	1.00	1.09	1.01
39	1.26	1.27	1.04	0.99	1.07	0.84	1.23	1.43	1.16	1.03	0.68	0.96	1.03	0.93	0.95	1.00	1.10	1.16	1.16	1.05	1.42	0.99	1.13	0.98	1.03
40	1.23	1.10	0.73	0.91	0.96	1.03	1.26	1.30	1.17	1.20	0.78	0.79	0.93	1.07	0.82	1.19	1.27	1.10	1.15	1.08	1.45	1.04	0.99	1.04	0.88
41	1.16	1.26	0.79	1.06	0.95	1.25	1.23	1.36	1.03	1.05	0.86	0.93	0.92	1.11	0.92	1.12	1.09	1.01	1.43	1.16	1.38	1.20	1.15	1.28	0.81
42	1.19	1.17	0.85	1.00	0.99	1.25	1.18	1.18	1.19	0.81	0.79	1.07	0.96	0.96	1.01	1.19	1.03	1.31	1.25	1.09	1.46	0.98	1.13	1.10	0.99

Mean SI proliferation assay data from donors 26-50 in the adjusted data set for peptides 1-42. Numbers in red indicate positive responses (SI ≥2.00, p <0.05). Numbers in pink indicate borderline responses (SI ≥1.90, p <0.05).

PEPTIDE	26	27	28	29	30	31	32	33	34	35	36	37	38	39	40	41	42	43	44	45	46	47	48	49	50
1	1.09	0.48	0.93	0.86	1.48	1.26	0.98	0.80	0.92	0.94	1.05	1.40	1.14	0.78	0.97	1.15	1.26	0.60	0.73	0.77	0.67	1.04	1.14	0.98	1.37
2	1.28	0.64	0.93	1.06	1.19	0.91	0.99	1.14	1.14	1.05	0.88	1.37	1.36	0.95	1.00	1.32	1.52	0.83	0.66	0.77	0.80	0.94	0.96	1.02	1.32
3	1.26	0.91	0.89	0.93	1.03	1.16	1.06	1.14	1.12	0.98	1.11	1.26	1.34	1.11	1.32	1.15	1.34	1.05	0.57	0.73	0.80	1.25	0.93	0.87	1.40
4	1.25	1.79	0.92	0.81	1.07	1.36	1.17	1.11	1.18	0.89	1.10	1.25	1.20	1.05	1.20	1.36	1.18	1.10	0.63	0.69	1.02	0.96	1.05	0.89	1.39
5	1.47	0.90	0.96	0.78	1.25	1.45	1.40	1.18	1.16	0.97	1.26	1.36	1.22	1.25	1.06	1.27	1.09	1.80	1.12	0.74	0.82	1.04	0.89	0.88	1.42
6	1.24	0.92	1.09	0.87	1.29	0.90	1.16	1.93	0.88	0.85	0.93	1.17	1.21	3.79	1.18	1.13	1.31	2.22	2.98	0.81	0.80	1.06	0.81	1.15	1.44
7	1.28	1.03	0.95	0.75	1.14	0.53	0.55	1.18	1.09	0.95	1.39	0.98	1.26	1.15	1.20	1.16	1.40	2.51	1.04	0.76	0.88	0.79	0.68	0.72	1.17
8	1.09	0.98	1.04	0.74	1.13	0.93	1.06	1.07	0.76	0.88	1.21	1.37	1.13	1.57	0.73	1.14	0.97	1.63	1.28	0.82	0.94	0.83	0.56	1.10	1.10
9	1.50	0.72	0.98	1.18	1.01	1.53	0.61	0.96	0.69	1.08	0.86	1.20	1.41	0.98	1.08	1.57	1.51	0.76	0.36	0.94	0.84	1.10	0.91	0.82	1.20
10	1.18	0.73	0.94	1.44	0.94	1.07	0.71	1.15	1.16	1.04	1.14	1.33	1.13	1.29	1.34	1.57	1.24	0.82	0.85	1.02	0.90	1.12	1.23	1.09	1.19
11	1.25	0.90	0.89	1.16	1.09	1.37	0.86	1.02	0.91	1.08	1.05	1.09	1.19	1.24	1.54	1.35	1.02	0.78	0.58	0.85	0.97	1.08	0.86	0.91	1.25
12	1.33	1.23	0.94	1.17	1.28	1.04	1.09	1.31	1.27	1.09	1.10	1.31	1.11	1.34	1.06	1.41	0.91	0.77	0.55	0.91	1.08	1.22	1.38	1.20	1.55
13	1.46	1.26	0.99	1.27	1.17	1.35	1.09	1.33	0.70	1.01	1.06	1.13	1.22	1.47	1.35	1.40	0.95	0.92	0.77	0.89	1.03	1.06	1.04	1.06	1.78
14	1.15	1.55	0.98	1.49	1.00	1.08	0.77	0.96	0.94	0.98	1.08	1.00	1.32	1.38	1.11	1.22	1.11	1.05	0.76	0.94	0.96	1.13	0.94	1.07	1.19
15	1.18	0.52	1.32	1.23	0.91	1.27	1.19	0.77	0.96	1.42	1.20	1.10	1.04	0.62	0.99	1.42	1.46	0.84	1.49	0.75	0.74	0.72	0.98	1.14	1.21
16	1.19	0.84	0.98	1.36	1.28	0.56	0.54	0.80	1.08	1.51	1.50	0.69	1.37	0.59	1.50	1.24	1.48	1.29	1.45	0.77	0.72	0.68	0.85	0.70	1.12
17	1.53	1.45	1.32	1.48	1.46	1.01	1.24	1.00	1.20	1.84	1.45	1.21	1.50	1.18	1.35	1.46	1.52	1.30	1.55	0.83	1.04	0.81	1.62	1.11	1.28
18	1.33	0.95	1.34	1.30	1.04	1.21	1.51	0.64	0.97	1.37	1.12	1.36	1.20	0.78	1.05	1.23	1.13	0.89	1.22	0.78	0.82	0.87	1.05	0.98	0.98
19	1.57	1.09	1.24	1.30	1.19	1.15	1.45	0.72	0.87	1.28	0.97	1.25	1.08	0.88	1.49	1.20	1.36	1.81	1.25	0.76	0.82	0.65	0.86	1.04	1.07
20	1.41	1.23	1.40	1.31	1.07	1.12	1.23	1.17	0.88	1.27	1.22	1.00	1.22	0.81	1.22	1.12	1.12	1.17	1.18	0.76	0.91	1.20	0.91	1.18	0.83
21	1.35	1.28	1.47	1.46	1.12	1.02	0.97	0.99	0.75	1.40	1.18	0.99	1.26	0.93	1.04	1.08	1.14	1.15	1.09	0.65	1.04	0.63	1.16	0.92	0.88
22	1.19	1.48	1.66	1.40	1.06	1.14	1.09	1.07	0.99	1.28	1.29	1.46	1.25	1.02	0.88	1.07	1.03	1.73	1.19	0.65	0.96	0.67	1.04	1.02	0.66
23	1.07	0.74	1.13	1.18	1.17	1.16	0.97	0.97	1.05	1.30	0.86	1.22	1.08	0.63	1.00	1.24	1.45	0.80	1.42	0.98	1.04	0.91	1.11	0.91	1.25
24	1.13	0.73	1.11	1.19	1.39	1.09	0.91	0.84	1.30	1.27	0.94	0.89	1.02	0.79	0.98	1.21	1.20	1.27	1.37	1.01	0.98	0.86	1.00	0.98	1.24
25	1.29	0.74	1.04	1.18	1.09	1.12	1.00	0.85	1.35	1.35	0.87	1.18	1.07	0.87	1.17	1.20	1.29	0.62	1.18	1.06	1.11	0.89	1.02	1.05	1.04
26	1.31	0.92	1.10	1.23	1.25	1.02	0.99	0.68	1.09	1.20	0.91	0.90	1.07	0.85	1.18	1.24	1.04	0.83	1.58	1.02	0.89	0.99	0.90	1.21	1.12
27	1.24	0.98	1.14	1.10	1.15	1.01	0.96	1.18	1.01	1.03	0.70	0.95	1.02	0.91	1.05	1.40	0.93	0.92	1.28	0.98	0.85	0.93	1.08	0.99	0.87
28	1.19	1.17	1.12	1.14	1.04	1.04	0.82	1.35	1.02	0.96	0.93	0.74	1.01	1.02	1.05	1.08	1.20	0.84	1.01	1.19	1.05	0.93	1.10	1.20	0.89
29	0.79	0.70	1.26	1.35	0.80	0.76	1.51	0.85	0.60	0.90	0.85	0.90	1.06	0.81	0.96	0.98	1.59	1.07	1.06	1.00	0.64	1.04	0.98	1.18	1.11
30	0.95	0.70	1.18	1.33	1.00	0.90	1.47	0.99	0.62	1.03	0.96	0.80	1.33	0.96	1.42	0.88	1.84	1.39	1.77	1.04	0.76	1.25	1.45	1.20	1.97
31	0.96	0.84	1.11	1.14	0.90	0.73	1.19	0.81	0.77	0.81	0.97	0.66	1.14	0.85	1.00	0.94	1.39	0.95	0.84	0.96	0.80	1.05	1.02	1.21	1.25
32	0.92	0.78	1.09	1.18	0.93	0.67	1.37	0.93	0.77	0.87	0.97	0.83	1.04	0.93	1.10	1.04	1.32	0.87	1.05	0.92	0.78	1.02	1.19	1.19	1.25
33	1.01	0.91	1.21	1.30	0.83	0.76	1.34	1.47	0.96	0.89	1.25	0.94	1.07	0.77	1.04	1.26	1.34	0.96	1.09	0.93	0.82	1.00	0.77	1.13	1.27
34	0.97	1.31	1.12	1.33	1.07	0.89	1.42	1.25	0.92	0.87	1.28	1.29	1.03	0.89	1.26	1.02	1.38	0.94	0.78	0.91	0.77	0.94	0.80	1.02	1.57
35	1.00	1.03	1.34	1.22	0.76	0.81	1.54	1.39	0.85	1.04	1.48	1.01	0.99	0.93	0.83	0.91	1.25	0.92	0.91	0.89	0.74	0.90	0.76	1.02	1.27
36	0.93	1.28	1.58	1.16	0.82	0.90	1.33	0.91	1.04	0.99	1.14	1.05	1.12	0.90	1.05	1.10	1.08	1.08	0.91	0.86	0.80	0.90	0.79	1.17	1.18
37	0.73	1.03	1.03	1.64	1.20	0.95	1.18	1.23	0.71	1.03	0.82	1.07	0.94	0.99	1.03	1.00	1.52	1.12	1.36	0.98	0.70	1.25	1.53	0.96	1.14
38	0.87	1.12	0.91	1.18	0.96	0.88	1.26	0.93	0.86	0.86	0.88	0.85	1.06	0.98	0.92	0.99	1.34	0.82	1.15	0.94	0.80	1.11	1.10	0.84	1.11
39	0.91	1.23	0.94	1.05	1.00	0.74	1.34	0.89	0.81	0.94	0.91	0.90	0.94	1.04	1.12	1.05	1.13	1.13	1.35	0.94	0.77	1.07	1.07	0.85	1.24
40	0.90	1.08	0.91	1.24	1.04	0.75	1.26	0.83	0.84	0.91	0.86	0.96	0.94	0.92	0.89	1.02	1.07	0.96	1.78	1.10	0.86	1.19	0.96	1.08	1.12
41	0.97	1.05	0.89	1.01	1.02	0.81	1.19	0.96	0.84	0.90	1.00	0.99	0.90	0.83	1.33	1.20	1.22	1.29	1.01	0.98	0.76	1.21	0.81	1.07	1.09
42	0.86	1.13	0.90	0.90	1.03	0.78	1.02	1.17	0.89	0.89	1.02	0.85	0.72	1.02	1.09	1.21	1.32	1.02	1.13	1.19	0.95	1.03	0.98	0.98	1.06

Mean SI proliferation assay data from donors 1-25 in the adjusted data set for peptides 43-80. Numbers in red indicate positive responses (SI ≥2.00, p <0.05). Numbers in pink indicate borderline responses (SI ≥1.90, p <0.05).

PEPTIDE	1	2	3	4	5	6	7	8	9	10	11	12	13	14	15	16	17	18	19	20	21	22	23	24	25
43	0.94	1.07	1.16	1.32	1.40	0.80	1.27	1.30	1.06	1.10	0.77	0.92	0.75	0.87	0.86	0.90	0.80	1.15	0.85	1.08	1.43	0.74	0.52	1.63	1.06
44	1.41	1.13	1.65	1.29	1.23	1.15	1.29	1.60	1.17	1.01	0.84	1.05	0.77	1.15	0.90	1.08	1.02	1.08	0.79	1.24	1.47	1.08	0.70	1.34	1.15
45	1.28	0.94	1.21	1.25	0.95	1.11	1.48	1.74	1.14	1.00	0.83	0.86	0.71	1.13	0.96	0.98	0.90	0.97	0.76	0.91	1.09	1.08	0.76	1.42	1.01
46	1.28	0.96	1.20	1.32	0.90	1.30	1.09	1.57	1.03	0.88	0.80	1.03	0.75	1.14	0.83	0.92	0.89	1.02	0.85	0.88	1.16	0.94	0.83	1.42	1.07
47	1.27	1.02	1.15	1.12	1.00	1.67	1.16	1.97	0.88	0.84	1.02	0.91	0.70	1.08	0.84	0.98	0.99	0.80	0.91	1.01	1.19	1.11	0.67	1.31	1.20
48	1.19	0.95	1.23	1.18	1.20	0.92	1.06	1.37	0.95	0.83	0.94	1.09	0.78	0.99	1.02	1.10	1.02	0.86	0.88	0.90	1.20	1.00	0.64	1.37	1.00
49	1.03	1.01	1.12	1.10	0.87	1.44	0.96	1.14	0.82	0.72	1.04	0.98	0.83	0.98	0.82	1.16	0.96	0.85	1.11	0.78	1.14	1.13	0.77	1.31	1.12
50	0.64	0.97	1.33	1.38	0.94	1.02	0.92	1.06	0.89	0.72	1.04	1.30	0.89	0.92	0.85	1.28	0.89	1.08	0.70	0.60	1.06	1.08	0.67	1.09	1.16
51	0.94	0.91	1.20	1.12	1.45	0.82	1.23	1.49	1.26	1.45	0.69	0.74	0.88	0.97	0.95	1.15	0.87	1.27	0.89	0.91	1.28	0.76	0.83	1.34	1.07
52	1.26	0.87	1.09	1.26	1.30	1.11	1.20	1.02	1.22	1.11	0.70	0.76	0.74	1.00	1.07	1.19	0.94	1.09	0.94	1.14	1.26	0.87	0.83	1.32	1.09
53	1.45	0.93	1.17	1.19	0.90	1.02	1.16	1.41	1.13	1.05	0.81	0.82	0.69	1.11	0.94	1.11	1.16	1.02	1.04	1.09	1.71	0.78	0.85	1.32	1.13
54	1.10	0.86	0.90	1.01	1.20	1.13	1.17	1.34	1.02	1.08	0.85	0.88	0.77	1.17	0.71	1.07	1.03	0.87	0.80	1.22	1.19	0.99	0.84	1.28	1.09
55	1.11	0.87	1.09	1.05	1.07	1.23	1.20	0.97	1.19	1.14	0.80	0.97	0.79	1.00	1.01	1.16	1.16	0.86	0.84	1.20	1.29	1.11	0.79	1.14	1.16
56	1.17	0.86	1.06	1.17	1.03	1.19	1.11	1.08	1.10	0.92	0.82	1.01	0.89	1.17	0.97	1.07	1.29	0.93	0.92	1.09	1.18	0.92	0.82	1.08	1.09
57	0.73	1.06	1.00	0.81	1.20	0.74	0.68	1.31	0.78	0.97	0.69	1.02	0.91	1.13	0.90	0.88	0.64	1.19	0.62	1.50	1.44	0.75	0.72	1.19	0.79
58	1.08	1.19	0.99	0.98	1.17	0.97	0.85	1.03	0.97	0.90	0.77	0.87	0.68	1.23	0.96	0.95	0.77	1.08	0.76	1.32	1.30	1.03	0.91	1.29	0.79
59	0.93	1.12	1.00	0.93	1.04	0.91	0.78	1.09	1.09	0.84	0.93	1.02	0.68	1.32	0.76	0.97	0.93	0.80	0.77	1.56	1.07	1.03	0.91	1.12	0.82
60	0.99	1.44	1.06	0.92	0.92	1.05	0.91	0.84	1.11	0.83	0.85	1.22	0.70	1.23	0.73	0.87	0.95	0.85	0.77	1.55	1.68	0.98	0.77	1.01	0.79
61	0.83	1.33	1.21	1.22	1.10	1.65	0.82	0.79	0.98	0.98	1.17	1.21	0.65	1.31	0.91	0.92	0.83	0.70	0.70	1.39	1.29	0.90	0.85	1.09	0.96
62	0.81	1.26	1.29	1.01	0.99	1.03	0.75	0.92	0.83	1.07	0.90	1.36	0.75	1.20	0.82	0.93	1.07	0.94	0.97	1.35	1.05	0.91	0.89	1.15	1.13
63	0.86	1.13	1.22	0.93	1.15	1.06	0.64	0.68	0.81	1.02	0.99	1.36	0.76	1.10	0.99	0.95	0.92	1.00	0.88	1.26	1.02	0.69	0.84	1.13	0.91
64	0.64	1.08	1.15	1.05	1.18	1.05	0.78	0.83	0.72	1.01	1.06	1.84	0.84	0.92	0.92	1.11	0.66	1.02	0.81	1.08	1.18	0.72	0.71	0.97	0.92
65	0.90	0.90	0.85	0.69	0.79	0.56	0.80	1.18	0.86	1.05	0.56	0.86	1.14	1.02	0.85	1.01	0.80	1.34	1.00	1.55	1.01	0.91	0.97	1.07	0.91
66	1.17	1.13	0.93	0.78	0.95	0.89	0.77	0.93	1.12	0.96	0.78	0.83	1.12	1.26	0.96	0.98	1.04	1.30	0.87	1.53	1.20	1.20	0.97	0.99	1.03
67	1.11	1.19	1.11	0.88	0.98	0.74	0.86	0.76	1.37	0.90	0.71	0.90	0.98	1.15	0.97	1.06	0.95	1.09	0.90	1.32	1.06	1.50	0.91	1.02	0.88
68	1.13	1.15	0.91	0.98	0.99	0.85	0.89	0.80	1.17	0.86	0.77	0.84	1.00	1.08	0.81	0.76	1.09	0.92	0.97	1.71	1.05	1.28	1.07	1.11	0.92
69	0.96	1.28	1.08	0.85	1.04	0.69	0.78	0.69	1.07	1.09	1.33	1.01	1.00	1.09	1.03	0.77	0.95	1.06	0.97	1.31	0.85	1.37	0.87	1.08	0.82
70	1.19	1.15	1.01	1.07	0.86	1.14	0.88	0.65	1.10	1.08	1.43	1.08	0.81	1.22	0.97	1.00	1.13	1.02	1.06	0.95	1.00	1.32	0.92	1.05	0.87
71	0.53	1.70	0.77	1.11	1.29	1.09	0.91	1.64	0.83	0.66	1.37	0.48	2.02	0.86	0.69	1.05	0.81	1.16	1.23	0.89	0.87	0.57	0.94	1.13	2.16
72	0.98	2.32	1.24	1.44	1.47	1.11	1.84	1.42	1.30	0.73	1.05	0.50	1.21	1.13	0.74	1.15	1.06	0.94	1.05	1.13	0.97	0.73	1.27	1.27	2.44
73	0.93	2.25	1.10	1.30	1.27	1.24	1.92	1.33	1.10	0.77	1.01	0.58	1.05	1.15	0.97	2.03	1.13	0.82	0.79	1.09	1.37	0.81	1.08	1.40	1.48
74	1.03	1.77	1.25	1.34	1.33	1.15	1.65	1.18	1.31	0.75	1.37	0.73	0.94	0.90	0.71	0.92	1.14	0.77	0.62	1.08	0.93	0.82	1.18	1.53	1.20
75	0.87	2.06	1.01	1.23	1.40	1.32	1.49	1.02	1.36	0.80	1.41	1.11	0.83	0.93	0.74	0.99	1.21	0.63	0.72	1.11	0.86	0.95	1.29	1.51	1.05
76	0.74	1.68	1.15	1.05	1.92	1.21	1.54	0.89	1.18	0.92	1.25	1.34	0.98	1.08	0.74	0.87	0.99	0.77	0.76	0.80	0.97	0.79	1.32	1.49	1.09
77	1.09	1.60	1.07	1.04	1.90	1.48	1.24	0.89	1.03	0.88	1.43	2.60	1.06	0.98	0.86	0.93	1.02	0.81	0.67	1.03	0.98	0.82	1.16	1.56	1.09
78	0.71	1.08	1.08	1.07	1.66	1.16	1.07	0.78	0.96	0.69	1.64	4.22	0.91	0.96	0.66	0.73	0.74	0.88	0.70	0.83	0.89	0.93	1.65	1.45	1.42
79	0.81	1.32	1.07	1.07	1.29	0.84	0.85	1.65	1.00	0.83	0.90	0.56	1.58	0.93	0.88	0.87	0.78	1.24	1.20	0.92	0.95	0.76	1.21	1.04	1.19
80	1.17	1.67	1.14	1.10	1.89	1.10	1.20	1.43	1.13	0.97	1.18	0.55	1.29	1.17	1.09	0.97	1.18	0.98	0.99	0.97	1.07	0.90	1.11	1.24	1.29
C3	1.01	2.33	1.26	3.40	1.35	0.97	1.16	1.31	0.91	0.90	0.99	0.66	4.67	3.19	4.47	1.11	12.19	0.87	4.07	1.08	1.11	0.84	1.00	1.56	2.28
C32	1.07	2.00	1.62	1.11	1.39	1.08	1.30	1.59	1.02	0.99	1.08	0.77	1.13	0.99	2.11	1.28	1.18	2.43	1.20	1.22	1.65	0.86	1.15	1.21	2.01
KLH	2.34	10.56	10.38	5.48	3.51	7.28	2.33	2.64	2.59	1.05	4.31	2.41	6.89	2.96	14.21	10.05	5.18	2.84	3.55	2.01	12.32	5.60	5.84	2.96	3.21

Mean SI proliferation assay data from donors 26-50 in the adjusted data set for peptides 43-80. Numbers in red indicate positive responses (SI ≥2.00, p <0.05). Numbers in pink indicate borderline responses (SI ≥1.90, p <0.05).

PEPTIDE	26	27	28	29	30	31	32	33	34	35	36	37	38	39	40	41	42	43	44	45	46	47	48	49	50
43	1.03	0.51	0.79	1.08	2.89	0.78	1.33	0.86	0.76	1.20	0.68	1.08	1.09	0.91	1.00	1.34	0.94	0.77	0.72	0.79	0.67	0.85	0.91	0.99	1.04
44	1.30	0.55	0.82	1.24	1.65	0.75	1.22	0.80	0.79	1.23	0.78	1.24	1.31	0.90	0.97	1.19	1.11	0.86	1.00	0.74	0.93	0.92	0.81	0.91	1.10
45	1.22	0.61	0.78	1.26	2.18	0.88	1.43	0.81	0.94	1.23	0.83	1.10	1.08	0.79	0.79	1.14	0.94	0.94	0.94	0.81	1.02	1.02	0.81	0.82	1.11
46	1.03	0.71	0.72	1.12	1.62	0.87	0.95	0.95	0.91	1.17	0.98	1.00	1.03	1.01	0.67	1.12	1.10	0.66	0.90	0.86	1.09	0.97	0.84	1.08	0.92
47	1.10	0.75	0.78	1.11	1.18	0.93	0.88	0.85	0.75	1.31	0.94	1.13	1.18	0.96	0.89	1.51	0.90	0.58	1.12	0.82	0.95	0.85	0.86	0.89	0.89
48	0.90	0.71	0.83	0.98	1.12	0.75	1.27	0.84	0.82	1.24	0.95	1.10	1.17	0.93	0.88	1.62	0.98	0.63	1.31	0.91	1.13	0.94	0.81	0.77	1.01
49	0.84	0.75	0.89	1.00	1.04	1.00	1.16	0.84	0.83	1.16	1.12	1.29	1.30	0.95	0.83	1.65	0.94	0.86	0.87	0.90	0.94	0.87	0.93	0.93	1.04
50	0.83	0.79	0.97	0.97	1.19	0.92	1.16	1.09	0.91	1.10	1.25	0.98	1.27	1.25	0.82	1.84	1.14	0.69	1.08	0.69	0.96	0.85	1.03	0.65	0.91
51	1.04	0.49	0.99	1.08	1.49	0.94	1.38	0.75	0.86	1.07	0.58	1.13	1.22	0.92	0.93	1.16	1.02	0.83	0.87	0.92	0.92	1.00	0.97	1.07	1.04
52	1.18	0.66	0.82	1.21	1.13	0.98	1.35	0.67	0.87	1.02	0.60	1.05	1.19	0.87	0.92	1.06	1.15	0.84	0.83	0.95	0.86	0.80	0.89	0.92	0.97
53	0.99	0.68	0.92	1.18	1.37	0.88	0.93	0.78	0.84	1.03	0.77	0.95	1.16	0.90	0.88	1.09	1.20	0.81	1.40	1.04	0.98	0.87	1.02	0.86	1.05
54	1.05	0.76	0.92	1.20	1.23	0.99	1.14	0.77	0.87	0.86	0.77	0.99	1.01	0.82	0.98	1.00	1.08	0.63	1.13	1.08	0.95	0.89	0.97	1.01	0.91
55	1.21	0.65	0.81	1.00	1.26	0.81	0.60	0.98	0.79	0.97	0.71	0.96	0.93	0.93	0.95	1.43	1.05	0.87	1.35	1.01	0.99	1.12	0.75	0.90	0.79
56	0.99	0.73	0.91	1.19	1.16	0.94	1.01	0.82	0.92	1.03	0.90	0.96	0.83	1.10	0.86	1.22	0.98	0.80	1.08	0.96	1.00	1.03	0.91	0.82	1.02
57	1.42	0.51	1.21	1.14	1.34	1.21	1.04	1.09	0.86	1.09	0.86	0.98	1.06	1.23	1.19	1.33	1.23	1.45	0.66	0.72	1.06	1.10	1.15	0.80	1.05
58	1.40	0.74	1.18	1.09	1.22	1.12	1.10	0.90	1.01	1.25	1.11	0.94	1.17	1.30	1.29	1.20	1.08	1.38	0.95	0.77	0.93	1.13	1.03	1.12	0.85
59	1.56	0.76	1.05	1.18	1.34	1.05	1.42	0.71	0.85	1.23	0.82	1.18	1.22	1.31	1.10	1.14	1.19	1.06	0.60	0.81	1.03	1.06	0.90	0.84	0.95
60	1.36	0.70	0.96	1.10	1.12	1.02	1.30	0.98	0.93	1.05	0.95	1.31	1.04	1.21	1.15	0.92	1.10	1.27	0.60	0.82	1.08	1.03	0.77	0.67	0.85
61	1.42	0.88	0.94	1.16	1.05	0.91	1.38	1.08	0.93	1.15	1.27	1.35	1.14	1.19	1.17	1.32	0.97	0.95	0.57	0.66	1.14	1.15	0.79	0.81	0.82
62	1.35	1.08	1.14	1.22	0.79	1.15	1.25	1.03	0.95	1.04	1.06	1.06	1.09	1.40	1.05	0.96	1.04	1.12	0.77	0.72	1.05	0.86	0.79	0.77	0.72
63	1.27	0.96	1.08	1.14	0.91	1.20	1.23	1.03	0.96	1.02	1.22	1.24	1.21	1.16	1.10	1.15	1.09	1.15	0.92	0.75	1.05	1.06	0.68	1.00	0.65
64	1.14	1.04	1.03	1.28	1.06	1.50	1.12	0.98	0.71	1.07	0.97	1.30	1.44	1.13	0.88	1.04	1.06	0.98	1.08	0.79	0.95	1.21	0.86	0.80	0.54
65	1.35	0.49	1.04	0.87	0.94	1.00	0.81	1.24	0.79	1.33	0.84	1.29	1.02	1.06	1.11	1.46	1.06	1.48	0.70	0.91	0.88	1.02	1.33	1.08	1.08
66	1.40	0.58	1.13	1.09	1.22	1.11	0.86	0.91	1.18	1.50	1.00	1.14	1.26	0.90	1.09	1.55	1.11	1.33	0.67	0.92	1.18	0.94	1.04	1.22	0.98
67	1.23	0.73	0.94	0.95	1.33	1.29	0.92	0.72	0.77	1.22	0.68	1.17	0.86	1.15	1.13	1.19	1.13	1.11	0.71	0.89	1.19	0.83	1.06	0.88	1.07
68	1.17	0.90	0.87	1.20	1.00	0.91	1.24	1.09	1.19	1.27	0.83	0.91	0.90	1.08	0.91	0.93	1.05	1.13	0.66	0.84	1.03	0.98	1.03	0.94	1.16
69	1.17	0.94	0.74	1.08	1.03	0.94	1.15	0.75	1.07	1.01	0.91	1.17	0.88	1.07	1.05	1.03	1.18	0.97	0.55	0.92	0.92	0.90	0.94	1.09	0.83
70	1.11	0.88	0.71	1.12	0.91	0.92	1.03	0.94	0.86	1.01	1.02	1.31	0.88	1.10	0.93	0.94	1.08	0.95	0.68	0.80	0.99	0.68	1.00	0.74	0.88
71	1.12	0.64	0.85	0.87	1.02	0.78	0.71	4.71	0.88	0.94	0.82	1.21	0.72	0.50	1.03	0.92	1.90	1.04	3.60	0.88	0.77	1.00	0.94	0.91	1.42
72	1.22	0.54	1.07	1.35	1.31	0.96	0.78	4.50	1.07	1.26	0.66	1.08	0.76	0.79	0.95	1.20	1.82	1.11	10.60	0.93	1.00	1.16	0.86	0.90	1.18
73	1.15	0.57	1.10	1.25	1.09	0.72	0.71	1.67	1.21	1.41	0.70	1.01	0.89	0.69	0.90	0.95	2.11	1.07	0.80	0.83	1.12	1.21	0.86	0.93	1.47
74	1.19	0.74	0.94	1.03	1.07	0.72	1.04	1.51	0.82	1.37	0.77	1.20	0.86	0.78	0.77	1.10	1.79	1.03	1.09	0.97	0.98	1.08	0.74	0.93	1.43
75	1.30	0.99	1.02	1.01	1.06	0.71	0.86	1.89	1.08	1.16	0.82	0.99	0.88	0.71	0.84	1.07	1.54	1.21	1.02	0.89	0.93	0.98	0.92	0.82	1.26
76	1.08	0.99	0.87	1.20	1.13	0.68	0.96	3.84	0.78	1.41	0.80	1.06	0.85	0.81	0.65	0.91	1.22	0.92	1.23	0.85	1.00	0.85	0.79	0.80	1.18
77	1.32	0.90	1.02	0.82	1.09	0.87	1.01	1.36	0.63	1.49	0.89	1.08	0.90	0.90	0.57	0.84	1.29	0.78	0.80	0.80	0.91	0.73	0.64	0.72	0.93
78	1.00	0.98	0.98	0.88	1.34	0.85	0.98	2.21	0.77	1.11	1.25	1.23	1.02	2.70	0.66	0.76	1.23	0.82	1.03	0.83	0.79	0.53	0.74	0.92	0.64
79	1.17	0.89	1.02	1.44	1.22	1.37	0.89	1.41	1.31	0.99	0.70	0.98	0.95	0.57	1.02	1.44	2.14	1.11	1.32	0.83	1.11	1.66	1.93	1.02	1.72
80	1.04	0.75	1.15	1.32	1.16	1.48	0.64	1.01	1.25	0.92	0.72	0.98	1.05	0.67	0.91	1.39	2.13	1.25	1.06	0.79	0.89	1.14	1.15	1.03	1.65
C3	1.22	0.90	1.07	1.38	11.87	1.00	0.71	1.32	1.28	1.04	0.67	1.11	2.75	1.36	1.00	1.18	1.45	3.42	1.32	1.17	1.10	1.24	1.06	0.74	1.29
C32	1.19	1.18	0.97	1.06	1.20	1.35	5.34	1.87	1.11	1.19	1.45	0.99	1.05	0.83	0.89	1.20	1.49	1.22	1.29	0.89	1.10	6.75	1.89	0.67	1.23
KLH	2.36	9.56	2.45	5.67	7.68	1.39	1.29	9.75	7.26	3.42	3.76	2.18	5.02	3.72	4.61	3.34	6.85	11.38	8.72	13.06	2.87	3.65	15.29	0.64	4.53

Supplementary Methods. Abzena Report 2.
Planning site-directed mutagenesis to eliminate the identified T cell epitopes

An Abzena company
ANTITOPE
Enabling better biopharmaceuticals
*

*** ANTITOPE is a former name of ABZENA (https://abzena.com)**

Research Report: CBL01 Stage 3.2

Cleveland Biolabs, Inc.

Design of Deimmunised Variants

13th November 2015

Antitope Ltd.
Babraham Research Campus
Babraham
Cambridge CB22 3AT
UK

Cleveland BioLabs, Inc.
F.A.O: Vadim Mett, PhD
73 High St.
Buffalo
NY 14203
USA

Signatures

Name	Signature	Date
**Author(s):**		
Rob Holgate	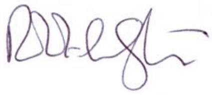	13th November 2015
Matt Smede	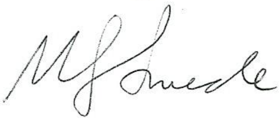	13th November 2015
Arron Hearn	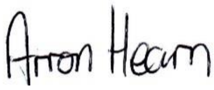	13th November 2015

Additional Contributors:

T. Jones
M. Fogg

Table of Contents

List of Figures 4
List of Tables 4
List of Abbreviations 5
1. Summary 6
2. Introduction 7
3. Design of Variants 8
3.1 Selection of amino acids 8
3.2. Analysis by iTope™ and TCED™ 9
3.2.1 Deimmunisation of epitope 1 11
3.2.2 Deimmunisation of epitope 2 12
3.2.3 Deimmunisation of epitope 3 13
4. Conclusion 17
5. References 18

List of Figures

Figure 1. Structural analysis of Entolimod together with TLR5 generated using Swiss-PDBViewer. The predicted structure was prepared by aligning the helical regions of entolimod (structure provided by Cleveland Biolabs) with the helical regions of flagellin previously complexed with TLR5 (PDB I.D 3V47, Yoon *et al.* 2012). Two TLR5-flagellin 1:1 heterodimers are shown assembled into a 2:2 tail-to-tail complex. For clarity, only one molecule of entolimod is shown (in red) complexed with one molecule of TLR5 (in blue) with the other 1:1 TLR5-flagellin heterodimer shown in grey. T cell epitopes 1, 2 and 3 are highlighted. 9

Figure 2. Peptide 6 showing two HLA-DR restricted core 9mer binding registers in Epitope 1. The two potential registers are highlighted in blue and the P1 anchor residues are highlighted in red. Proposed mutations for deimmunisation are shown. Mutations affecting I83 only are shown in black, mutations affecting F87 only are shown in purple and mutations affecting both I83 and F87 are shown in green. 11

Figure 3. Peptides 71-74 showing three HLA-DR restricted core 9mer binding registers in Epitope 2. The three potential registers are highlighted in blue and the P1 anchor residues are highlighted in red. Proposed mutations for deimmunisation are shown. Mutations affecting I305 only are shown in black, mutations affecting L306 only are shown in purple and mutations affecting V313 only are shown in orange. Mutations affecting more than one p1 anchor are shown in green (I305 and L306) or blue (L306 and V313). 12

Figure 4. Peptides 77-80 showing four HLA-DR restricted core 9mer binding registers in Epitope 3. The four potential registers are highlighted in blue and the P1 anchor residues are highlighted in red. Proposed mutations for deimmunisation are shown. Mutations affecting V324 only are shown in black, mutations affecting L325 only are shown in purple and mutations affecting both V324 and L325 are shown in green. 13

List of Tables

Table 1. Proposed deimmunising changes to flagellin derivative epitope 1 using iTope™. Peptides spanning the sequence of interest were tested as 9mer peptides in one amino acid increments. The table shows the total number of alleles (out of 34) predicted to bind the epitope and the number of promiscuous high affinity alleles. Mutations in positions affecting more than one P1 anchor residue are highlighted in the same colour.. 14

Table 2. Proposed deimmunising changes to flagellin derivative epitope 2 using iTope™. Peptides spanning the sequence of interest were tested as 9mer peptides in one amino acid increments. The table shows the total number of alleles (out of 34) predicted to bind the epitope and the number of promiscuous high affinity alleles. Mutations in positions affecting more than one P1 anchor residue are highlighted in the same colour.. 15

Table 3. Proposed deimmunising changes to flagellin derivative epitope 3 using iTope™. Peptides spanning the sequence of interest were tested as 9mer peptides in one amino acid increments. The table shows the total number of alleles (out of 34) predicted to bind the epitope and the number of promiscuous high affinity alleles. Mutations in positions affecting more than one P1 anchor residue are highlighted in the same colour.. 16

List of Abbreviations

Abbreviation	*Description*
BLAST	Basic local alignment search tool
CDR	Complementarity Determining Region
FW	Framework region
HLA	Human leukocyte antigen
MHC	Major histocompatibility complex
TCED™	T cell epitope database

1. Summary

The aim of this project was to design a series of epitope variants of a flagellin derivative in such a way that the immunogenic regions, previously identified by T cell epitope mapping (Report: CBL01: Episcreen™ T Cell Epitope Mapping, 26th October 2015), were eliminated whilst retaining binding affinity.

2. Introduction

EpiScreen™ T cell epitope mapping of peptides derived from a flagellin derivative previously revealed positive T cell responses against several peptides (Report: CBL01 EpiScreen™ T Cell Epitope Mapping, 26th October 2015). Sequence analysis of the responding peptides indicated the presence of three T cell epitopes. In each case, these T cell epitopes were low frequency positive T cell proliferation responses, ranging between 6% and 10% of the 50 donor study cohort in the adjusted data. However the mean SIs varied, suggesting a range of T cell epitope strengths from low (epitope 1) to moderate (epitope 3) to strong (epitope 2). The aim of this project was to design a series of epitope variants of the flagellin derivative in such a way that the identified immunogenic regions were removed whilst taking into account structural and likely binding properties of the flagellin derivative.

3. Design of Variants

3.1 Selection of amino acids

Selection of specific amino acid changes was influenced by the available biophysical and biochemical data, e.g. constraints on modification of the reference flagellin structure taking into consideration secondary and tertiary protein structures, as well as potential interactions of amino acid side chains with the core of the protein and with the ligand, TLR5. Models were based on the structure of flagellin with the N-terminal fragment of zebrafish TLR5 (Yoon *et al,* 2012). Each epitope was analysed individually to identify residues that would remove promiscuous MHC class II binding. All three epitopes were located away from the principle binding site with TLR5 (**Figure 1**), and were thus were not predicted to interfere directly with ligand binding. The structure of flagellin was noted as primarily comprising of alpha helical regions, therefore helix breaking glycine and proline residues were avoided when selecting alternative amino acids.

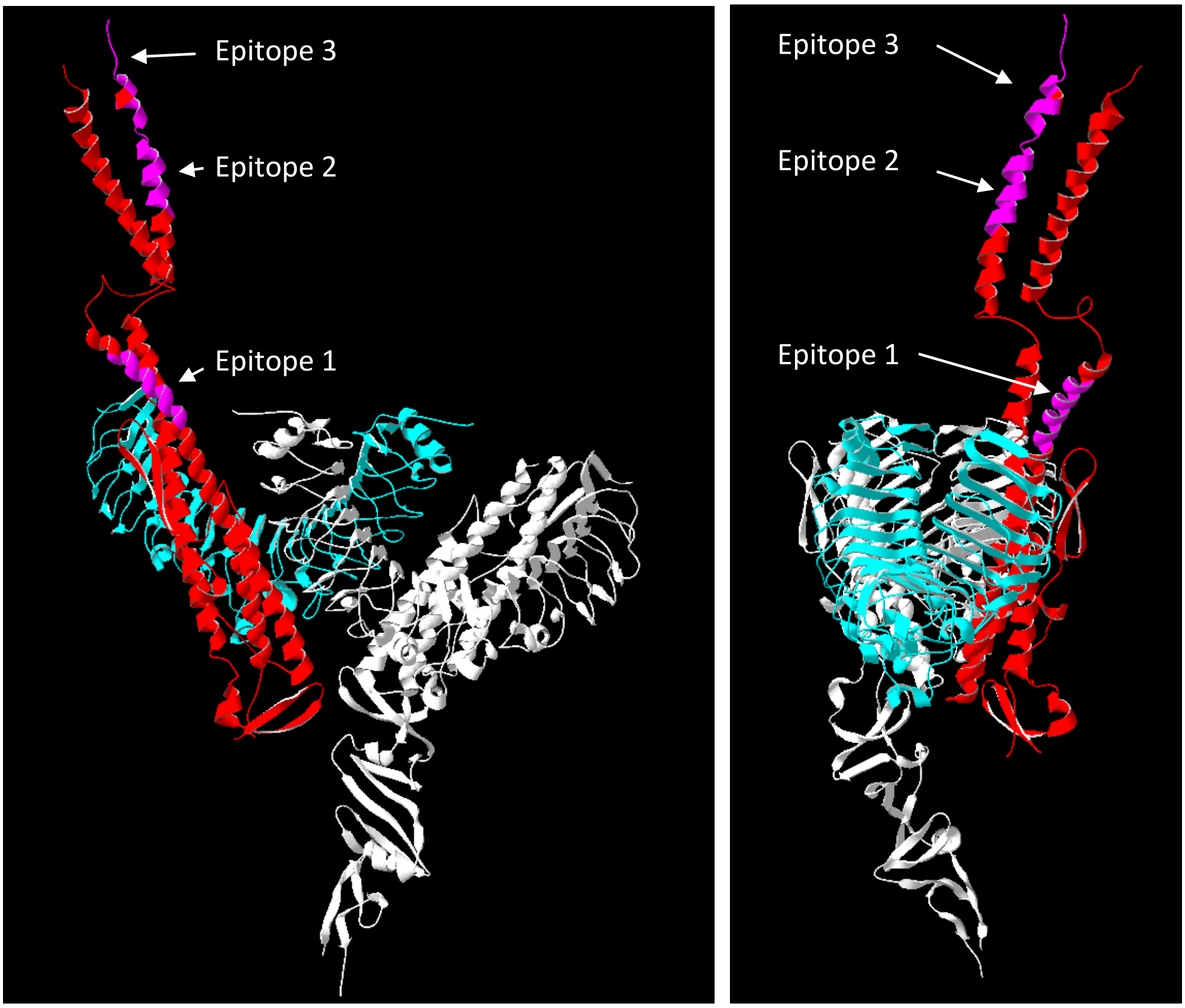

Figure 1. Structural analysis of Entolimod together with TLR5 generated using Swiss-PDBViewer. The predicted structure was prepared by aligning the helical regions of entolimod (structure provided by Cleveland Biolabs) with the helical regions of flagellin previously complexed with TLR5 (PDB I.D 3V47, Yoon *et al.* 2012). Two TLR5-flagellin 1:1 heterodimers are shown assembled into a 2:2 tail-to-tail complex. For clarity, only one molecule of entolimod is shown (in red) complexed with one molecule of TLR5 (in blue) with the other 1:1 TLR5-flagellin heterodimer shown in grey. T cell epitopes 1, 2 and 3 are highlighted.

3.2. Analysis by iTope™ and TCED™

The iTope™ software predicts favourable interactions between amino acid side chains of a peptide and specific binding pockets (in particular pocket positions; p1, p4, p6, p7 and p9) within the open-ended binding grooves of 34 human MHC class II alleles. These alleles represent the most common HLA-DR alleles found world-wide with no weighting attributed to those found most prevalently in any particular ethnic population. Twenty of the alleles contain the 'open' p1 configuration and 14 contain the 'closed' configuration where glycine

at position 83 is replaced by a valine. The location of key binding residues is achieved by the *in silico* generation of 9mer peptides that overlap by one amino acid spanning the test protein sequence. In-house comparisons with physical MHC class II binding experiments has shown that iTope™ can be used to successfully discriminate with high accuracy between peptides that either bind or do not bind MHC class II molecules. However, the results should be assessed in the light of the fact that all predictive methods for MHC class II binding inherently over-predict the number of T cell epitopes since they do not allow for other important processes during antigen presentation such as protein/peptide processing, recognition by the T cell receptor or T cell tolerance to the peptide. The TCED™ contains the sequences of all the peptides previously screened in EpiScreen™ T cell epitope mapping assays. The TCED™ is used to search any test sequence against a large (>10,000 peptides) database of peptides derived from unrelated protein and antibody sequences which have been tested in EpiScreen™ T cell epitope mapping assays.

Analysis of the derivative flagellin sequences using iTope™ was performed with overlapping 9mers spanning each of the three DR-restricted epitopes identified previously, and were tested against each of the 34 MHC class II allotypes. Each 9mer was scored based on the potential 'fit' and interactions with the MHC class II molecules. The peptide scores calculated by the software lie between 0 and 1. Peptides that produced a high mean binding score (>0.55 in the iTope™ scoring function) were highlighted and, if >50% of the MHC class II binding peptides (i.e. 17 out of 34 alleles) had a high binding affinity (score >0.6), such peptides were defined as 'promiscuous high affinity' MHC class II binding peptides which are considered a high risk for containing $CD4^+$ T cell epitopes. Promiscuous moderate affinity MHC class II binding peptides bind a high number of alleles (>50%) with a binding score >0.55 (but without a majority >0.6).

3.2.1 Deimmunisation of epitope 1

In silico MHC class II binding analysis using iTope™ revealed two possible core 9mer HLA-DR restricted epitopes, with two associated P1 anchor residues (I83 and F87, numbering based on Entolimod reference sequence), although the iTope™ data suggests that I83 (17 alleles bound in total) is more likely to be the P1 anchor than F87 (8 alleles bound). As this region is primarily alpha-helical, glycine and proline (both known helix breakers) were avoided, as were changes that might disrupt the helix-helix interface. Suggested sequence changes were identified that affect either one or both of the epitopes associated with the two potential P1 anchors and are illustrated in **Figure 2** and listed in **Table 1**. As the precise location of the P1 anchor is not known, mutations affecting both residues would be the preferred choice.

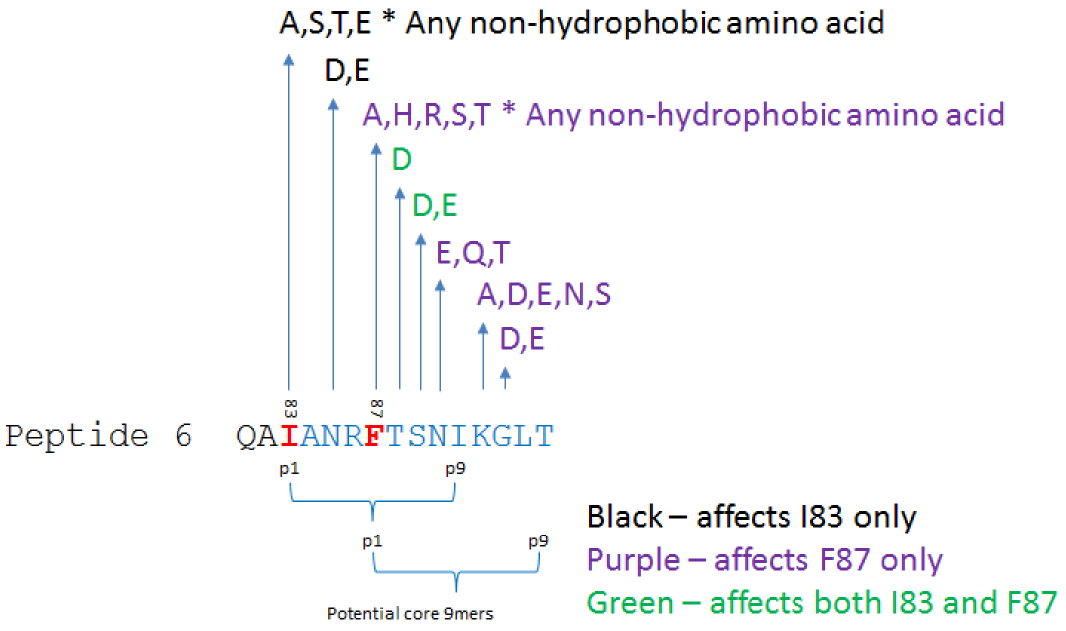

Figure 2. Peptide 6 showing two HLA-DR restricted core 9mer binding registers in Epitope 1. The two potential registers are highlighted in blue and the P1 anchor residues are highlighted in red. Proposed mutations for deimmunisation are shown. Mutations affecting I83 only are shown in black, mutations affecting F87 only are shown in purple and mutations affecting both I83 and F87 are shown in green.

3.2.2 Deimmunisation of epitope 2

In silico MHC class II binding analysis using iTope™ revealed three possible core 9mer HLA-DR restricted epitopes, with three associated P1 anchor residues (I305, L306 and V313, numbering based on Entolimod reference sequence). As this region is primarily alpha-helical, glycine and proline (both known helix breakers) were avoided, as were changes that might disrupt the helix-helix interface. Suggested single amino acid sequence changes, primarily based on the predicted HLA-DR associations, are illustrated in **Figure 3** and listed in **Table 2**. From iTope™ analysis, the 9mers associated with the three P1 anchors (I305, L306 and V313) were predicted to bind 2, 29 and 9 out of 34 MHC class II alleles respectively. Therefore, mutations affecting L306 would be the preferred choice (shown in purple, green and blue in **Figure 3**).

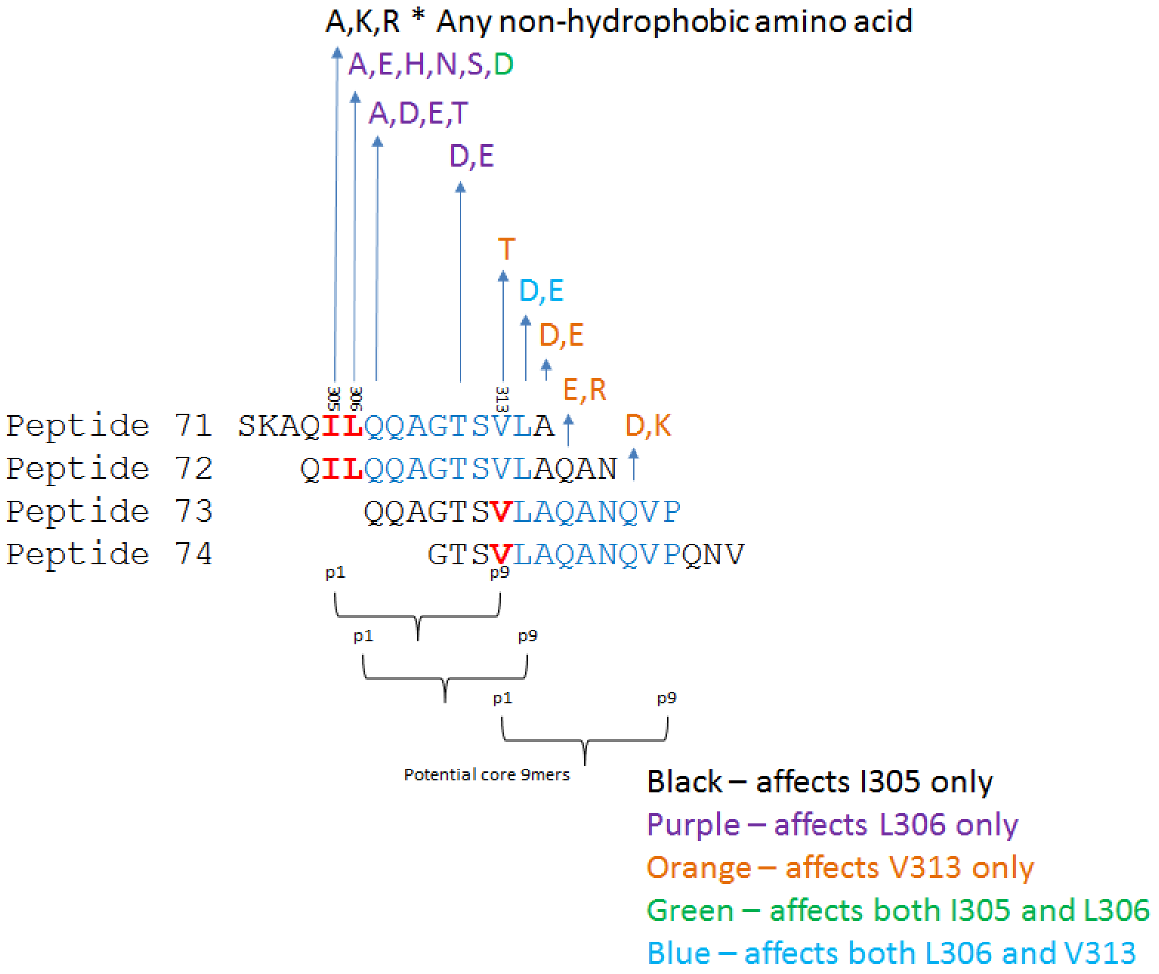

Figure 3. Peptides 71-74 showing three HLA-DR restricted core 9mer binding registers in Epitope 2. The three potential registers are highlighted in blue and the P1 anchor residues are highlighted in red. Proposed mutations for deimmunisation are shown. Mutations affecting I305 only are shown in black, mutations affecting L306 only are shown in purple and mutations affecting V313 only are shown in orange. Mutations affecting more than one p1 anchor are shown in green (I305 and L306) or blue (L306 and V313).

3.2.3 Deimmunisation of epitope 3

In silico MHC class II binding analysis using iTope™ revealed four possible core 9mer HLA-DR restricted epitopes with four associated P1 anchor residues (V324, L325, L327 and L328, numbering based on Entolimod sequence) within peptide 78. These 9mers were predicted to bind 29, 29, 2 and 30 out of 34 MHC class II alleles respectively. Since peptides 79 and 80 failed to induce positive responses in the T cell assay it is likely that the P1 anchor is either V324 or L325. Structural analysis suggests a flexible conformation within this region thus allowing for consideration of a wider range of amino acids for incorporation. Suggested single amino acid sequence changes predicted to affect the HLA-DR associations of these two P1 anchors are illustrated in **Figure 4** and listed in **Table 3**.

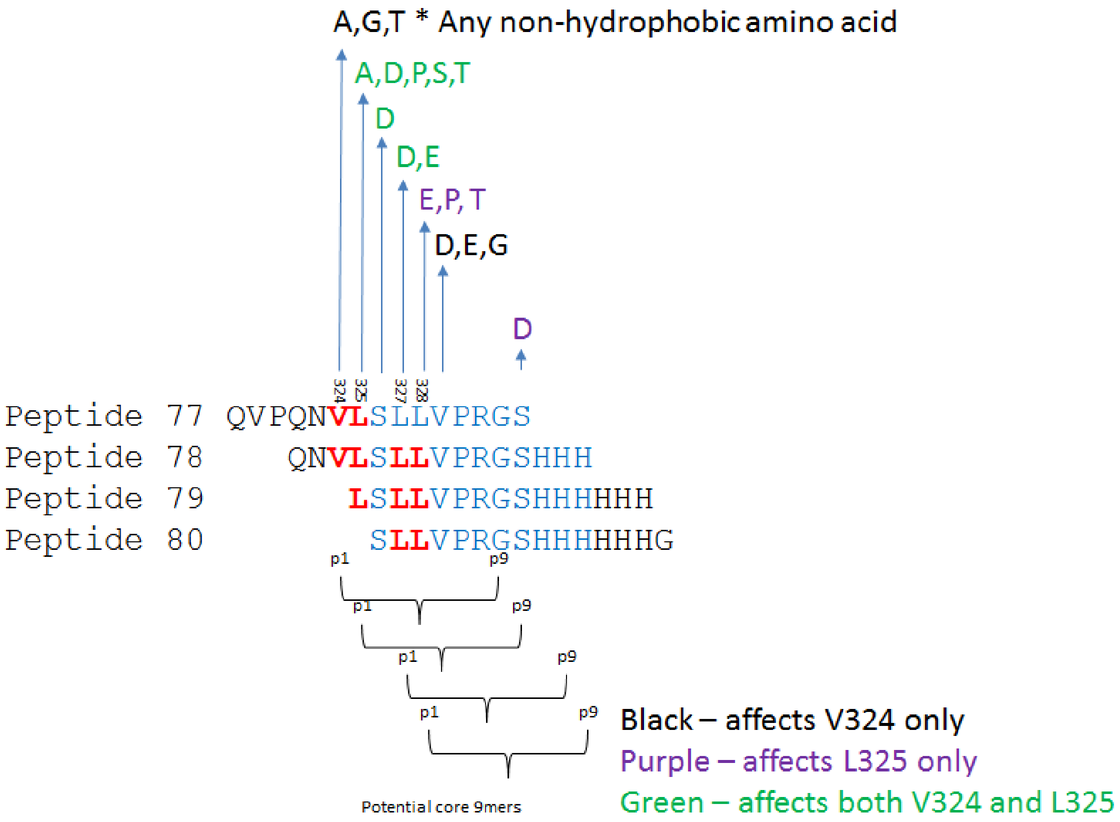

Figure 4. Peptides 77-80 showing four HLA-DR restricted core 9mer binding registers in Epitope 3. The four potential registers are highlighted in blue and the P1 anchor residues are highlighted in red. Proposed mutations for deimmunisation are shown. Mutations affecting V324 only are shown in black, mutations affecting L325 only are shown in purple and mutations affecting both V324 and L325 are shown in green.

p1 Anchor	Sequence	MHC II Ligands	High Affinity Ligands
I83	IANRFTSNI	17	6
	A********	0	0
	S********	0	0
	T********	0	0
	E********	0	0
	**D******	5	1
	**E******	5	2
	*****D***	0	0
	******D**	7	5
	******E**	12	5

F87	FTSNIKGLT	8	2
	A********	0	0
	H********	0	0
	R********	0	0
	S********	0	0
	T********	0	0
	*D*******	1	0
	**D******	0	0
	**E******	0	0
	E**	0	0
	Q**	4	1
	T**	4	0
	*****A***	2	1
	*****D***	0	0
	*****E***	0	0
	*****N***	3	0
	*****S***	1	0
	******D**	0	0
	******E**	2	0

Table 1. Proposed deimmunising changes to flagellin derivative epitope 1 using iTope™. Peptides spanning the sequence of interest were tested as 9mer peptides in one amino acid increments. The table shows the total number of alleles (out of 34) predicted to bind the epitope and the number of promiscuous high affinity alleles. Mutations in positions affecting more than one P1 anchor residue are highlighted in the same colour.

p1 Anchor	Sequence	MHC II Ligands	High Affinity Ligands
I305	ILQQAGTSV	2	2
	A********	0	0
	K********	0	0
	R********	0	0
	*D*******	2	1

L306	LQQAGTSVL	29	10
	A********	0	0
	D********	0	0
	E********	0	0
	H********	0	0
	N********	0	0
	S********	0	0
	*A*******	10	7
	*D*******	5	0
	*E*******	10	7
	*T*******	10	7
	*****D***	4	1
	*****E***	3	2
	********D	17	10
	********E	19	10

V313	VLAQANQVP	9	6
	T********	0	0
	*D*******	0	0
	*E*******	6	1
	**D******	4	0
	**E******	4	0
	E**	4	3
	R**	6	1
	******D**	3	2
	******K**	6	3

Table 2. Proposed deimmunising changes to flagellin derivative epitope 2 using iTope™. Peptides spanning the sequence of interest were tested as 9mer peptides in one amino acid increments. The table shows the total number of alleles (out of 34) predicted to bind the epitope and the number of promiscuous high affinity alleles. Mutations in positions affecting more than one P1 anchor residue are highlighted in the same colour.

p1 Anchor	Sequence	MHC II Ligands	High Affinity Ligands
V324	VLSLLVPRG	29	24
	A********	0	0
	G********	0	0
	T********	0	0
	*A*******	22	13
	*D*******	7	3
	*P*******	18	5
	*S*******	22	8
	*T*******	22	13
	**D******	18	5
	D**	16	7
	E**	16	4
	*****D***	4	1
	*****E***	6	2
	*****G***	13	5

L325	LSLLVPRGS	29	21
	A********	0	0
	D********	0	0
	P********	0	0
	S********	0	0
	T********	0	0
	*D*******	21	17
	**D******	15	5
	**E******	15	6
	E**	18	9
	P**	16	6
	T**	19	13
	********D	20	14

Table 3. Proposed deimmunising changes to flagellin derivative epitope 3 using iTope™. Peptides spanning the sequence of interest were tested as 9mer peptides in one amino acid increments. The table shows the total number of alleles (out of 34) predicted to bind the epitope and the number of promiscuous high affinity alleles. Mutations in positions affecting more than one P1 anchor residue are highlighted in the same colour.

4. Conclusion

Sequence variants of the flagellin derivative were analysed by iTope™ and TCED™ and changes were predicted within the sequences of Epitopes 1, 2 and 3 that significantly reduced or eliminated MHC class II binding in order to remove associated T cell epitopes.

5. References

Yoon SI, Kurnasov O, Natarajan V, Hong M, Gudkov AV, Osterman AL, Wilson IA. Structural basis of TLR5-flagellin recognition and signaling. Science. 2012 Feb 17;335 (6070):859-64.

Supplementary Methods. Abzena Report 3.
Comparing immunogenicity of the engineered entolimod variant GP532 with parental entolimod (here termed Sample 2 and Sample 1, respectively)

Final report: CBL02 **Cleveland Biolabs, Inc.**

EpiScreen™ DC:T Cell Immunogenicity Analysis

19th June 2018

Abzena plc
Babraham Research Campus
Babraham
Cambridge
CB22 3AT
UK

Cleveland Biolabs, Inc.
FAO: Dr. Vadim Mett
73 High St.
Buffalo
New York 14203
USA

Antitope Ltd, trading as Abzena, is a wholly owned UK operating subsidiary of the UK registered Abzena plc group

Signatures

Author(s):

Name	Signature	Date
E. A. Cloake		19th June 2018
M. H. Fogg		19th June 2018

Additional Contributors:

A. Rust
T. Fisher
K. Welch
A. Caprifico
J. Csapo
E. Mallett
R. Fisher
S. Snelling
B. Hajir
R. Cunningham

Table of Contents

List of Figures 4
List of Tables 5
List of Abbreviations 6
1. Summary 7
2. Introduction 8
3. Methods 9
3.1 EpiScreen™ donor selection 9
3.2 Preparation of samples 10
3.3 Preparation of MoDC and autologous CD4+ T cells 12
3.4 Proliferation assays 12
3.5 ELISpot assays 12
3.6 Assessment of cell viability 13
3.7 EpiScreen™ data analysis 13
3.8 Statistical analysis 13
4. Results and Discussion 14
4.1 Assessment of cell viability 14
4.2 Screening of samples using EpiScreen™ DC:T cell assays 15
4.2.1 EpiScreen™ DC:T cell proliferation assay 15
4.2.2 EpiScreen™ DC:T cell IL-2 ELISpot assay 19
4.3 Interpretation of results 23
5. Conclusion 25
6. References 26
Appendix I 27
Appendix II 29
Appendix III 30

List of Figures

Figure 1. Frequency of donor HLA-DR and DQ allotypes expressed in CBL02 study compared to the world population and combined European and North American populations. 9

Figure 2. Reducing SDS-PAGE of the samples and a reference antibody. The samples and a reference antibody were loaded at (a) 0.1µg and (b) 1µg onto NuPage 4-12% Bis-Tris gels (ThermoFisher Scientific) and run at 200 V for 30 min. Size marker is PageRuler broad range unstained protein ladder (ThermoFisher Scientific). Gels were stained with a Pierce Silver Stain Kit (ThermoFisher Scientific). 11

Figure 3. Viability of MoDC from 10 donors cultured with the samples and controls (medium and KLH) was assessed by trypan blue dye exclusion and is shown as an average with SD (error bars). 15

Figure 4. Healthy donor T cell proliferation responses to: (a) sample 1, (b) sample 2 and (c) KLH. $CD4^+$ T cells were incubated with autologous mature DC loaded with the samples and assessed for proliferation after 7 days' incubation. T cell responses with an SI ≥1.90 (indicated by red dotted line) that were significant ($p <0.05$) using an unpaired, two sample Student's t-test were considered positive. 19

Figure 5. Healthy donor T cell IL-2 secretion response to: (a) sample 1, (b) sample 2 and (c) KLH. $CD4^+$ T cells were incubated with autologous mature DC loaded with the samples and assessed for IL-2 secretion after 7 days' incubation. T cell responses with an SI ≥1.90 (indicated by red dotted line) that were significant ($p <0.05$) using an unpaired, two sample Student's t-test were considered positive. 22

Figure 6. Box and whisker plots showing healthy donor T cell responses to the samples: (a) Proliferation of $CD4^+$ T cells and (b) T cell IL-2 ELISpot responses. Bars represent the 10-90 percentile. * $p < 0.05$ 24

List of Tables

Table 1. Details of CBL02 samples received for EpiScreen™ DC:T cell immunogenicity analysis. The endotoxin levels were determined by a chromogenic kinetic LAL assay kit. 10

Table 2. Summary of healthy donor T cell proliferation and IL-2 ELISpot responses. Positive T cell responses for proliferation (SI ≥1.90, p <0.05) (“P”), and IL-2 (SI ≥1.90, p <0.05) ELISpot (“E”) after 7 days’ culture are shown. The frequency of positive responses for proliferation and IL-2 ELISpot assays are shown as a percentage at the bottom of the columns. Correlation is expressed as the percentage of proliferation responses that were also positive in the IL-2 ELISpot assay. 16

Table 3. Summary of the mean magnitude (±SD) of positive $CD4^+$ T cell proliferative responses against the samples. The mean SI was calculated from the average of positive donor responses observed during the assay. 17

Table 4. Summary of the frequency and magnitude (±SD) of positive IL-2 secretion responses against the two samples and KLH. The mean SI was calculated from all positive donor responses observed. 20

List of Abbreviations

Abbreviation	*Description*
ANOVA	Analysis of variance
AP	Alkaline phosphatase
BCIP	5-bromo-4-chloro-3-indolyl phosphate
BSA	Bovine serum albumin
CPM	Counts per minute
CV	Coefficient of variance
DC	Dendritic cells
GM-CSF	Granulocyte-Macrophage Colony-Stimulating Factor
HLA	Human leukocyte antigen
HTA	Human Tissue Authority
IL-2	Interleukin 2
IL-4	Interleukin 4
KLH	Keyhole limpet haemocyanin
LAL	Limulus amoebocyte lysate
MoDC	Monocyte-derived dendritic cells
MHC	Major histocompatibility complex
NBT	Nitro blue tetrazolium
PBMC	Peripheral blood mononuclear cells
PBS	Phosphate buffered saline
PHA	Phytohaemagglutinin
SD	Standard deviation
SI	Stimulation Index
SPW	Spots per well
SSO	Sequence specific oligonucleotide
TNF-α	Tumour Necrosis Factor-α

1. Summary

The EpiScreen™ DC:T cell assay was used to assess the immunogenic potential of two samples (provided by Cleveland Biolabs, Inc.) by measuring $CD4^+$ T cell responses. MoDC were prepared from the PBMC of a cohort of 50 healthy donors, loaded with sample (antigen) and induced to a mature phenotype in order to present T cell epitopes to autologous purified $CD4^+$ T cells. T cell responses were measured using T cell proliferation ([^{3}H]-Thymidine uptake) and IL-2 cytokine secretion (ELISpot). Analysis of the frequency and magnitude of the $CD4^+$ T cell responses indicated that sample 1 was considered to have a greater risk of clinical immunogenicity than sample 2.

2. Introduction

Immune responses to biological therapeutic agents are wide ranging, and can be directed against agents that are both non-human and human in origin. These responses include those that elicit a weak clinical effect and those that limit efficacy which can occasionally result in morbidity or even mortality in patients. In particular, serious complications can arise with the production of neutralising antibodies, especially when they target recombinant self-proteins and therefore have the potential to cross react with the patient's own endogenous protein (Lim 2005). Problems associated with immunogenicity to biologics, especially monoclonal antibodies, have been reduced largely due to advances in molecular biology. There are, however, many recombinant protein biologics that are identical to endogenously expressed human sequences that still elicit potent neutralising immune responses in patients (Hochuli 1997; Schellekens et al. 1997; Namaka et al. 2006). The mechanism by which immunogenicity is triggered remains unclear although the tolerance to self-proteins may be broken by a number of factors linked to both the product and the patient (reviewed in: Chester et al. 2005; Baker & Jones 2007). For the product, these include dose, frequency of administration, route, immunomodulatory capacity of the protein therapeutic, and the formulation (Jaber & Baker 2007). For the patient, factors such as immune competence (i.e. whether the patient is receiving immunosuppressive treatment), patient's MHC haplotype and intrinsic tolerance to the protein therapeutic will influence immunogenicity. Regardless of how immunogenicity is triggered, one of the single most important factors in the development of an ensuing immune response is the presence of epitopes that are able to effectively stimulate a potent $CD4^+$ T cell response.

The value of T cell epitope analysis is becoming increasingly important particularly in the pre-clinical analysis of biologics, and may, in time, become a requirement for regulatory approval for clinical trials. To this end, Abzena has developed a pre-clinical *ex vivo* T cell assay (EpiScreen™) which provides an effective technology for assessing immunogenicity by quantifying T cell responses to protein therapeutics. EpiScreen™ T cell assays provide a format in which the immunogenicity of whole proteins can be assessed. Using a cohort of community blood donors carefully selected based on MHC haplotypes, purified therapeutic proteins are tested for T cell immunogenicity *in vitro*. This technology has been used successfully to compare protein variants for the potential to induce an immune response *in vivo* (Jones et al. 2004; Jones et al. 2005). These studies show that EpiScreen™ provides a powerful screening technology due to the high degree of sensitivity along with the robust nature of the assay which allows an accurate pre-clinical assessment of the potential for immunogenicity of biologics.

In the present study, the immunogenicity of two samples provided by Cleveland Biolabs, Inc. was evaluated using the EpiScreen™ DC:T cell assay. DC derived from monocytes isolated from PBMC were loaded with the samples and induced to mature in order to present T cell epitopes to autologous purified $CD4^+$ T cells. T cell responses were assessed using both $CD4^+$ T cell proliferation measured by incorporation of $[^3H]$-Thymidine and IL-2 secretion measured by ELISpot assays in parallel.

3. Methods

3.1 EpiScreen™ donor selection

Antitope Ltd is licensed (number 12627) by the Human Tissue Authority (HTA), the regulatory body for the Human Tissue Act 2004. This act regulates the removal, storage, use and disposal of human tissue within the UK. Accordingly, all activities that fall under this act were performed to HTA standards.

PBMC were isolated from healthy community donor buffy coats (from blood drawn within 24 hours) obtained under consent from commercial vendors. Donors were characterised by identifying HLA-DR and HLA-DQ haplotypes to 4 digit resolution by HISTO Spot SSO HLA typing (MC Diagnostics, St. Asaph, UK). T cell responses to the neo antigen, KLH (Sigma, Poole, UK) were also determined for use as a reproducibility control. PBMC were then frozen and stored in liquid nitrogen until required.

A cohort of 50 donors was selected to best represent the number and frequency of HLA-DR and DQ allotypes expressed in the European/North American and the world populations. Analysis of the allotypes expressed revealed that the cohort covered all major HLA-DR and DQ allotypes. Currently HLA-DP is not considered in the selection, due to its more limited polymorphism and likely low levels of expression (Edwards et al. 1986). **Figure 1** shows a comparison of the distribution and frequency of MHC class II haplotypes expressed in the European/North American and the world populations against the selected donor cohort (CBL02). High resolution HLA-DR and DQ typing results are listed in **Appendix I**.

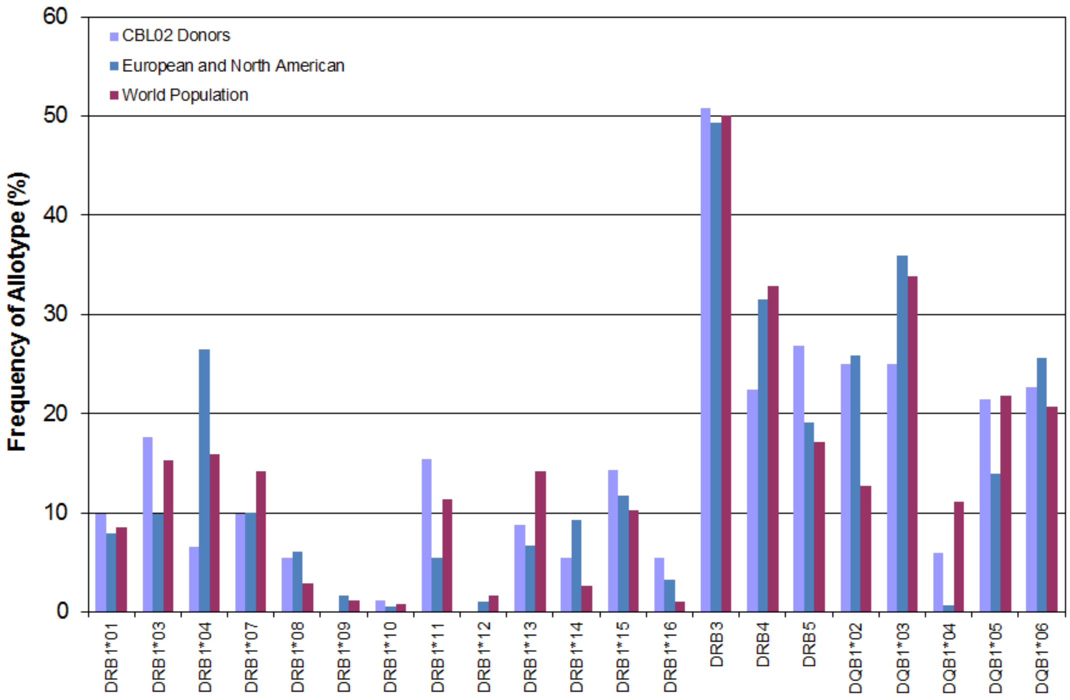

Figure 1. Frequency of donor HLA-DR and DQ allotypes expressed in CBL02 study compared to the world population and combined European and North American populations.

3.2 Preparation of samples

Samples, as detailed in **Table 1**, were received from Cleveland Biolabs, Inc., and were stored according to the instructions provided. The purity of the samples was assessed by denaturing SDS PAGE on a 4-12% gradient gel and silver stained (Pierce Silver Stain Kit, ThermoFisher Scientific, Loughborough, UK). The results of this analysis are shown in **Figure 2**, and indicate that there is one band present in both samples (a reference antibody is shown for comparison). Endotoxin levels were measured using a chromogenic kinetic LAL assay kit according to the manufacturer's instructions (Charles River, Margate, UK) and found to be within the limit acceptable for the assay (<5.0 EU/mg) (**Table 1**). The samples were diluted to 500 µg/ml in AIM-V® culture medium (ThermoFisher Scientific) just before use (final assay concentration 50 µg/ml). KLH was stored at -20°C at 10 mg/ml in dH_2O. For the studies, an aliquot of KLH was thawed immediately before diluting to 1 mg/ml in AIM-V® (final assay concentration 100 µg/ml). PHA (Sigma) was used as a positive control in the ELISpot assay and a 1 mg/ml stock was stored at -20°C before diluting to a concentration of 10 µg/ml in AIM-V® (final assay concentration 2.5 µg/ml).

Abzena ID	Cleveland Biolabs, Inc. ID	Concentration (mg/ml)	Storage	Endotoxin (EU/mg)
Sample 1	CBLB502	1.6	-80°C	0.63
Sample 2	491TEMX	1.0	-80°C	4.62

Table 1. Details of CBL02 samples received for EpiScreen™ DC:T cell immunogenicity analysis. The endotoxin levels were determined by a chromogenic kinetic LAL assay kit.

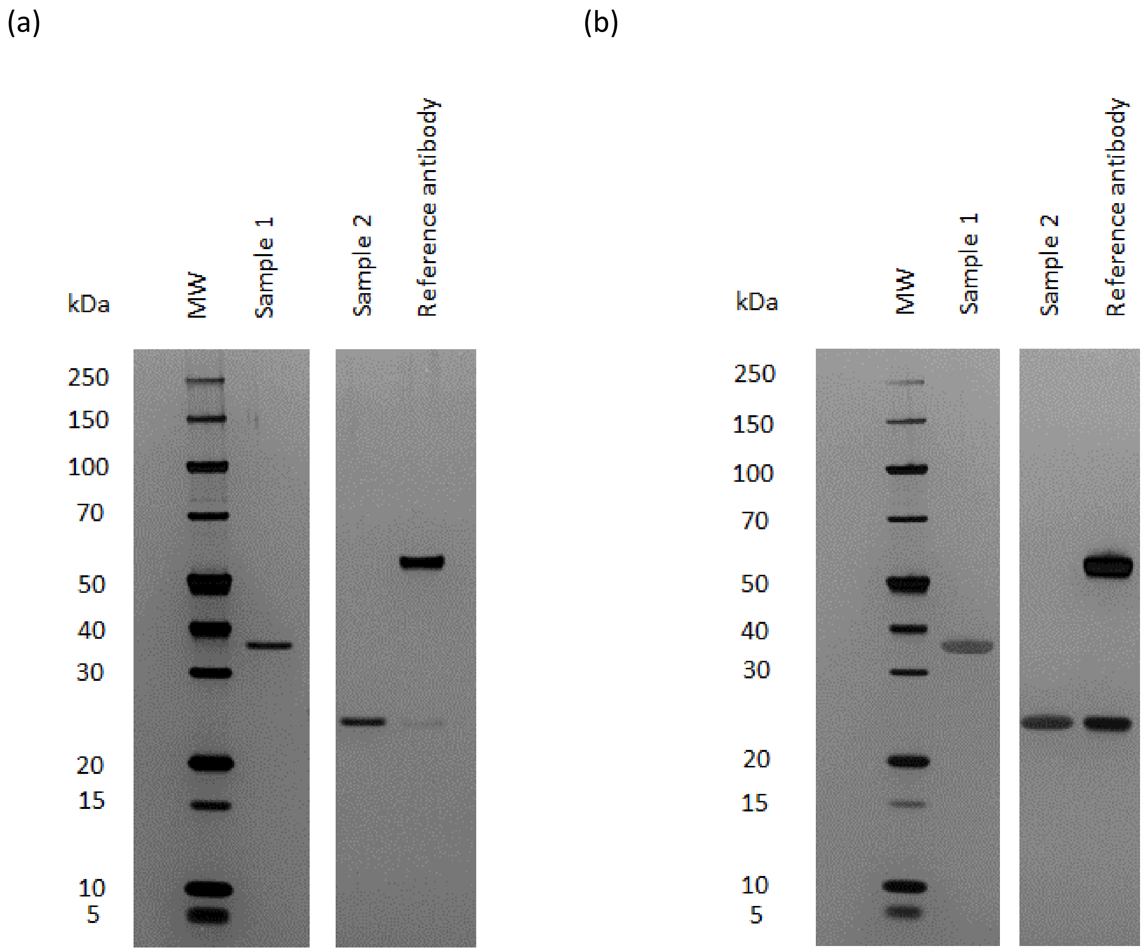

Figure 2. Reducing SDS-PAGE of the samples and a reference antibody. The samples and a reference antibody were loaded at (a) 0.1µg and (b) 1µg onto NuPage 4-12% Bis-Tris gels (ThermoFisher Scientific) and run at 200 V for 30 min. Size marker is PageRuler broad range unstained protein ladder (ThermoFisher Scientific). Gels were stained with a Pierce Silver Stain Kit (ThermoFisher Scientific).

3.3 Preparation of MoDC and autologous CD4$^+$ T cells

To prepare MoDC, PBMC from each donor were revived in AIM-V® culture medium and CD14$^+$ cells (monocytes) were isolated using Miltenyi Pan Monocyte Isolation kits and LS columns (Miltenyi Biotech, Oxford, UK) according to the manufacturer's instructions. Monocytes were resuspended in DC culture media (AIM-V® supplemented with 1000 IU/ml IL-4 and 1000 IU/ml GM-CSF (Peprotech, London, UK)) and plated in low-bind 24 well plates (2 ml final culture volume). Cells were fed on day 2 by half volume DC culture media change. On day 3, antigens (samples and KLH) were added to the cells in DC culture medium to a final concentration of 0.3 µM. In addition, an equivalent volume of DC culture medium was added to the untreated control wells. MoDC were incubated with antigen for 24 hours after which cells were washed three times, and resuspended in DC culture medium containing 50 ng/ml TNF-α (Peprotech) in order to mature the cells.

Cells were fed again on day 7 by a half volume medium change with DC culture medium containing 50 ng/ml TNF-α before harvesting on day 8. The harvested MoDC were counted and viability assessed using trypan blue (Sigma) dye exclusion. MoDC were then γ-irradiated (40 Gy) before use in the proliferation and ELISpot assays. Also on day 8, autologous CD4$^+$ T cells were isolated by negative selection from PBMC using a CD4$^+$ T Cell Isolation Kit and LS columns (Miltenyi Biotech) according to the manufacturer's instructions.

3.4 Proliferation assays

After counting and assessing cell viability, 1 x10^5 CD4$^+$ T cells were co-cultured with 1 x10^4 irradiated MoDC in 96 well round bottom plates. All cultures were set up in six replicate wells. Following a 7 day co-culture, the cells were pulsed with 1.0 µCi [^{3}H]-Thymidine (Perkin Elmer, Buckinghamshire, UK) in 50 µl AIM-V® medium and incubated for a further 6 hours before harvesting onto filter mats using a TomTec Mach III cell harvester. Cpm for each well were determined by Meltilex™ (Perkin Elmer) scintillation counting on a Microplate Beta Counter in paralux, low background counting.

3.5 ELISpot assays

ELISpot plates (Millipore, Watford, UK) were pre-wetted and coated overnight with 100 µl/well IL-2 capture antibody (R&D Systems, Abingdon, UK) in PBS. Plates were then washed 2 times in PBS, incubated overnight in blocking buffer (1% BSA in PBS) and washed in AIM-V® medium. CD4$^+$ T cells and DC were added to each well as for the proliferation assay (ratio 10:1).

Each sample was tested in sextuplet cultures and, for each donor, a negative control (AIM-V® medium alone), no cells control and a mitogen positive control (PHA at 2.5 µg/ml - used as an internal test for ELISpot function and cell viability, Sigma) were also included on each plate. After a 7 day incubation period, ELISpot plates were developed by sequential washing in dH_2O and PBS (x3) prior to the addition of 100 µl filtered, biotinylated detection antibody (R&D Systems) in PBS / 1% BSA. Following incubation at 37°C for 1.5 hours, plates were further washed in PBS (x3) and 100 µl filtered streptavidin-AP (R&D Systems) in PBS / 1% BSA was added for 1.5 hours (incubation at room temperature). Streptavidin-AP was discarded and

plates were washed in PBS (x4). 100 µl BCIP/NBT substrate (R&D Systems) was added to each well and incubated for 30 minutes at room temperature. Spot development was stopped by washing the wells and the backs of the wells three times with dH_2O. Dried plates were scanned on an Immunoscan® Analyser and spw were determined using Immunoscan® Version 5 software.

3.6 Assessment of cell viability

Following MoDC harvest on day 8, MoDC were assessed for viability using trypan blue dye exclusion. Viability was expressed as a percentage of cells unstained with trypan blue out of the total number of cells.

3.7 EpiScreen™ data analysis

For proliferation and IL-2 ELISpot assays, an empirical threshold of an SI equal to or greater than 1.90 (SI ≥1.90) has been previously established whereby samples inducing responses above this threshold are deemed positive. Extensive assay development and previous studies have shown that this is the minimum signal to noise threshold allowing maximum sensitivity without detecting large numbers of false positive responses or omitting subtle immunogenic events. For both proliferation (n=6) and IL-2 ELISpot (n=6) data sets, positive responses were defined by statistical and empirical thresholds:

1. Significance (p <0.05) of the response by comparing cpm or spw of test wells against medium control wells (cpm >150, spw >3) using an unpaired two sample Student's t-test.
2. SI ≥1.90, where SI = mean of test wells (cpm or spw) / baseline (cpm or spw). Data presented in this way is indicated as SI ≥1.90, p <0.05.

In addition, intra-assay variation was assessed using Dixons Q test in combination with the CV and SD of the raw data from replicate cultures.

3.8 Statistical analysis

P values were calculated using an unpaired two sample Student's t-test in Prism 5 (GraphPad, La Jolla, USA).

4. Results and Discussion

Two samples provided by Cleveland Biolabs, Inc. were assessed using PBMC from a cohort of 50, HLA-typed, healthy donors in the EpiScreen™ DC:T cell assay to determine the relative risk of immunogenicity. MoDC were prepared to a semi-mature stage and incubated with the samples before full DC maturation was induced by stimulation with the pro-inflammatory cytokine TNF-α. It has previously been shown, using the EpiScreen™ DC:T cell assay system, that the addition of TNF-α induces the up-regulation of DC costimulatory molecules such as CD80, CD86 and MHC class II which define a mature DC phenotype. The neoantigen KLH was included as a control. Both samples were assessed at a final concentration of 0.3 µM based on Abzena's previous experience showing that this saturating concentration is sufficient to stimulate detectable protein-specific T cell responses. To assess the immunogenic potential of each sample, the EpiScreen™ DC:T cell assay uses two markers (IL-2 production and proliferation) to measure T cell activation. Whilst there is generally a good correlation between IL-2 production and proliferation after T cells have been activated, differences can sometimes still occur. This can be due to the kinetics of T cell responses in culture where transient proliferation responses can potentially be missed, particularly if the proliferation response occurs during the very early stages of the autologous T cell culture (i.e. before day 7). Alternatively, differences can be due to activation of specific T cell subsets that undergo limited proliferation. Since the IL-2 ELISpot assay comprises a membrane pre-coated with capture antibody which binds secreted cytokine during the entire incubation time, both early and late responses will be detected. Proliferation and IL-2 ELISpot assays have therefore been interpreted independently, and differences and similarities then highlighted between the respective assay data.

4.1 Assessment of cell viability

To exclude any direct toxic effects of the samples on MoDC, the viability of cells four days after the removal of the samples (day 8 of MoDC culture) was assessed by trypan blue dye exclusion. **Figure 3** shows the mean viabilities of cells from 10 donors. The samples did not affect cell viability since the mean viability of MoDC treated with medium alone was similar to that of MoDC treated with samples or control antigen (KLH), between 94% and 96%.

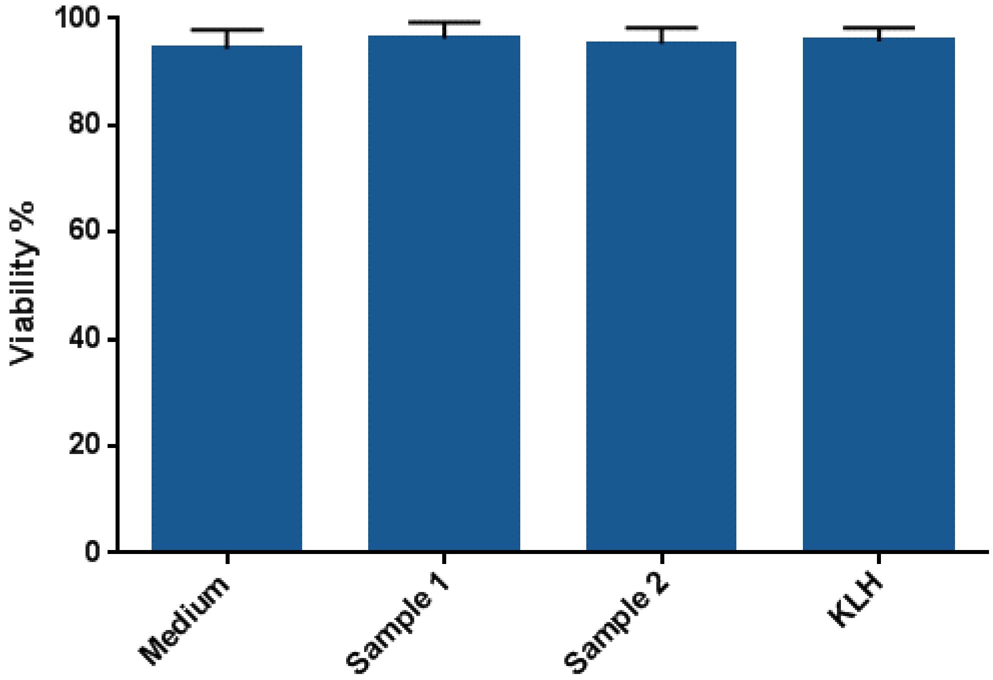

Figure 3. Viability of MoDC from 10 donors cultured with the samples and controls (medium and KLH) was assessed by trypan blue dye exclusion and is shown as an average with SD (error bars).

4.2 Screening of samples using EpiScreen™ DC:T cell assays

4.2.1 EpiScreen™ DC:T cell proliferation assay

Table 2 and **Figure 4** show the $CD4^+$ T cell proliferation in response to the samples. The neo-antigen KLH induced positive responses in 54% of the donor cohort, with a mean magnitude SI of 4.07. (**Table 3**).

Sample 1 induced positive responses in 28% of the donor cohort (SI ≥1.90 (p <0.05)), whereas sample 2 induced positive responses in 8% of the donor cohort. The mean magnitude of the positive T cell proliferation responses was low (SI <3.00) for both samples with mean SIs of 2.39 and 2.67 for sample 1 and 2 respectively (**Table 3**).

	Sample 1	Sample 2	KLH
Donor 1			
Donor 2			PE
Donor 3			
Donor 4			P
Donor 5			
Donor 6			
Donor 7			E
Donor 8			P
Donor 9	P		P
Donor 10	P	P	PE
Donor 11	PE	P	PE
Donor 12	P		PE
Donor 13	E	E	PE
Donor 14			
Donor 15			PE
Donor 16			
Donor 17		E	PE
Donor 18	P		PE
Donor 19			PE
Donor 20	P	P	PE
Donor 21	E		E
Donor 22	E	E	E
Donor 23			E
Donor 24	P	P	P
Donor 25			P
Donor 26	E	E	PE
Donor 27			
Donor 28			E
Donor 29	P		PE
Donor 30	P		PE
Donor 31	PE		PE
Donor 32			P
Donor 33			
Donor 34			PE
Donor 35	P		
Donor 36			
Donor 37	PE		E
Donor 38			
Donor 39	P		
Donor 40	E	E	E
Donor 41	PE		PE
Donor 42			PE
Donor 43			E
Donor 44			PE
Donor 45			E
Donor 46			PE
Donor 47			E
Donor 48			P
Donor 49	E		PE
Donor 50			
% Proliferation	28	8	54
% ELISpot	20	10	60
% Proliferation and ELISpot	8	0	40
% Correlation	29	0	74

Table 2. Summary of healthy donor T cell proliferation and IL-2 ELISpot responses. Positive T cell responses for proliferation (SI ≥1.90, p <0.05) ("P"), and IL-2 (SI ≥1.90, p <0.05) ELISpot ("E") after 7 days' culture are shown. The frequency of positive responses for proliferation and IL-2 ELISpot assays are shown as a percentage at the bottom of the columns. Correlation is expressed as the percentage of proliferation responses that were also positive in the IL-2 ELISpot assay.

Abzena ID	Mean SI	SD	% Response
Sample 1	2.39	±0.31	28
Sample 2	2.67	±0.98	8
KLH	4.07	±2.38	54

Table 3. Summary of the mean magnitude (±SD) of positive $CD4^{+}$ T cell proliferative responses against the samples. The mean SI was calculated from the average of positive donor responses observed during the assay.

(a) Sample 1

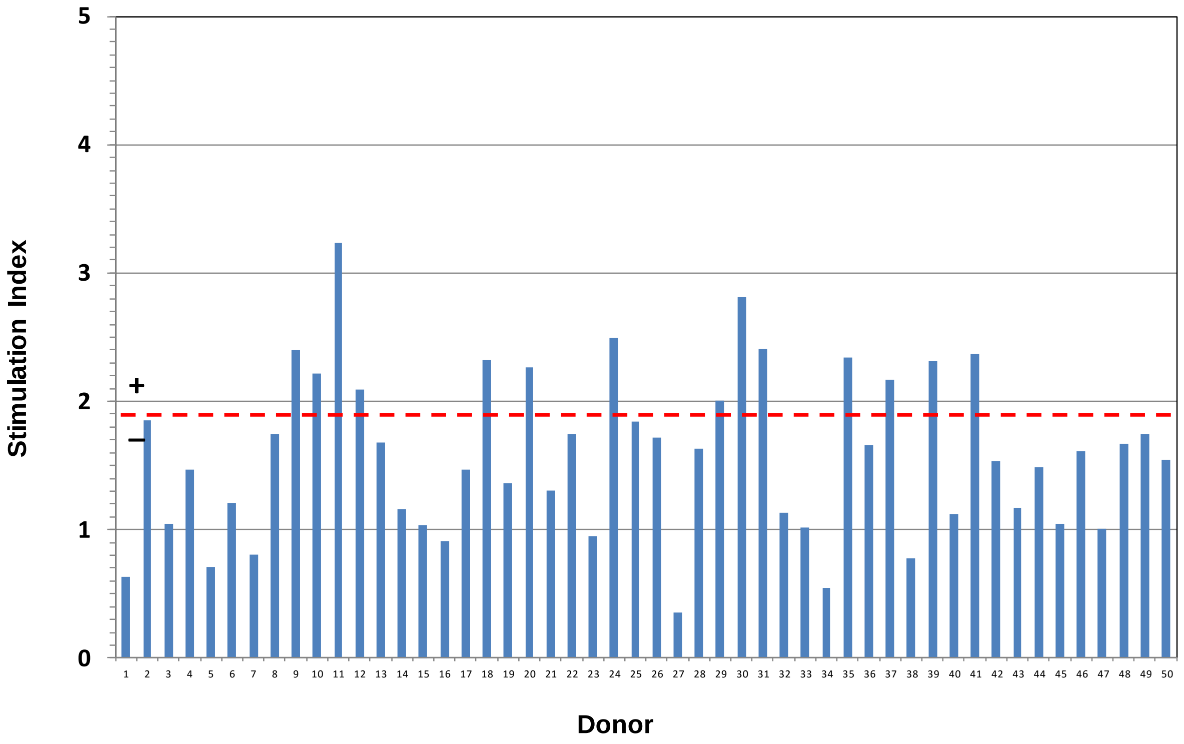

(b) Sample 2

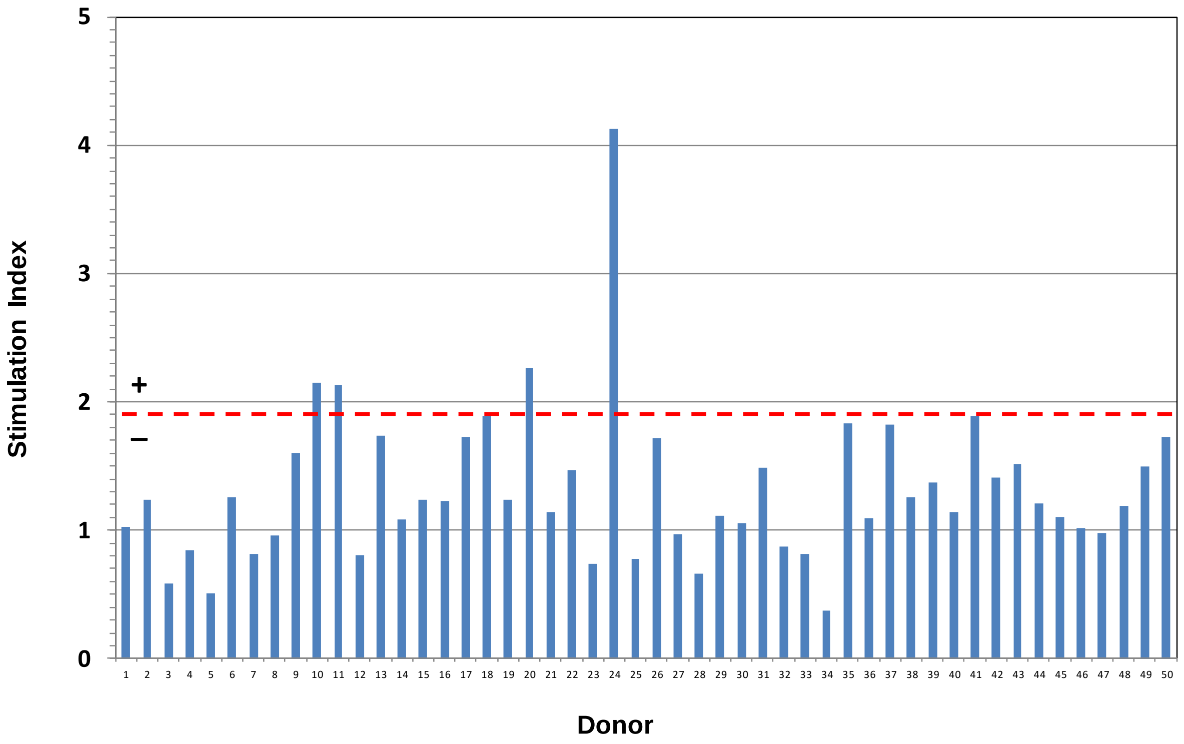

(c) KLH

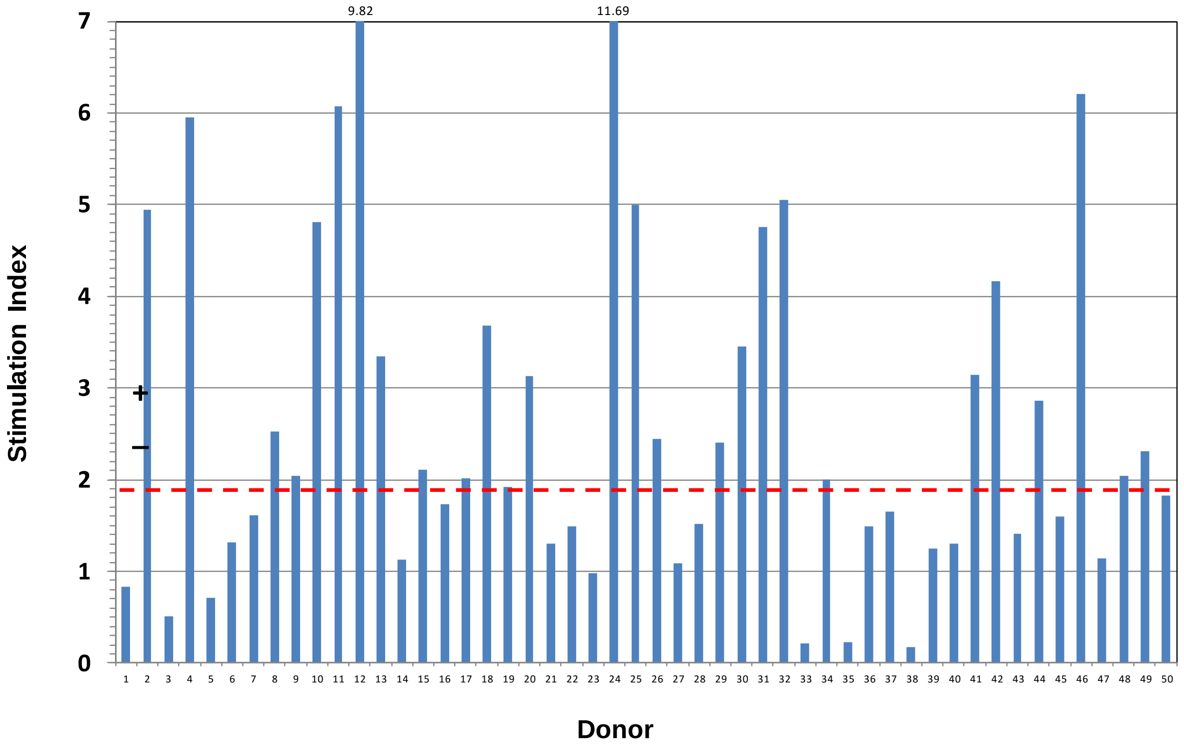

Figure 4. Healthy donor T cell proliferation responses to: (a) sample 1, (b) sample 2 and (c) KLH. CD4+ T cells were incubated with autologous mature DC loaded with the samples and assessed for proliferation after 7 days' incubation. T cell responses with an SI ≥1.90 (indicated by red dotted line) that were significant (p <0.05) using an unpaired, two sample Student's t-test were considered positive.

4.2.2 EpiScreen™ DC:T cell IL-2 ELISpot assay

Table 2 and **Figure 5** show the responses obtained in the IL-2 ELISpot assay which measures IL-2 secretion by CD4+ T cells following stimulation with DC loaded with the two samples and KLH. Similar to the proliferation assay, positive responses were recorded in donors that produced an SI ≥1.90 with a significant (p <0.05) difference observed between test spw and background (untreated medium control). All positive control PHA treated wells were positive for the presence of spots, although SI values are not prepared for the ELISpot data as after 7 days the majority of wells contained spots too numerous to count (data not shown).

KLH induced a positive response in 60% of donors with a mean magnitude SI of 3.82. The results obtained in the IL-2 ELISpot assay for the samples were similar to those obtained in the proliferation assay with sample 1 inducing a higher response rate. Samples 1 and 2 induced an IL-2 response frequency of 20% and 10% respectively and these were all significant (p <0.05) using an unpaired, two sample Student's t-test. The mean magnitude (SI) of the positive T cell responses in the IL-2 ELISpot assay for sample 1 was 2.92 and 3.12 for sample 2 (**Table 4**).

Abzena ID	Mean SI	SD	% Response
Sample 1	2.92	±1.44	20
Sample 2	3.12	±2.02	10
KLH	3.82	±3.07	60

Table 4. Summary of the frequency and magnitude (±SD) of positive IL-2 secretion responses against the two samples and KLH. The mean SI was calculated from all positive donor responses observed.

(a) Sample 1

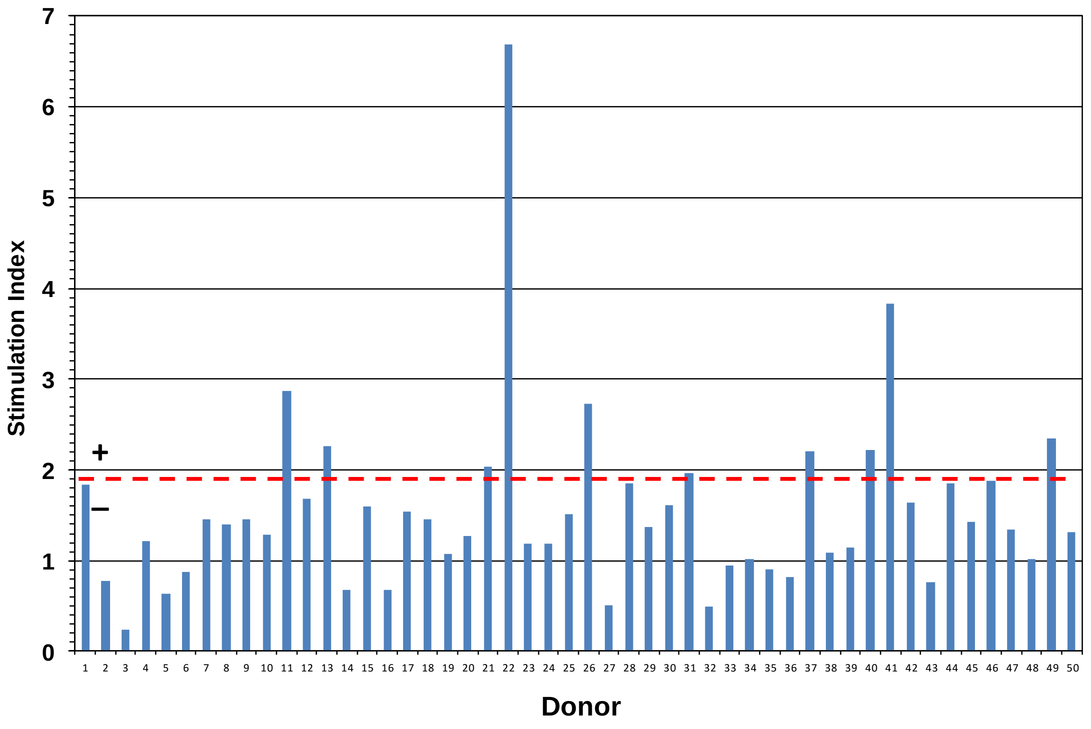

(b) Sample 2

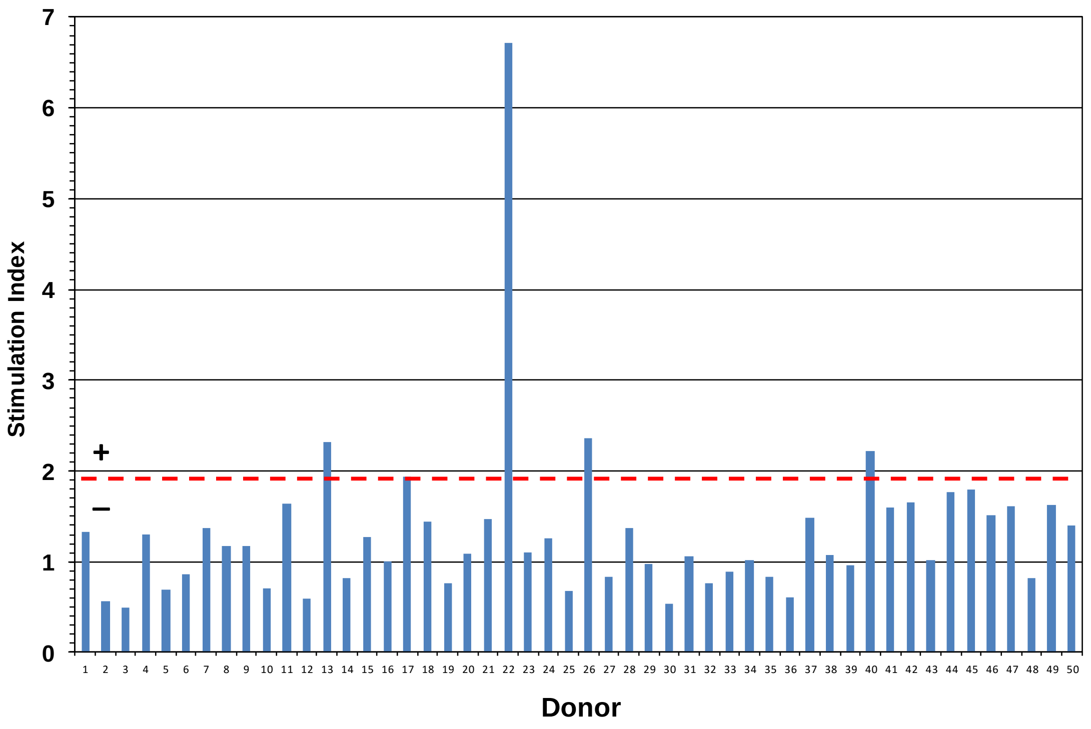

(b) KLH

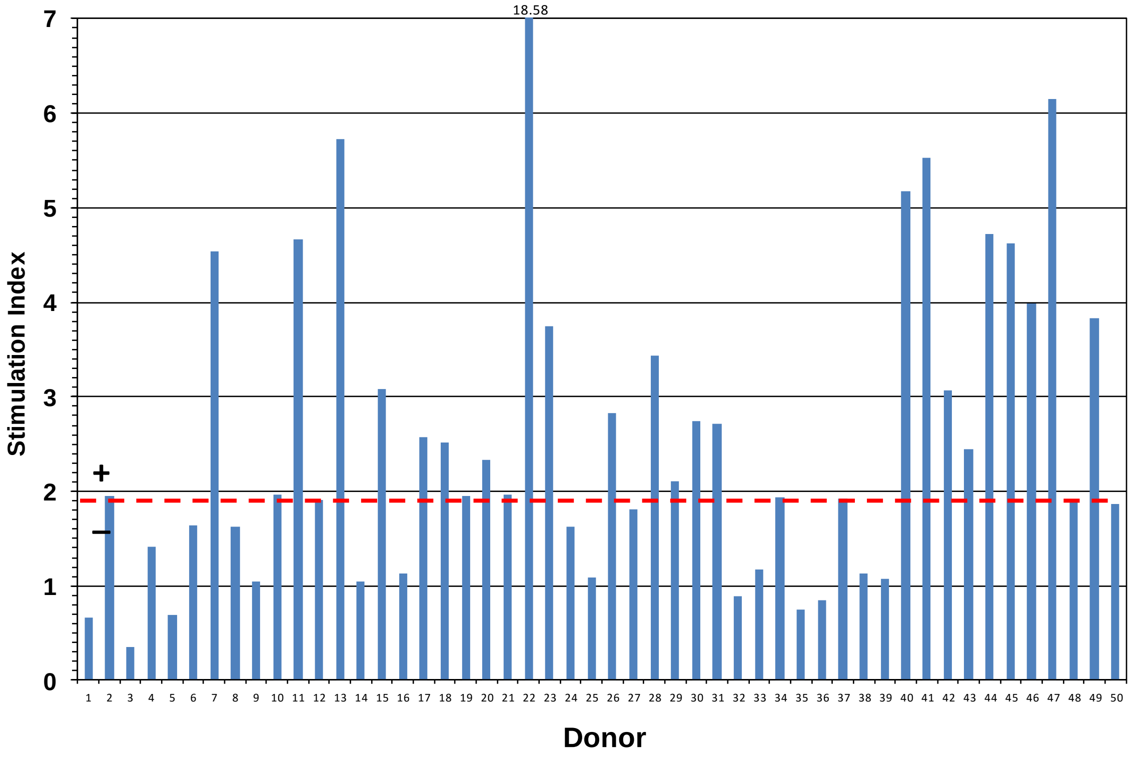

Figure 5. Healthy donor T cell IL-2 secretion response to: (a) sample 1, (b) sample 2 and (c) KLH. $CD4^+$ T cells were incubated with autologous mature DC loaded with the samples and assessed for IL-2 secretion after 7 days' incubation. T cell responses with an SI ≥1.90 (indicated by red dotted line) that were significant (p <0.05) using an unpaired, two sample Student's t-test were considered positive.

4.3 Interpretation of results

The overall correlation between proliferation and IL-2 ELISpot assays was 74% for KLH (**Table 2**) and thus, as in previous studies, responding donors were defined as those that mounted a positive response to a sample in both IL-2 ELISpot and proliferation assays. KLH yielded a combined frequency of positive responses of 40%, which is within the range of donor response observed across previous DC:T studies (25-55%) and was therefore considered valid. Moreover, all donors produced a positive T cell response against PHA in IL-2 ELISpot assays indicating that cells in the *ex vivo* cultures were functional (data not shown).

Analysis of the combined datasets from the two assays revealed that sample 1 and sample 2 gave combined frequency of positive responses of 8% and 0%, respectively.

To determine if there were any statistically significant differences in the magnitude of $CD4^+$ T cell responses to the samples in the proliferation and the IL-2 ELISpot data sets, *P* values were calculated using an unpaired two sample Student's t-test in Prism 6 (**Figure 6**). The magnitude of responses to sample 1 was significantly higher than responses to sample 2 in the proliferation assay. There were no significant differences between the samples in the IL-2 ELISpot assay.

Analysis of the responding donor haplotypes may be performed to determine if there was a strong association between MHC class II allotype and the capacity to induce a T cell response. This analysis can only be carried out if samples induced responses in both the proliferation assay and the ELISpot assay in >4 donors in the study cohort, and is restricted to allotypes expressed at higher frequencies (>5%) in the whole study population. Neither sample response rate reached the criteria necessary to carry out this analysis.

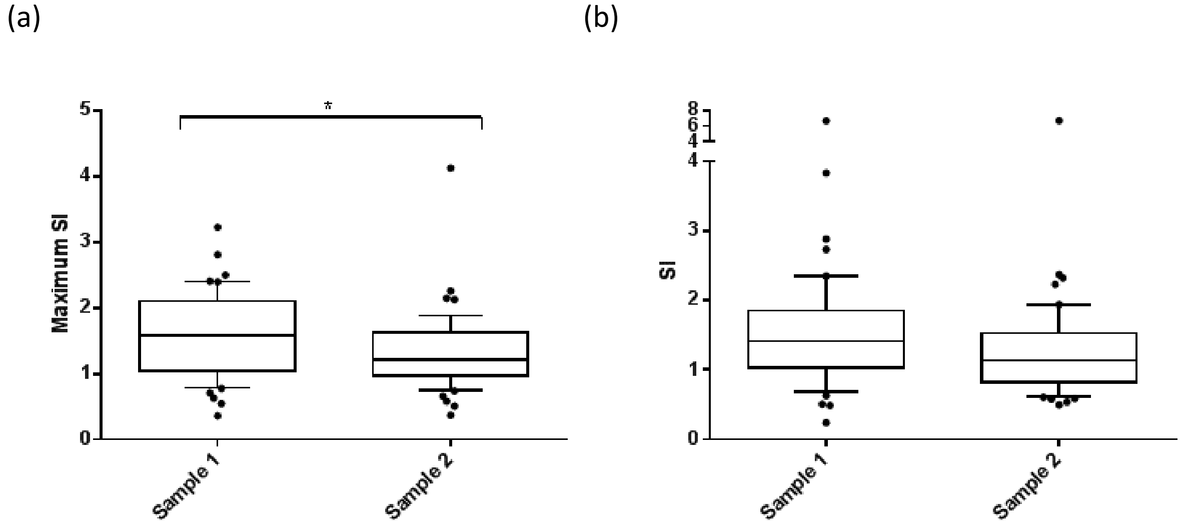

Figure 6. Box and whisker plots showing healthy donor T cell responses to the samples: (a) Proliferation of CD4^{+} T cells and (b) T cell IL-2 ELISpot responses. Bars represent the 10-90 percentile. * $p < 0.05$

5. Conclusion

The EpiScreen™ DC:T cell assay was used to determine the relative potential for clinical immunogenicity of two samples. The ability of mature DC, loaded with the samples, to induce $CD4^+$ T cell responses in PBMC from a panel of 50 HLA-typed donors was measured by proliferation and IL-2 ELISpot. The results showed that both samples induced a combined positive response frequency in 0-8% of the donor cohort. However, the correlation between positive proliferation and IL-2 ELISpot responses for the test samples was low. In the individual assays sample 1 would be considered of greater risk of clinical immunogenicity than sample 2 due to the high frequency of positive proliferation (28% vs 8%) and IL-2 ELISpot (20% vs 10%) responses. In addition, the mean magnitude of proliferative responses to sample 1 was significantly higher than those to sample 2, adding further evidence to support the conclusion of an increased risk of clinical immunogenicity for sample 1 versus sample 2.

6. References

Baker, M.P. & Jones, T.D., 2007. Identification and removal of immunogenicity in therapeutic proteins. *Current opinion in drug discovery & development*, 10(2), pp.219–27.

Chester, K.A., Baker, M. & Mayer, A., 2005. Overcoming the immunologic response to foreign enzymes in cancer therapy. *Expert review of clinical immunology*, 1(4), pp.549–59.

Edwards, J.A. et al., 1986. Differential expression of HLA class II antigens in fetal human spleen: relationship of HLA-DP, DQ, and DR to immunoglobulin expression. *Journal of immunology (Baltimore, Md. : 1950)*, 137(2), pp.490–7.

Hochuli, E., 1997. Interferon immunogenicity: technical evaluation of interferon-alpha 2a. *Journal of interferon & cytokine research : the official journal of the International Society for Interferon and Cytokine Research*, 17 Suppl 1, pp.S15–21.

Jaber, A. & Baker, M., 2007. Assessment of the immunogenicity of different interferon beta-1a formulations using ex vivo T-cell assays. *Journal of pharmaceutical and biomedical analysis*, 43(4), pp.1256–61.

Jones, T.D. et al., 2005. Identification and removal of a promiscuous CD4+ T cell epitope from the C1 domain of factor VIII. *Journal of thrombosis and haemostasis : JTH*, 3(5), pp.991–1000.

Jones, T.D. et al., 2004. The development of a modified human IFN-alpha2b linked to the Fc portion of human IgG1 as a novel potential therapeutic for the treatment of hepatitis C virus infection. *Journal of interferon & cytokine research : the official journal of the International Society for Interferon and Cytokine Research*, 24(9), pp.560–72.

Lim, L.C., 2005. Acquired red cell aplasia in association with the use of recombinant erythropoietin in chronic renal failure. *Hematology (Amsterdam, Netherlands)*, 10(3), pp.255–9.

Namaka, M. et al., 2006. The clinical importance of neutralizing antibodies in relapsing-remitting multiple sclerosis. *Current medical research and opinion*, 22(2), pp.223–39.

Schellekens, H., Ryff, J.C. & van der Meide, P.H., 1997. Assays for antibodies to human interferon-alpha: the need for standardization. *Journal of interferon & cytokine research : the official journal of the International Society for Interferon and Cytokine Research*, 17 Suppl 1, pp.S5–8.

Appendix I

	High resolution haplotype of donors
Donor 1	DRB1*07:01:01:01;DRB1*15:01:01:01;DRB4*01:03:01:01;DRB5*01:01:01;DQB1*02:02:01;DQB1*06:02:01
Donor 2	DRB1*01:01:01;DRB1*13:01:01:01;DRB3*02:02:01:01;DQB1*05:01:01:01;DQB1*06:03:01:01
Donor 3	DRB1*03:01:01:01;DRB1*08:01:01;DRB3*01:01:02:01;DQB1*02:01:01;DQB1*04:02:01
Donor 4	DRB1*07:01:01:01;DRB4*01:03:01:02N;DQB1*03:03:02:01
Donor 5	DRB1*15:02:01:01;DRB5*01:02;DQB1*05:03:01:01
Donor 6	DRB1*13:01:01:01;DRB1*13:02:01;DRB3*01:01:02:01;DRB3*03:01:01;DQB1*06:03:01:01;DQB1*06:04:01
Donor 7	DRB1*11:04:01;DRB1*14:04:01;DRB3*02:02:01:01;DQB1*03:01:01:01;DQB1*05:03:01:01
Donor 8	DRB1*15:01:01:01;DRB5*01:01:01;DQB1*06:02:01:01
Donor 9	DRB1*15:02:01:01;DRB5*01:02;DQB1*06:01:01
Donor 10	DRB1*03:01:01:01;DRB1*13:02:01;DRB3*01:01:02:01;DRB3*03:01:01;DQB1*02:01:01;DQB1*06:04:01
Donor 11	DRB1*07:01:01:01;DRB1*08:01:01;DRB4*01:03:01:02N;DQB1*03:03:02:01;DQB1*04:02:01
Donor 12	DRB1*03:01:01:01;DRB1*15:01:01:01;DRB3*01:01:02:01;DRB5*01:01:01;DQB1*02:01:01;DQB1*06:02:01
Donor 13	DRB1*03:01:01:01;DRB1*16:02:01;DRB3*01:01:02:01;DRB5*02:02;DQB1*02:01:01;DQB1*05:02:01
Donor 14	DRB1*01:01:01;DRB1*03:01:01:01;DRB3*01:01:02:01;DQB1*02:01:01;DQB1*05:01:01:01
Donor 15	DRB1*13:02:01;DRB1*15:01:01:01;DRB3*03:01:01;DRB5*01:01:01;DQB1*06:02:01;DQB1*06:04:01
Donor 16	DRB1*03:01:01:01;DRB1*11:04:01;DRB3*02:02:01:01;DQB1*02:01:01;DQB1*03:01:01:01
Donor 17	DRB1*11:01:01;DRB1*11:04:01;DRB3*02:02:01:01;DQB1*03:01:01:01
Donor 18	DRB1*01:01:01;DRB1*16:01:01;DRB5*02:02;DQB1*05:01:01:01;DQB1*05:02:01
Donor 19	DRB1*04:02:01;DRB1*16:01:01;DRB4*01:03:01:01;DRB5*02:02;DQB1*03:02:01;DQB1*05:02:01
Donor 20	DRB1*03:01:01:01;DRB1*08:04:01;DRB3*01:01:02:01;DQB1*02:01:01;DQB1*04:02:01
Donor 21	DRB1*01:01:01;DRB1*15:01:01:01;DRB5*01:01:01;DQB1*05:01:01:01;DQB1*06:02:01
Donor 22	DRB1*11:04:01;DRB1*13:01:01;DRB3*01:01:02:01;DRB3*02:02:01:01;DQB1*03:01:01:01;DQB1*06:03:01
Donor 23	DRB1*03:01:01:01;DRB1*04:04:01;DRB3*01:01:02:01;DRB4*01:03:01:01;DQB1*02:01:01;DQB1*03:02:01
Donor 24	DRB1*07:01:01:01;DRB1*14:04:01;DRB3*02:02:01:01;DRB4*01:03:01:02N;DQB1*03:03:02:01; DQB1*05:03:01:01

Donor 25	DRB1*01:01:01;DRB1*04:04:01;DRB4*01:03:01:01;DQB1*03:02:01;DQB1*05:01:01:01
Donor 26	DRB1*03:01:01:01;DRB1*07:01:01:01;DRB3*01:01:02:01;DRB4*01:03:01:01;DQB1*02:01:01; DQB1*02:02:01:01
Donor 27	DRB1*03:01:01:01;DRB1*11:01:01;DRB3*01:01:02:01;DRB3*02:02:01:01;DQB1*02:01:01; DQB1*03:01:01:01
Donor 28	DRB1*01:01:01;DRB1*11:04:01;DRB3*02:02:01:01;DQB1*03:01:01:01;DQB1*05:01:01:01
Donor 29	DRB1*07:01:01:01;DRB1*15:01:01:01;DRB4*01:03:01:02N;DRB5*01:01:01;DQB1*03:03:02:01; DQB1*06:02:01
Donor 30	DRB1*13:02:01;DRB1*14:01:01/14:54:01;DRB3*02:02:01:01;DRB3*03:01:01;DQB1*05:03:01:01; DQB1*06:09:01:01
Donor 31	DRB1*07:01:01:01;DRB1*13:02:01;DRB3*03:01:01;DRB4*01:03:01:01;DQB1*02:02:01:01;DQB1*06:04:01
Donor 32	DRB1*03:01:01:01;DRB3*01:01:02:01;DQB1*02:01:01
Donor 33	DRB1*03:01:01:01;DRB1*11:04:01;DRB3*02:02:01:01;DQB1*02:01:01;DQB1*03:01:01:01
Donor 34	DRB1*03:01:01:01;DRB1*11:01:01:01;DRB3*01:01:02:01;DRB3*02:02:01:01;DQB1*02:01:01; DQB1*03:01:01:01
Donor 35	DRB1*04:04:01;DRB1*11:01:01:01;DRB3*02:02:01:01;DRB4*01:03:01:01;DQB1*03:01:01:01; DQB1*03:02:01:01
Donor 36	DRB1*13:01:01:01;DRB1*16:02:01:01;DRB3*02:02:01:01;DRB5*02:02:01;DQB1*05:02:01:01; DQB1*06:03:01:01
Donor 37	DRB1*03:01:01:01;DRB1*14:01:01/14:54:01;DRB3*02:02:01:01;DQB1*02:01:01;DQB1*05:03:01:01
Donor 38	DRB1*11:04:01;DRB3*02:02:01:01;DQB1*03:01:01:01
Donor 39	DRB1*01:01:01;DRB1*15:01:01:01;DRB5*01:01:01;DQB1*05:01:01:01;DQB1*06:02:01:01
Donor 40	DRB1*04:05:01;DRB1*11:04:01;DRB3*02:02:01:01;DRB4*01:03:01:01;DQB1*02:02:01:01; DQB1*03:01:01:01
Donor 41	DRB1*03:01:01:01;DRB1*15:01:01:01;DRB3*01:01:02:01;DRB5*01:01:01;DQB1*02:01:01; DQB1*06:02:01:01
Donor 42	DRB1*08:01:01;DRB1*15:02:01:01;DRB5*01:02;DQB1*04:02:01:01;DQB1*06:01:01
Donor 43	DRB1*07:01:01:01;DRB1*11:01:01:01;DRB3*02:02:01:01;DRB4*01:03:01:01;DQB1*02:02:01:01; DQB1*03:01:01:01
Donor 44	DRB1*11:01:01:01;DRB1*16:01:01;DRB3*02:02:01:01;DRB5*02:02:01;DQB1*03:01:01:01; DQB1*05:02:01:01
Donor 45	DRB1*04:04:01;DRB1*11:04:01;DRB3*02:02:01:01;DRB4*01:03:01:01;DQB1*03:01:01:01; DQB1*03:02:01:01
Donor 46	DRB1*08:01:01;DRB1*15:01:01:01;DRB5*01:01:01;DQB1*04:02:01:01;DQB1*06:02:01:01
Donor 47	DRB1*01:01:01;DRB1*10:01:01:01;DQB1*05:01:01:01
Donor 48	DRB1*01:01:01;DRB1*14:01:01/14:54:01:01;DRB3*02:02:01:01;DQB1*05:01:01:01;DQB1*05:03:01:01
Donor 49	DRB1*07:01:01:01;DRB4*01:01:01:01;DRB4*01:03:01:01;DQB1*02:02:01:01
Donor 50	DRB1*03:01:01:01;DRB1*15:01:01:01;DRB3*02:02:01:01;DRB5*01:01:01;DQB1*02:01:01; DQB1*06:02:01:01

Appendix II

Proliferation assay SI for donors 1 to 50. Numbers in red indicate positive responses (SI ≥1.90, p <0.05).

	Sample 1	Sample 2	KLH
Donor 1	0.63	1.02	0.83
Donor 2	1.85	1.24	4.95
Donor 3	1.04	0.58	0.51
Donor 4	1.47	0.85	5.95
Donor 5	0.71	0.51	0.71
Donor 6	1.21	1.25	1.31
Donor 7	0.81	0.82	1.61
Donor 8	1.74	0.95	2.52
Donor 9	2.40	1.60	2.04
Donor 10	2.22	2.15	4.81
Donor 11	3.23	2.13	6.08
Donor 12	2.09	0.81	9.82
Donor 13	1.68	1.74	3.34
Donor 14	1.17	1.08	1.13
Donor 15	1.03	1.24	2.11
Donor 16	0.91	1.23	1.73
Donor 17	1.47	1.73	2.02
Donor 18	2.33	1.89	3.67
Donor 19	1.36	1.23	1.92
Donor 20	2.27	2.26	3.13
Donor 21	1.31	1.14	1.30
Donor 22	1.75	1.47	1.49
Donor 23	0.95	0.74	0.97
Donor 24	2.50	4.13	11.69
Donor 25	1.85	0.78	5.00
Donor 26	1.72	1.72	2.45
Donor 27	0.36	0.97	1.08
Donor 28	1.63	0.66	1.52
Donor 29	2.01	1.11	2.41
Donor 30	2.81	1.06	3.46
Donor 31	2.41	1.49	4.76
Donor 32	1.13	0.87	5.06
Donor 33	1.01	0.81	0.22
Donor 34	0.55	0.37	2.00
Donor 35	2.35	1.83	0.23
Donor 36	1.66	1.10	1.49
Donor 37	2.17	1.83	1.65
Donor 38	0.78	1.25	0.17
Donor 39	2.31	1.37	1.24
Donor 40	1.12	1.14	1.30
Donor 41	2.37	1.89	3.15
Donor 42	1.53	1.41	4.16
Donor 43	1.17	1.51	1.40
Donor 44	1.49	1.21	2.86
Donor 45	1.04	1.11	1.60
Donor 46	1.61	1.02	6.20
Donor 47	1.01	0.98	1.15
Donor 48	1.67	1.19	2.04
Donor 49	1.74	1.49	2.32
Donor 50	1.54	1.73	1.83

Appendix III

IL-2 ELISpot assay SI for donors 1 to 50. Numbers in red indicate positive responses (SI ≥1.90, p <0.05).

	Sample 1	Sample 2	KLH
Donor 1	1.83	1.33	0.67
Donor 2	1.03	0.58	1.95
Donor 3	0.24	0.50	0.36
Donor 4	1.21	1.30	1.41
Donor 5	0.63	0.69	0.69
Donor 6	0.88	0.87	1.65
Donor 7	1.46	1.38	4.53
Donor 8	1.40	1.17	1.62
Donor 9	1.45	1.17	1.05
Donor 10	1.29	0.71	1.96
Donor 11	2.88	1.65	4.67
Donor 12	1.68	0.59	1.91
Donor 13	2.26	2.32	5.73
Donor 14	0.68	0.82	1.04
Donor 15	1.59	1.27	3.08
Donor 16	0.68	1.00	1.14
Donor 17	1.55	1.94	2.57
Donor 18	1.46	1.44	2.52
Donor 19	1.07	0.77	1.95
Donor 20	1.27	1.09	2.33
Donor 21	2.04	1.47	1.97
Donor 22	6.69	6.72	18.58
Donor 23	1.18	1.10	3.75
Donor 24	1.18	1.26	1.62
Donor 25	1.51	0.68	1.09
Donor 26	2.73	2.37	2.83
Donor 27	0.51	0.83	1.81
Donor 28	1.85	1.37	3.44
Donor 29	1.46	1.05	2.11
Donor 30	1.61	0.54	2.74
Donor 31	1.97	1.06	2.71
Donor 32	0.49	0.76	0.89
Donor 33	0.94	0.89	1.17
Donor 34	1.07	1.07	1.93
Donor 35	0.91	0.83	0.75
Donor 36	0.82	0.61	0.85
Donor 37	2.21	1.48	1.93
Donor 38	1.09	1.07	1.13
Donor 39	1.14	0.96	1.08
Donor 40	2.23	2.23	5.18
Donor 41	3.83	1.59	5.53
Donor 42	1.64	1.65	3.06
Donor 43	0.76	1.01	2.45
Donor 44	1.85	1.76	4.72
Donor 45	1.42	1.79	4.62
Donor 46	1.88	1.51	3.99
Donor 47	1.35	1.61	6.15
Donor 48	1.02	0.82	1.89
Donor 49	2.35	1.62	3.83
Donor 50	1.31	1.40	1.87
