# Supplementary material for: A deimmunized and pharmacologically optimized Toll-like receptor 5 agonist for therapeutic applications
Source: Commun Biol. 2021 Apr 12;4:466. doi: 10.1038/s42003-021-01978-6 (PMC8041767; doi:10.1038/s42003-021-01978-6)
Supplement: Supplementary file 3 — Description of Additional Supplementary Files [file 42003_2021_1978_MOESM3_ESM.pdf]

Description of Additional Supplementary Files

File name: Supplementary Data 1

Description: Source data for graphs shown in the paper (Figures 1, 3b, 5, 6, 9 and 10).
