# Supplementary material for: A deimmunized and pharmacologically optimized Toll-like receptor 5 agonist for therapeutic applications
Source: Commun Biol. 2021 Apr 12;4:466. doi: 10.1038/s42003-021-01978-6 (PMC8041767; doi:10.1038/s42003-021-01978-6)
Supplement: Supplementary file 5 — Reporting Summary [file 42003_2021_1978_MOESM5_ESM.pdf]

Corresponding author(s): Andrei Gudkov

Last updated by author(s): Feb 26, 2021

Reporting Summary

Nature Research wishes to improve the reproducibility of the work that we publish. This form provides structure for consistency and transparency in reporting. For further information on Nature Research policies, see our Editorial Policies and the Editorial Policy Checklist.

Statistics

For all statistical analyses, confirm that the following items are present in the figure legend, table legend, main text, or Methods section.

n/a	Confirmed	
☐	☒	The exact sample size (n) for each experimental group/condition, given as a discrete number and unit of measurement
☐	☒	A statement on whether measurements were taken from distinct samples or whether the same sample was measured repeatedly
☐	☒	The statistical test(s) used AND whether they are one- or two-sided<br>*Only common tests should be described solely by name; describe more complex techniques in the Methods section.*
☒	☐	A description of all covariates tested
☒	☐	A description of any assumptions or corrections, such as tests of normality and adjustment for multiple comparisons
☐	☒	A full description of the statistical parameters including central tendency (e.g. means) or other basic estimates (e.g. regression coefficient) AND variation (e.g. standard deviation) or associated estimates of uncertainty (e.g. confidence intervals)
☐	☒	For null hypothesis testing, the test statistic (e.g. F, t, r) with confidence intervals, effect sizes, degrees of freedom and P value noted<br>*Give P values as exact values whenever suitable.*
☒	☐	For Bayesian analysis, information on the choice of priors and Markov chain Monte Carlo settings
☒	☐	For hierarchical and complex designs, identification of the appropriate level for tests and full reporting of outcomes
☒	☐	Estimates of effect sizes (e.g. Cohen's d, Pearson's r), indicating how they were calculated

Our web collection on statistics for biologists contains articles on many of the points above.

Software and code

Policy information about availability of computer code

Data collection	For quantification of TLR5 agonist activity in transgenic NF-kB-luciferase reporter mice, bioluminescence (total flux) was measured using Living Image software (Perkin Elmer).<br>For RNAseq-based analysis of gene expression, sequencing data quality was assessed via FastQC and reads were aligned to the mouse reference genome with STAR RNA-seq aligner using annotation from the same source. Reads were counted using featureCounts using the same annotation. Differential gene expression and normalized counts were calculated using DESeq2. References for these tools are provided in the manuscript.
Data analysis	In our RNAseq experiment, differential gene expression comparison, analysis and visualization were performed using both Python and R programming languages. KEGG enrichment was performed with R clusterProfiler package (reference provided in the manuscript). All other data analysis was performed using GraphPad Prism software.

For manuscripts utilizing custom algorithms or software that are central to the research but not yet described in published literature, software must be made available to editors and reviewers. We strongly encourage code deposition in a community repository (e.g. GitHub). See the Nature Research guidelines for submitting code & software for further information.

Data

Policy information about availability of data

All manuscripts must include a data availability statement. This statement should provide the following information, where applicable:

- Accession codes, unique identifiers, or web links for publicly available datasets
- A list of figures that have associated raw data
- A description of any restrictions on data availability

The authors declare that all data supporting this study are available within the article and its supplementary information files, may be obtained from the

corresponding author upon reasonable request, or in the case of our RNAseq experiment comparing gene expression in livers of wild type and TLR5-/- C57BL/6 mice treated with vehicle, entolimod or GP532, are available in NCBI's Gene Expression Omnibus (GEO) database under accession number GSE163748 (https://www.ncbi.nlm.nih.gov/geo/query/acc.cgi?acc=GSE163748). Source data for graphs presented in the article (Figures 1, 3b, 5, 6, 9 and 10) are provided in the Supplementary Data file.

Field-specific reporting

Please select the one below that is the best fit for your research. If you are not sure, read the appropriate sections before making your selection.

- [x] Life sciences
- [ ] Behavioural & social sciences
- [ ] Ecological, evolutionary & environmental sciences

For a reference copy of the document with all sections, see nature.com/documents/nr-reporting-summary-flat.pdf

Life sciences study design

All studies must disclose on these points even when the disclosure is negative.

Sample size	In all animal experiments, sample sizes were chosen to provide statistical significance for the expected magnitude of the effect based on preliminary (pilot) and/or similar previous studies.
Data exclusions	No data were excluded.
Replication	Replicates were included in every experiment.
Randomization	Not applicable to our studies.
Blinding	The histology scoring of radiation-induced tissue damage was performed blindly.

Reporting for specific materials, systems and methods

We require information from authors about some types of materials, experimental systems and methods used in many studies. Here, indicate whether each material, system or method listed is relevant to your study. If you are not sure if a list item applies to your research, read the appropriate section before selecting a response.

Materials & experimental systems

n/a	Involved in the study	
[ ]	[x]	Antibodies
[ ]	[x]	Eukaryotic cell lines
[x]	[ ]	Palaeontology and archaeology
[ ]	[x]	Animals and other organisms
[x]	[ ]	Human research participants
[ ]	[x]	Clinical data
[x]	[ ]	Dual use research of concern

Methods

n/a	Involved in the study	
[x]	[ ]	ChIP-seq
[x]	[ ]	Flow cytometry
[x]	[ ]	MRI-based neuroimaging

Antibodies

Antibodies used	Human serum samples from 45 anonymous entolimod-naïve donors, selected for high entolimod neutralization activity (Bioreclamation IVT)<br>Serum samples from patients injected with entolimod in clinical trials (Cleveland BioLabs, Inc.)<br>Entolimod-reactive mouse monoclonal antibodies mAB4D11 and mAb11D04 (Cleveland BioLabs, Inc.)<br>Normal human serum samples with very low levels of entolimod-reactive neutralizing antibodies (obtained from clinical trial subjects prior to entolimod administration; Cleveland BioLabs, Inc.)<br>Rabbit monoclonal Ab against NF-κB p65 (Cell Signaling, catalog #8242)<br>Rat monoclonal Ab against macrophage marker F4/80 (ThermoFisher, catalog #MA1-91124)<br>Fluorochrome-conjugated donkey Abs from Johnson ImmunoResearch (anti-rabbit Cy3 and anti-rat AlexaFluor 488)
Validation	All entolimod-recognizing antibodies and antisera listed above were validated using ELISA and/or neutralization of in vitro signaling as described in the manuscript. Commercial antibodies used for IHC were validated by staining of control tissues.

Eukaryotic cell lines

Policy information about cell lines

Cell line source(s)	HEK293-hTLR5::NF-kB-lacZ reporter cells (InvivoGen)<br>THP1-NLRC4 cells (InvivoGen)
Authentication	Commercial cell lines were purchased with Certificate of Analysis and used without further authentication.
Mycoplasma contamination	Commercial cell lines were obtained mycoplasma-free from the vendor and either used fresh or propagated in the lab with regular mycoplasma testing (every 1-2 months).
Commonly misidentified lines<br>(See ICLAC register)	None

Animals and other organisms

Policy information about studies involving animals; ARRIVE guidelines recommended for reporting animal research

Laboratory animals	Mus musculus:<br>-female C57BL/6 mice (9-12 weeks old)<br>-male and female transgenic NF-kB reporter mice (Balb/C-Tg(IκBα-luc)Xen; 14-23 weeks old)<br>-male C57BL/6 mice (12 weeks old)<br>-male TLR5-knockout mice (B6(Cg)-Tlr5tm1.2Gewr/J; 12 weeks old)<br>-female NIH Swiss mice (12 weeks old)<br>-female BALB/c mice (13 weeks old)<br>-female FVB/NJ mice (10-12 weeks old)
Wild animals	N/A
Field-collected samples	N/A
Ethics oversight	Experiments performed in Mus musculus (laboratory mice) complied with all relevant ethical regulations for animal testing and research and were approved by the Institutional Animal Care and Use Committee (IACUC) of Roswell Park Cancer Institute (RPCI).

Note that full information on the approval of the study protocol must also be provided in the manuscript.

Clinical data

Policy information about clinical studies

All manuscripts should comply with the ICMJEguidelines for publication of clinical research and a completedCONSORT checklist must be included with all submissions.

Clinical trial registration	NCT01527136 (ClinicalTrials.gov)
Study protocol	The full protocol can be obtained from Cleveland BioLabs, Inc. (CBLI)
Data collection	The study was performed by CBLI at Roswell Park Cancer Institute (RPCI, Buffalo, NY) under RPCI Study Number I196111 (Alex Adjei, MD, PhD, Principal Investigator). The study protocol was approved by the Institutional Review Board of RPCI and followed all relevant ethical regulations. Informed consent for the study, including collection of blood samples for measurement of anti-entolimod antibody levels, was obtained from all trial participants prior to their enrollment. Start date: January 2012; Completion date: October 2014.
Outcomes	(i) MTD of entolimod [Time Frame: 3 weeks] and (ii) safety of entolimod [Time Frame: up to 30 days post-treatment]; both evaluated using National Cancer Institute (NCI)-Common Terminology Criteria for Adverse Events (CTCAE) Version 4.0.
